# Transition-metal-free one-pot synthesis of alkynyl selenides from terminal alkynes under aerobic and sustainable conditions

**DOI:** 10.3762/bjoc.13.92

**Published:** 2017-05-16

**Authors:** Adrián A Heredia, Alicia B Peñéñory

**Affiliations:** 1 INFIQC, Facultad de Ciencias Químicas, Universidad Nacional de Córdoba, Ciudad Universitaria, X5000HUA Córdoba, Argentina

**Keywords:** alkynyl selenide, electrophilic cyclization, nucleophilic substitution, potassium selenocyanate, terminal alkynes

## Abstract

Alkynyl selenides were synthesized by a straightforward one-pot and three-step methodology, without the need of diselenides as starting reagents, under an oxygen atmosphere and using PEG 200 as the solvent. This procedure involves the in situ generation of dialkyl diselenides through a K_3_PO_4_-assisted reaction of an alkyl selenocyanate obtained by a nucleophilic substitution reaction between KSeCN and alkyl halides. Successive reaction with terminal alkynes in the presence of *t-*BuOK affords the corresponding alkyl alkynyl selenide in moderate to good yields. Finally, this methodology allowed the synthesis of 2-alkylselanyl-substituted benzofuran and indole derivatives starting from convenient 2-substituted acetylenes.

## Introduction

Alkynyl selenides, as many other selenium compounds, have potential anti-oxidant activities, and may play a role in certain diseases such as cancer, heart diseases, inflammatory processes, arthritis, and skin damage caused by exposure to UV radiation [1–3]. However, all these properties belong to organoselenides due to their ability to engage in enzymatic processes [4–5] and alkynyl selenides have demonstrated only limited biological properties [6–7]. On the other hand, these compounds are widely applied in organic chemistry due to the presence of an alkynyl group [8–9] and an organoselenium moiety [10] in one molecule both comprising versatile building blocks or intermediates in organic synthesis. Some of the most outstanding synthetic applications of alkynyl selenides are electrophilic addition reactions, many of them modulated by transition-metal catalysts [11]. Some examples are: hydroboration which results in a vinylborane selenide used in Pd-catalyzed Suzuki cross-coupling reactions [12], the addition to tributyltin hydride in the presence of Pd and Cu catalysts to afford (*E*)-α-selenylstannanes for the synthesis of trisubstituted alkenes [13] and hydrozirconation with further replacement of Zr atom by hydrogen or halogen [14] or their use in the preparation of α-seleno-α,β-unsaturated ketones [15]. Besides, hydrogen halide-addition reactions to alkynyl selenides in the absence of transition metals [16] and the addition of *p-*toluenesulfonic acid [17] are applied to obtain vinyl organometallic compounds and key selenoester intermediates. Finally, electrophilic cyclizations [18–19] of systems bearing a selenide alkynyl group allow the synthesis of seleno-functionalized heterocycles [20–21]. Recently, the use of alkynyl selenides as substrates for Pd-catalyzed Suzuki, Negishi, Kumada and Sonogashira cross-coupling reactions has been reported with good yields [22].

Due to the synthetic relevance of alkynyl selenides several methodologies for their synthesis have been developed. Among them are reactions between lithium or sodium acetylides and electrophilic selenium reactants [23]. The use of hypervalent iodine(III) species [24] or alkynyl bromides with RSeLi [25] as nucleophilic selenium species or the reaction of alkynyl bromides under Cu catalysis [26–29], from terminal alkynes in the presence of bases and Cu [30–33], Fe [34–35] or In [36] catalysis, or with *t-*BuOK without of transition metal [37] have been reported. In general, these methodologies require selenyl halides or diselenides as starting materials, which limit their application. Furthermore, in most cases these procedures involve the use of reducing agents, such as elemental Mg or Zn, transition-metal catalysis, inert atmosphere, controlled temperatures, and long reaction times.

**Scheme 1:**
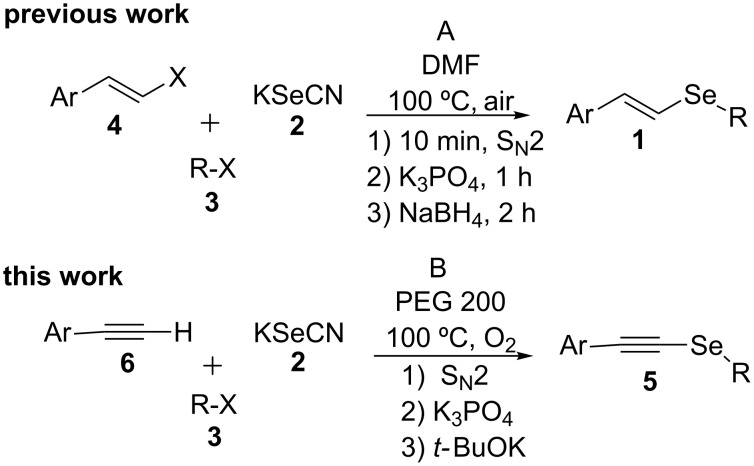
One-pot synthesis of vinyl and alkynyl selenides.

Recently, we have reported the synthesis of vinyl selenides **1** by a three-step one-pot procedure using KSeCN (**2**), alkyl (**3**) and styryl halides (**4**) with good yields (Scheme 1, A) [38]. This reaction proceeds through an initial nucleophilic substitution reaction between the alkyl halide and KSeCN to afford the corresponding alkyl selenocyanate (RSeCN). The treatment of the latter with K_3_PO_4_ generates the alkyl selenolate anion (RSe^−^) which reacts with another RSeCN molecule to give the corresponding diselenide R_2_Se_2_. Reduction of the latter diselenide by treatment with NaBH_4_ affords an alkyl selenolate anion, which reacts with styryl halide to give the desired styryl selenide **1**. During optimization of the reaction conditions to **1** and in the presence of only *t-*BuOK, the corresponding alkynyl selenide **5** was obtained together with dialkyl selenides R_2_Se and R_2_Se_2_. In view of the important synthetic application of alkynyl selenides, we envisioned the possibility of obtaining alkynyl analogues based on our methodology developed for the synthesis of vinyl selenides.

We herein report the optimized reaction conditions for the one-pot and three-step synthesis of alkynyl selenides **5** using KSeCN as selenium source in PEG 200 as the solvent under an oxygen atmosphere. The reaction does not require diselenides or selenyl halides as starting electrophilic reagents, thus offering important advantages over the methods currently reported in the literature (Scheme 1, B).

## Results and Discussion

### Optimization of conditions

**Scheme 2:**
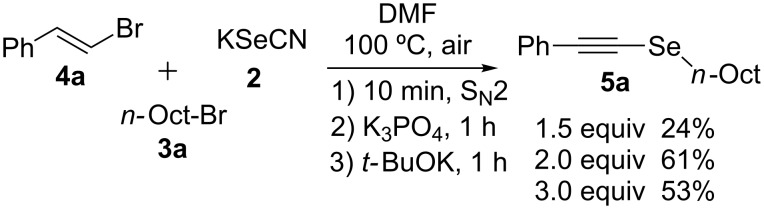
Effect of *t-*BuOK on the formation of *n*-octyl alkynyl selenide **5a**.

During a base screening for the synthesis of alkyl styryl selenides (**1**) [38], we observed that the addition of K_3_PO_4_ to RSeCN, obtained in situ by reaction of RX with KSeCN, afforded exclusively the corresponding R_2_Se_2_. Based on this result we started seeking the best conditions to afford alkynyl selenide **5a** using styryl bromide (**4a**), **2** and *n*-octyl bromide (**3a**). Thus, we applied the optimized conditions for the synthesis of **1** (Scheme 1, A), replacing the reducing agent by *t-*BuOK in the third step. When 1.5 equiv of *t-*BuOK with regard to **4a** were employed in the reaction, alkyne **5a** was obtained in 24%, while the use of 2.0 and 3.0 equiv increased the yield to 61% and 53%, respectively (Scheme 2). Using more than two equiv of base resulted in a lower yield of alkyne **5a** and the formation of styryl selenide **1a** could be detected.

Afterward we performed some control experiments. Thus, the reaction of styryl bromide (**4a**) with 1 equiv of *t-*BuOK afforded 64% yield of phenylacetylene (**6a**), while the elimination reaction was quantitative with 2 equiv of the base. These results suggested that **6a** is a key intermediate in the reaction. Furthermore, the fact that in the presence of 1 equiv of base the elimination product was obtained in approximately 50% yield implies that the deprotonation of **6a** is faster than the elimination from **4a**.

Since **6a** is an intermediate compound of the reaction, we optimized the reaction conditions using terminal alkyne **6a** as the substrate instead of styryl bromide (**4a**), following a three-step one-pot procedure (see Scheme 2). First, we explored the reactivity of KSeCN (**2**), *n*-octyl bromide (**3a**) and acetylene **6a** in DMF as solvent under different atmospheric conditions (Table 1, entries 1–3). Conducting the reaction under an inert atmosphere, compound **5a** was obtained with a yield of 38%. A slightly higher performance was observed when the reaction was carried out under an air atmosphere (41%) and a remarkably improved yield of 71% was obtained under an oxygen atmosphere with continuous bubbling. These results show that the presence of oxygen in the reaction medium contributes to the formation of the expected product.

**Table 1:** Solvent screening for the one-pot synthesis of selenide **5a**.^a^

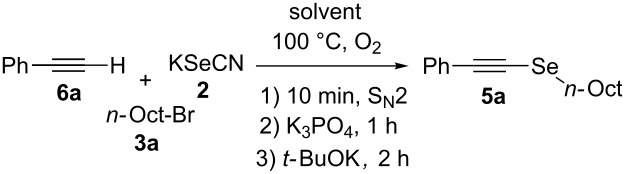		

entry	solvent	yield % **5a**^b^

1	DMF	71
2^c^	DMF	38
3^d^	DMF	41
4	MeCN	47
5	NMP	9
6	DMSO	10
7	dioxane	<5
8	toluene	18
9	ethanol	43
10	isopropanol	65
11	water	nd
12	PEG 300	63
**13**	**PEG 200**	**71**

^a^The reaction was performed by using: 0.25 mmol of **6a**, 1.35 equiv of **2**, **3a** and K_3_PO_4_ and 1.1 equiv of *t*-BuOK in 2 mL of solvent under O_2_ atmosphere, unless otherwise indicated. ^b^Quantified by GC with internal standard. nd: not detected. ^c^Under N_2_. ^d^Open to air.

Next, we performed a screening of solvents under oxygen atmosphere (Table 1). Best yields were obtained with DMF, isopropanol or polyethylene glycol (PEG) as the solvents (Table 1, entries 1, 10, 12 and 13), whereas only moderate yields below 50% were obtained when the reaction was conducted in acetonitrile or ethanol (Table 1, entries 4 and 9). Poor yields below 20% were observed by using *N*-methylpyrrolidone (NMP), dimethyl sulfoxide (DMSO), dioxane or toluene (Table 1, entries 5–8). Finally, product **5a** was not detectable at all when performing the reaction in water. As similar yields were found with DMF and PEG 200, we chose PEG 200 as the solvent for subsequent reactions, which is a non-toxic and inexpensive biodegradable material also used as a sustainable and alternative solvent due to its reusability [39].

**Scheme 3:**
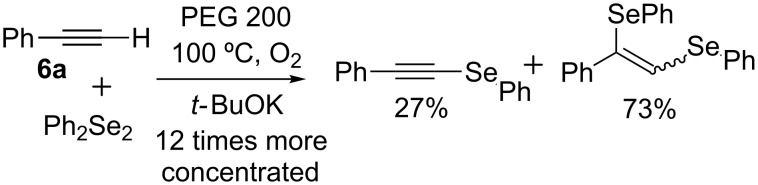
Effect of reactants concentration on alkynyl selenide formation.

Table 2 summarizes the results for the screening and concentration of the base used. Formation of **5a** increases as the volume of solvent increases from 1 mL to 3 mL, with yields of 61%, 71% and 84%, respectively (Table 2, entries 1–3); no considerable change (80%) was observed in 4 mL of PEG 200 (Table 2, entry 4). To understand this trend, the reaction between **6a** and Ph_2_Se_2_ was performed, in which the concentration of reactants was twelve times higher than that under standard conditions, Scheme 3, observing the formation of phenyl (2-phenylethynyl)selane [24] and the addition product 1-phenyl-2-(phenylselanyl)vinylselanylbenzene [40] (27% and 73% yields of relative area by GC, respectively). This result shows that under the conditions used, the Ph_2_Se_2_ addition reaction to the triple bond competes with Se-alkynylation. This side reaction is less efficient when the concentration decreases, favoring the reaction of interest when the amount of solvent increases.

**Table 2:** Concentration effects and base screening for the one-pot synthesis of selenide **5a**.^a^

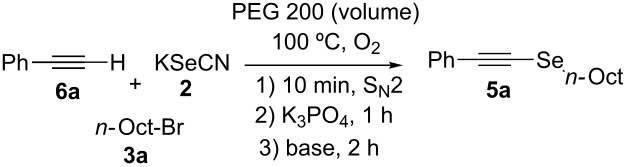			

entry	base	PEG 200 (mL)	yield % **5a**^b^

1	*t-*BuOK	1	61
2	*t-*BuOK	2	71
3	*t-*BuOK	3	84
4	*t-*BuOK	4	80
5	KOH	3	37
6	NaOMe	3	55
7^c^	CuI/Et_3_N	3	31
**8^d^**	***t-*BuOK**	**3**	**93 (78)^e^**
9^d,f^	*t-*BuOK	3	(56)^e,g^
10^f,h^	*t-*BuOK	3	26^i^

^a^The reaction was performed by using: 0.25 mmol of **6a**, 1.35 equiv of **2**, **3a** and K_3_PO_4_ and 1.1 equiv of *t*-BuOK in PEG 200 as solvent under O_2_ atmosphere, unless otherwise indicated. ^b^Quantified by GC with internal standard. ^c^CuI (10 mol %), 1.1 equiv of Et_3_N at 100 °C for 24 h under N_2_ atmosphere. ^d^0.25 mmol of **6a**, 1.0 equiv of **2**, **3a** and K_3_PO_4_, and 2.0 equiv of *t*-BuOK; **6a** and *t*-BuOK were added in the last step. ^e^Isolated yield. ^f^Without addition of K_3_PO_4_. ^g^Together with R_2_Se and R_2_Se_2_ (35% with a ratio of 1:6). ^h^*n*-Octyl selenocyanate, prepared by reaction of **3a** (0.25 mmol) and **2** (0.25 mmol) in 1 mL of PEG 200, was dropped to a solution of alkynyl anion, obtained by reaction of **6a** (0.25 mmol) and *t*-BuOK (0.25 mmol) in 2 mL of PEG 200 under N_2_ atmosphere, and heated at 100 °C for 2 h. ^i^Together with a mixture of R_2_Se and R_2_Se_2_ (74% with a ratio of 1:3).

Subsequently, a set of bases able to deprotonate the alkyne in the last step was tested. By using KOH or NaOMe, **5a** was generated in 37% and 55% yield, respectively (Table 2 entries 5 and 6). The CuI/Et_3_N system, a commonly used combination to activate alkynes in Sonogashira reactions, was also tested, giving 31% yield of **5a** (Table 2, entry 7). However, *t*-BuOK afforded the best results and therefore selected for the subsequent reactions. Next, the ratio of reactants was further investigated in order to obtain the best possible product yield. Thus, by using **6a**/**2**/**3a**/K_3_PO_4_/*t*-BuOK at a 1:1:1:1:2 ratio, respectively, and by adding **6a** with *t-*BuOK in the last step, the yield of selenide **5a** was 93% quantified by GC and 78% isolated yield (Table 2, entry 8). As a control experiment, the reaction was repeated under optimized conditions but without the addition of K_3_PO_4_ (Table 2, entry 9). As previously observed [38], the reaction occurred with the formation of di-*n*-octyl selenide and di-*n*-octyl diselenide as byproducts, decreasing the yield of **5a** to 56% and hampering its isolation. Similar results were obtained when adding *n*-octyl selenocyanate to a solution of alkynyl anion under N_2_ atmosphere followed by heating at 100 °C for 2 h (Table 2, entry 10).

With the optimized reaction conditions at hand, we explored the scope and limitation of this methodology (Scheme 1B) for the synthesis of alkyl alkynyl selenides **5** from various substituted terminal arylacetylenes **6**, synthesized according to known procedures [41–42], and different alkyl halides **3**.

Table 3 comprises the results obtained from the reaction of phenylacetylene (**6a**) with different alkyl halides **3**. The reaction of primary alkyl bromides afforded the corresponding alkynyl selenide **5** in moderate to good yields. It is worth mentioning, that replacing *n*-octyl bromide by the corresponding tosylated compound, the yield of **5a** decreased from 78% to 67% (Table 3, entries 1 and 2). A comparable reactivity was found for the reactions with methyl iodide and *n*-butyl bromide, affording **5b** and **5c** in 77% and 79% yields, respectively (Table 3, entries 3 and 4). However, with 6-bromohex-1-ene the yield of compound **5d** dropped to 41% (Table 3, entry 5) and 2-methyltetrahydro-2*H*-selenopyran was detected by GC–MS. This indicates a possible competitive intramolecular addition reaction between the selenium atom-centered radical and the alkenyl moiety.

**Table 3:** One-pot synthesis of alkynyl selenides **5**.^a^

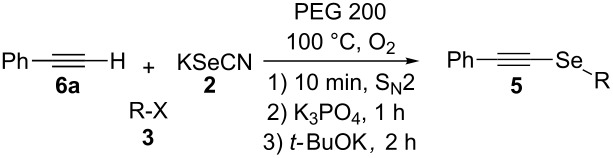			

entry	R–X	yield % **5**^b^	

1	*n*-OctBr	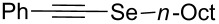 **5a**	78
2	*n*-OctOTs	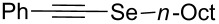 **5a**	67
3	MeI	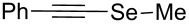 **5b**	77
4	*n*-BuBr	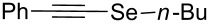 **5c**	79
5	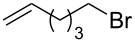	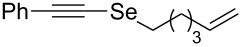 **5d**	41
6	*c*-HexCH_2_Br	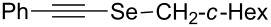 **5e**	56
7	*c*-HexBr	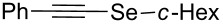 **5f**	nd
8	*t-*BuBr	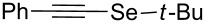 **5g**	nd
9	PhCH_2_Br	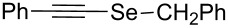 **5h**	nd

^a^The reaction was performed by using: 0.25 mmol of **6a**, 1.0 equiv of **2**, **3** and K_3_PO_4_ and 2.0 equiv of *t*-BuOK, in 3 mL PEG 200 as solvent under an O_2_ atmosphere; **6a** and *t*-BuOK were added in the last step. ^b^Isolated yield, nd: not detected.

The reaction with (bromomethyl)cyclohexane afforded **5e** in 56% yield (Table 3, entry 6). The difference in reactivity relative to **5a** is probably due to steric hindrance caused by the cyclohexyl ring to the alkynyl anion attack. The basic reaction conditions were responsible for promoting elimination reactions (E_2_) when secondary and tertiary alkyl halides were used, preventing formation of **5f** and **5g** products, and this is a limitation of the procedure reported herein (Table 3, entries 7 and 8). By employing a benzylic halide such as benzyl bromide, the expected product **5h** (Table 3, entry 9) was not detected. Again, the basic environment and moderate acidity of the methylene protons in benzyl selenocyanate (**7h**) or dibenzyl diselenide (**8h**) may afford intermediates prone to decomposition, directly affecting formation of **5h**.

Furthermore, the electronic and steric effects offered by different substituents on the aryl moieties attached to the acetylenic group were also studied (Table 4). Thus, the *p*-methyl-substituted derivative **6b** afforded the selenide **5i** in comparable yields as with the unsubstituted alkyne **6a** (Table 4, entries 1 and 2). The presence of a strong electron donor such as a methoxy group (compound **6c**), decreased the yield of product **5j** to 52%. This is ascribed to a lower acidity of the acetylenic proton in **6c** in relation to the unsubstituted alkyne **6a** (Table 4, entry 3). For phenylethynyl derivatives substituted in *para* or *ortho* position by halides, the yields of the corresponding products **5k** and **5l** were 64% and 61%, respectively (Table 4, entries 4 and 5). The *ortho*-substituent showed no remarkable steric hindrance since the reactive center was located relatively far from this group.

**Table 4:** One-pot synthesis of alkynyl selenide **5**.^a^

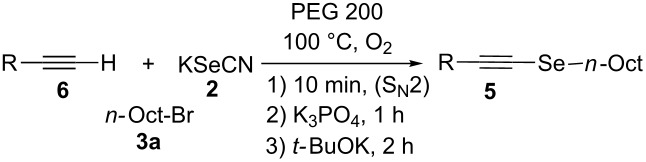			

entry	R, **6**	product **5**	yield %^b^

1	Ph **6a**	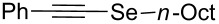 **5a**	78
2	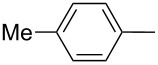 **6b**	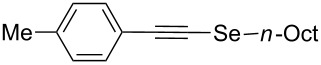 **5i**	81
3	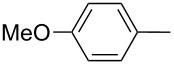 **6c**	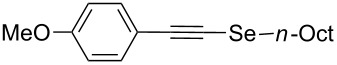 **5j**	52
4	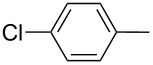 **6d**	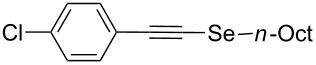 **5k**	64
5	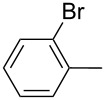 **6e**	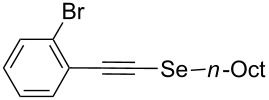 **5l**	61
6	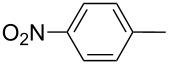 **6f**	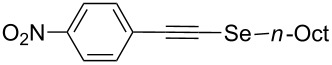 **5m**	nd
7	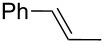 **6g**	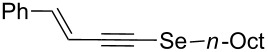 **5n**	53
8	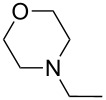 **6h**	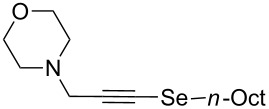 **5o**	nd
9^c^	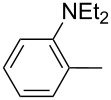 **6i**	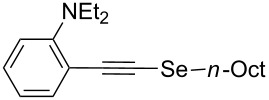 **5p**	<5^d^
10^c^	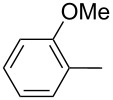 **6j**	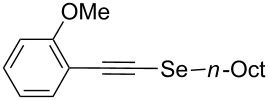 **5q**	51

^a^The reaction was performed by using: 0.25 mmol of **6**, 1.0 equiv of **2**, **3a** and K_3_PO_4_ and 2.0 equiv of *t*-BuOK, in 3 mL of PEG 200 as solvent under an O_2_ atmosphere; **6** and *t*-BuOK were added in the last step. ^b^Isolated yield, nd: not detected. ^c^3.0 equiv of *t*-BuOK and stirring at 100 °C for 6 h were used in the last step. ^d^The main product was 1-ethyl-2-(octylselanyl)-1*H*-indole (**9**) in 62% isolated yield.

On the other hand, in the presence of a strong electron-withdrawing substituent such as a nitro group, the corresponding product **5m** was not detected (Table 4, entry 6). This result indicates that although the acidity of acetylenic proton in **6f** is higher than that for the unsubstituted one **6a**, the resulting anion is highly stabilized by this group, thus reducing its nucleophilic character and being unreactive against di(*n*-octyl) diselenide (**8a**). Alkyne **6g**, having a styryl group afforded compound **5n** in 53% yield (as a mixture of *E* and *Z* isomers in a 5:1 ratio, respectively; Table 4, entry 7) and the aliphatic terminal alkyne **6h** failed to react under the selected conditions (Table 4, entry 8).

**Scheme 4:**
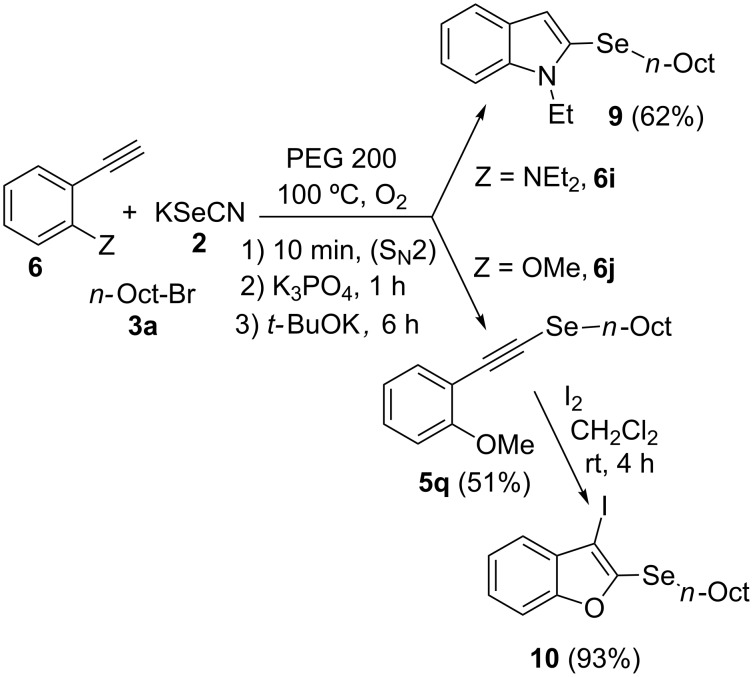
Synthesis of *N*-ethyl-2-(*n*-octylselanyl)-1*H-*indole (**9**) and 3-iodo-2-(*n*-octylselanyl)benzofuran (**10**).

In order to apply the present methodology to the one-pot synthesis of heterocycles derived from indole and benzofuran, convenient *ortho*-substituted phenylacetylenes were also tested. To our delight, the *o*-diethylamino-substituted derivative **6i** afforded *N*-ethyl-2-(*n*-octylselanyl)-1*H*-indole (**9**) in 62% isolated yield (Scheme 4). The primary selenyl-substituted product **5p** was only detected in traces by GC–MS suggesting that this product cyclized in the reaction medium to afford indole **9** (Table 4, entry 9). On the other hand, when *o*-MeO-substituted phenylacetylene **6j** was employed the corresponding selenyl-substituted product **5q** was isolated in 51% yield (Table 4, entry 10). The subsequent electrophilic addition of I_2_ to selenide **5q** afforded 3-iodo-2-(*n*-octylselanyl)benzofuran (**10**) in excellent yield (93% isolated yield, Scheme 4).

Finally, we compared our method with syntheses reported in the literature that mainly rely on commercially available selenium compounds such as *n*-Bu_2_Se_2_, Me_2_Se_2_ or Ph_2_Se_2_. Our procedure does not require transition-metal catalysts, it uses PEG 200 as solvent within shorter or comparable reaction times and it has the advantage of in situ generating R_2_Se_2_ from commercially available and cheaper salts, that are easier to handle and have a much less unpleasant odor than the aliphatic diselenides (R_2_Se_2_). Thus the global 79% yield of **5c** obtained by our method, involves the generation of R_2_Se_2_ over two steps followed by reaction with phenylacetylene. The other methods reported comparable or smaller isolated yields of **5c** (Table 5).

**Table 5:** Comparison of the present method with other reported procedures for the synthesis of **5c** from **6a** and *n*-Bu_2_Se_2_.

entry	conditions	yield % **5c**^a^	reference

1	NpsFe_3_O_4_ (10 mol %), K_2_CO_3_, DMF, 80 °C, 14 h	51	[34]
2	NpsCuO (10 mol %), K_2_CO_3_, DMSO, 80 °C, 14 h	79	[32]
3	InCl_3_ (10 mol %), Cs_2_CO_3_, DMSO, 80 °C, 12 h	64	[36]
4^b^	CuI (1 equiv), HMPA, N_2_, rt, 2 h	70	[26]
5^b^	BuSeLi, THF, N_2_, 0 °C, 1 h	60	[25]
6^c^	K_3_PO_4_, *t-*BuOK, PEG 200, O_2_, 100 °C, 3 h 10 min	79	this work

^a^Isolated yield. ^b^The reaction was performed by using 1-bromo-2-phenylacetylene as starting material. ^c^The reaction was performed by using KSeCN (1.0 equiv) and *n-*BuBr (1.0 equiv) for the in situ generation of *n*-Bu_2_Se_2_.

### Proposed mechanism

**Scheme 5:**
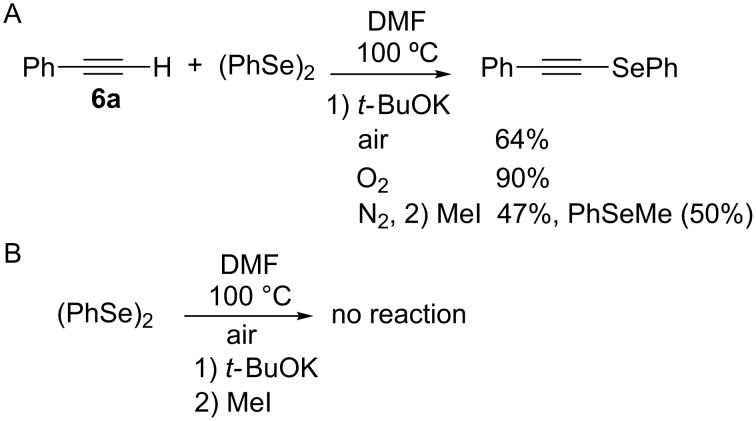
Control reactions and mechanistic study.

In order to assess the mechanism of the three-step one-pot synthesis of **5**, the reaction between the commercial reagents **6a** and 0.5 equiv of diphenyl diselenide (as analogue to the dialkyl diselenides **8** proposed as intermediates) was carried out in the presence of 1 equiv *t-*BuOK at 100 °C for 1 h under air atmosphere. As a result, phenyl(2-phenylethynyl)selane [34] was isolated in 64% yield. Repeating the reaction under oxygen the conversion to the alkynyl selenide increased to 90% (Scheme 5A). After work-up a positive test with I_2_/starch indicator of the aqueous layer confirmed the presence of H_2_O_2_ [43] resulting from the superoxide radical anion (O_2_^·−^) generated during reaction under oxygen atmosphere. It is also possible that the superoxide radical anion oxidizes the alkyl selenide anion (**11**) to the corresponding alkyl selenide radical and the formed O_2_^2−^ affords H_2_O_2_ after work-up. On the other hand, when the same reaction was performed under a nitrogen atmosphere and after one hour quenched with MeI, alkynyl selenide was formed in 47% yield together with methyl(phenyl)selane in 50% yield, determined as relative areas by GC. The latter result confirms that under inert gas atmosphere the phenyl selenide anion remains unoxidized and can be trapped by methylation. Furthermore, under the current reaction conditions no reduction of Se–Se bonds by the used base *t*-BuOK as proposed in the literature takes place (Scheme 5B) [44].

**Scheme 6:**
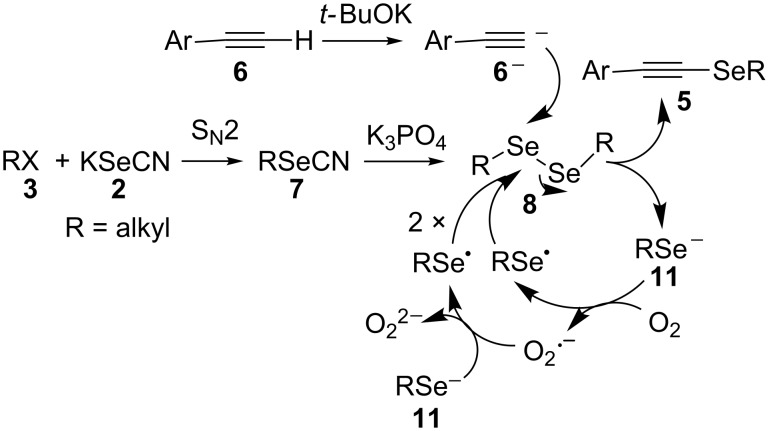
Proposed mechanism for the formation of selenides **5**.

To account for the results obtained we propose the following mechanism for the formation of alkynyl selenides (**5**) (Scheme 6). In the first step a nucleophilic substitution reaction between the alkyl halide **3** with KSeCN (**2**) yields alkyl selenocyanate **7**, which, after addition of K_3_PO_4_, quantitatively affords dialkyl diselenide **8** [38]. Subsequently, the arylacetylene **6** and *t*-BuOK are added with concomitant generation of the corresponding arylacetylene anion. The anion of **6**, once formed, attacks the diselenide intermediate **8**, leading to the expected product **5** and an alkyl selenolate anion (**11**). Given the fact, that the reaction occurs under air atmosphere, the presence of oxygen promotes the oxidation of anion **11** to **8** [45], continuing the cycle until complete consumption of **11**.

**Scheme 7:**
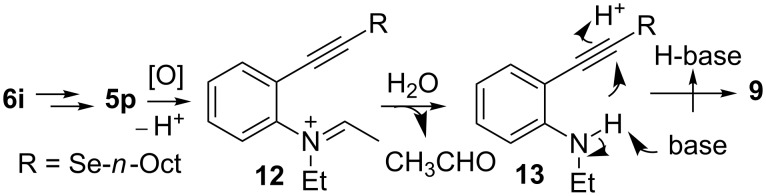
Proposed mechanism for the formation of indole **9**.

Due to the aerobic conditions and the presence of oxidative species such as O_2_^·−^, O_2_^2−^ or H_2_O_2_, the oxidative retro-alkylation is favored [46–49]. Therefore, we can explain the formation of indole **9** by oxidation of **5p**, generated in situ as described above, to afford the iminium intermediate **12**. Hydrolysis of the latter liberates the secondary amine **13** (Scheme 7). This intermediate was detected by GC–MS in traces. Finally, the presence of base in excess and the correct arrangement and electronic properties of both the amine and alkynyl groups promote a spontaneous cyclization reaction to form indole **9** in good yields [50].

## Conclusion

We have developed a novel one-pot procedure for the synthesis of alkyl alkynyl selenides in moderate to good yields. The reaction does not require selenolate anions as starting materials and proceeds through the in situ formation of diselenides from commercially available potassium selenocyanate. Furthermore, the reaction readily takes place in PEG 200, a sustainable solvent, in short reaction times and under an oxygen atmosphere. The method is also applicable to the synthesis of functionalized heterocycles in good to excellent yields.

## Supporting Information

File 1 Experimental details, characterization data and copies of ^1^H, ^13^C and ^77^Se NMR spectra for products **5a**–**e**, **5i**–**l**, **5n**, **5q**, **9** and **10**.
