# Supplementary material for: Transition-metal-free one-pot synthesis of alkynyl selenides from terminal alkynes under aerobic and sustainable conditions
Source: Beilstein J Org Chem. 2017 May 16;13:910–8. doi: 10.3762/bjoc.13.92 (PMC5480365; doi:10.3762/bjoc.13.92)

Transition-metal-free one-pot synthesis of alkynyl selenides from terminal alkynes under aerobic and sustainable conditions

Adrián A. Heredia and Alicia B. Peñéñory*

Address: INFIQC, Facultad de Ciencias Químicas, Universidad Nacional de Córdoba, Ciudad Universitaria, X5000HUA Córdoba, Argentina

Email: Alicia B. Peñéñory - penenory@fcq.unc.edu.ar

* Corresponding author

Experimental details, characterization data and copies of ^{1}H, ^{13}C and ^{77}Se NMR spectra for products **5a–e**, **5i–l**, **5n**, **5q**, **9** and **10**

Table of Contents

Experimental S3
Materials and methods S3
Experimental procedures and characterization data S3
References S10
^{1}H NMR. *n*-Octyl(phenylethynyl)selane (**5a**) S11
^{13}C NMR. *n*-Octyl(phenylethynyl)selane (**5a**) S12
^{77}Se NMR. *n*-Octyl(phenylethynyl)selane (**5a**) S13
^{1}H NMR. Methyl(phenylethynyl)selane (**5b**) S14
^{13}C NMR. Methyl(phenylethynyl)selane (**5b**) S15
^{77}Se NMR. Methyl(phenylethynyl)selane (**5b**) S16
^{1}H NMR. *n*-Butyl(phenylethynyl)selane (**5c**) S17
^{13}C NMR. *n*-Butyl(phenylethynyl)selane (**5c**) S18
^{77}Se NMR. *n*-Butyl(phenylethynyl)selane (**5c**) S19

^{1}H NMR. Hex-5-en-1-yl(phenylethynyl)selane (**5d**) S20
^{13}C NMR. Hex-5-en-1-yl(phenylethynyl)selane (**5d**) S21
^{77}Se NMR. Hex-5-en-1-yl(phenylethynyl)selane (**5d**) S22
^{1}H NMR.(Cyclohexylmethyl)(phenylethynyl)selane (**5e**) S23
^{13}CNMR.(Cyclohexylmethyl)(phenylethynyl)selane (**5e**) S24
^{77}SeNMR.(Cyclohexylmethyl)(phenylethynyl)selane (**5e**) S25
^{1}H NMR. *n*-Octyl(*p*-tolylethynyl)selane (**5i**) S26
^{13}C NMR. *n*-Octyl(*p*-tolylethynyl)selane (**5i**) S27
^{77}Se NMR. *n*-Octyl(*p*-tolylethynyl)selane (**5i**) S28
^{1}H NMR.((4-Methoxyphenyl)ethynyl)(*n*-octyl)selane (**5j**) S29
^{13}CNMR.((4-Methoxyphenyl)ethynyl)(*n*-octyl)selane (**5j**) S30
^{77}SeNMR.((4-Methoxyphenyl)ethynyl)(*n*-octyl)selane (**5j**) S31
^{1}H NMR. ((4-Chlorophenyl)ethynyl)(*n*-octyl)selane (**5k**) S32
^{13}C NMR. ((4-Chlorophenyl)ethynyl)(*n*-octyl)selane (**5k**) S33
^{77}Se NMR. ((4-Chlorophenyl)ethynyl)(*n*-octyl)selane (**5k**) S34
^{1}H NMR. ((2-Bromophenyl)ethynyl)(*n*-octyl)selane (**5l**) S35
^{13}C NMR. ((2-Bromophenyl)ethynyl)(*n*-octyl)selane (**5l**) S36
^{77}Se NMR. ((2-Bromophenyl)ethynyl)(*n*-octyl)selane (**5l**) S37
^{1}HNMR.(*E*,*Z*)-*n*-Octyl(4-phenylbut-3-en-1-yn-1-yl)selane (**5n**) S38
^{13} C NMR.(*E*,*Z*)-*n*-Octyl(4-phenylbut-3-en-1-yn-1-yl)selane (**5n**) S39
^{77}SeNMR.(*E*,*Z*)-*n*-Octyl(4-phenylbut-3-en-1-yn-1-yl)selane (**5n**) S40
^{1}H NMR. ((2-Methoxyphenyl)ethynyl)(*n*-octyl)selane (**5q**) S41
^{13}C NMR. ((2-Methoxyphenyl)ethynyl)(*n*-octyl)selane (**5q**) S42
^{77}Se NMR. ((2-Methoxyphenyl)ethynyl)(*n*-octyl)selane (**5q**) S43
^{1}H NMR. 1-Ethyl-2-(*n*-octylselanyl)-1*H*-indole (**9**) S44
^{13}C NMR. 1-Ethyl-2-(*n*-octylselanyl)-1*H*-indole (**9**) S45
^{77}Se NMR. 1-Ethyl-2-(*n*-octylselanyl)-1*H*-indole (**9**) S46
^{1}H NMR. 3-Iodo-2-(*n*-octylselanyl)benzofuran (**10**) S47
^{13}C NMR. 3-Iodo-2-(*n*-octylselanyl)benzofuran (**10**) S48
^{77}Se NMR. 3-Iodo-2-(*n*-octylselanyl)benzofuran (**10**) S49

Experimental

Materials and methods

KSeCN, methyl(phenyl)selane, alkyl and benzyl halides were all high-purity commercial samples and used without further purification. Terminal aryl acetylenes were synthesized according to known procedures,[1] from the geminal dibromoalkenes previously obtained by a Wittig-type reaction from the corresponding aldehydes employing CBr_4 and PPh_3.[2] DMF, acetonitrile and DMSO absolute grade were used without further purification and stored over molecular sieves (4 Å). Toluene and dioxane were distilled following standard procedures and stored over molecular sieves (4 Å). PEG 200, PEG 300, isopropanol and ethanol were used without further purification. All reaction products were isolated by radial chromatography (silica gel, pentane) from the reaction mixtures and characterized by ^{1}H, ^{13}C and ^{77}Se NMR spectroscopy and mass spectrometry. ^{1}H, ^{13}C and ^{77}Se NMR spectra were recorded at 400.16, 100.62 and 76.28 MHz, respectively on a Bruker 400 spectrometer, and all spectra were reported in δ (ppm) relative to Me_4Si, with $CDCl_3$ as a solvent. The chemical shifts in ^{77}Se spectra are given in ppm using diphenyl diselenide (PhSeSePh) diluted in $CDCl_3$ as an external standard (δ 463 ppm at 25 °C). Gas chromatographic analyses were performed on an Agilent 5890 with a flame-ionization detector, on a 30 m capillary column of a 0.32 mm × 0.25 μm film thickness, with a 5% phenylpolysiloxane phase. GC–MS analyses were conducted on an Agilent 7890 employing a 30 m × 0.25 mm × 0.25 μm with a 5% phenylpolysiloxane phase column. HRMS spectra were recorded on a micrOTOF II LC mass spectrometer. Ionization was achieved by atmospheric pressure chemical ionization (APCI) and the detection setup in the positive ion mode.

Experimental procedures and characterization data

General experimental procedure for the study of the effect of *t*-BuOK on the formation of *n*-octyl(phenylethynyl)selane (5a), (Scheme 2)

The reactions were carried out in a 10 mL three-necked Schlenk tube, equipped with a magnetic stirrer. The tube was charged with DMF (2.0 mL). KSeCN (**2**, 54.0 mg, 0.375 mmol), *n*-octyl bromide (**3a**, 64.3 mg, 0.335 mmol), β-bromostyrene (**4a**, 45.5 mg, 0.25 mmol) were added and stirred for 10 min at 100 °C. K_3PO_4 (79.9 mg, 0.375 mmol) was then added and the mixture was stirred for 1 h. Finally, 1.5, 2.0 or 3.0 equiv of *t*-BuOK were also added and the mixture was stirred for further 2 h. Then the reaction mixture was cooled to room temperature. Diethyl ether (15 mL) and water

(15 mL) were added and the mixture was stirred. The organic layer was separated and the aqueous layer was extracted with diethyl ether (2 × 15 mL). The combined organic extract was dried over anhydrous Na_2SO_4 and the product was isolated by radial chromatography from the crude reaction mixture.

General experimental procedures for one-pot synthesis of alkynyl selenides (5) (Scheme 1B)

The reactions were carried out in a 10 mL three-necked Schlenk tube, equipped with a magnetic stirrer and a PTFE tube (ID: 1 mm, OD: 2 mm) connected externally to a balloon filled with oxygen. The tube was charged with PEG 200 (3.0 mL), verifying the correct bubbling of oxygen into the solvent. KSeCN (**2**, 0.25 mmol) and alkyl halide (**3**, 1.0 equiv) were added and stirred for 10 min at 100 °C. K_3PO_4 (1.0 equiv) was then added and the mixture was stirred for 1 h. Finally, aryl acetylene (**6**, 1.0 equiv) and *t*-BuOK (2.0 equiv) were also added and the mixture was stirred for further 2 h. Then the reaction mixture was cooled to room temperature. Diethyl ether (15 mL) and water (15 mL) were added and the mixture was stirred. The organic layer was separated and the aqueous layer was extracted with diethyl ether (2 × 15 mL). The combined organic extract was dried over anhydrous Na_2SO_4, and the products were isolated by radial chromatography from the crude reaction mixture. The identity of all the products was confirmed by ^{1}H, ^{13}C and ^{77}Se NMR and EIMS.

***n*-Octyl(phenylethynyl)selane (5a):** Following the general procedure, we used KSeCN (**2**, 36.0 mg, 0.25 mmol) and *n*-octyl bromide (**3a**, 48.0 mg, 0.25 mmol) for 10 min at 100 °C, K_3PO_4 (53.3 mg, 0.25 mmol) for 1 h at 100 °C, and phenylacetylene (**6a**, 25.5 mg, 0.25 mmol) and *t*-BuOK (56.0 mg, 0.50 mmol) for 2 h at 100 °C. Purification was performed by radial chromatography (pentane) affording **5a** as a pale yellow oil (57.2 mg, 78% yield). ^{1}H NMR (400 MHz, $CDCl_3$): δ = 7.45–7.36 (m, 2H), 7.28 (m, 3H), 2.87 (t, *J* = 7.4 Hz, 2H), 1.86 (quint, *J* = 7.3 Hz, 2H), 1.45 (quint, *J* = 7.3 Hz, 2H), 1.32 – 1.25 (m, 8H), 0.88 (t, *J* = 6.8 Hz, 3H). ^{13}C NMR (100 MHz, $CDCl_3$): δ = 131.5, 128.2, 128.0, 123.8, 99.4, 70.6, 31.8, 30.2, 29.7, 29.4, 29.2, 29.0, 22.6, 14.1. ^{77}Se NMR (76 MHz, $CDCl_3$): δ = 159.8. GC-MS (EI) *m/z* 294 (30) $[M]^+$, 182 (76), 102 (32), 89 (32), 71 (37), 57 (67), 43 (100). HRMS (APCI) *m/z* calcd for $C_{16}H_{23}Se$ $[M+H]^+$: 295.09600, found 295.09656.

Methyl(phenylethynyl)selane (5b):[3] Following the general procedure, we used KSeCN (**2**, 36.0 mg, 0.25 mmol) and methyl iodide (**3b**, 35.5 mg, 0.25 mmol) for 10 min at 100 °C, K_3PO_4 (53.3 mg, 0.25 mmol) for 1 h at 100 °C, and phenylacetylene (**6a**, 25.5 mg, 0.25 mmol) and *t*-BuOK (56.0 mg, 0.50 mmol) for 2 h at 100 °C. Purification was performed by radial chromatography

(pentane) affording **5b** as a pale yellow oil (37.6 mg, 77% yield). ^{1}H NMR (400 MHz, $CDCl_3$): δ = 7.44 – 7.39 (m, 2H), 7.32 – 7.27 (m, 3H), 2.38 (s, 3H). ^{13}C NMR (100 MHz, $CDCl_3$): δ = 131.5, 128.2, 128.1, 123.5, 98.3, 71.2, 9.8. ^{77}Se NMR (76 MHz, $CDCl_3$): δ = 76.4. GC-MS (EI) *m/z* 196 (97) $[M]^+$, 181 (98), 115 (42), 89 (100), 63 (20).

***n*-Butyl(phenylethynyl)selane (5c):**[4] Following the general procedure, we used KSeCN (**2**, 36.0 mg, 0.25 mmol) and *n*-butyl bromide (**3c**, 34.3 mg, 0.25 mmol) for 10 min at 100 °C, K_3PO_4 (53.3 mg, 0.25 mmol) for 1 h at 100 °C, and phenylacetylene (**6a**, 25.5 mg, 0.25 mmol) and *t*-BuOK (56.0 mg, 0.50 mmol) for 2 h at 100 °C. Purification was performed by radial chromatography (pentane) affording **5c** as a pale yellow oil (46.8 mg, 79% yield). ^{1}H NMR (400 MHz, $CDCl_3$): δ = 7.44 – 7.38 (m, 2H), 7.32 – 7.27 (m, 3H), 2.89 (t, *J* = 7.4 Hz, 2H), 1.86 (quint, *J* = 7.4 Hz, 2H), 1.49 (sext, *J* = 7.4 Hz, 2H), 0.96 (t, *J* = 7.4 Hz, 3H). ^{13}C NMR (100 MHz, $CDCl_3$): δ = 131.5, 128.2, 128.0, 123.7, 99.4, 70.6, 32.2, 29.3, 22.5, 13.5. ^{77}Se NMR (76 MHz, $CDCl_3$): δ = 159.2. GC-MS (EI) *m/z* 238 (43) $[M]^+$, 182 (100), 102 (64), 89 (59), 57 (22), 41 (34).

Hex-5-en-1-yl(phenylethynyl)selane (5d): Following the general procedure, we used KSeCN (**2**, 36.0 mg, 0.25 mmol) and hex-5-en-1-yl bromide (**3d**, 40.8 mg, 0.25 mmol) for 10 min at 100 °C, K_3PO_4 (53.3 mg, 0.25 mmol) for 1 h at 100 °C, and phenylacetylene (**6a**, 25.5 mg, 0.25 mmol) and *t*-BuOK (56.0 mg, 0.50 mmol) for 2 h at 100 °C. Purification was performed by radial chromatography (pentane) affording **5d** as a pale yellow oil (26.9 mg, 41% yield). ^{1}H NMR (400 MHz, $CDCl_3$): δ = 7.46 – 7.36 (m, 2H), 7.34 – 7.26 (m, 3H), 5.88 – 5.74 (m, 1H), 5.06 – 5.00 (m, 1H), 4.99 – 4.93 (m, 1H), 2.88 (t, *J* = 7.3 Hz, 2H), 2.16 – 2.06 (m, 2H), 1.89 (quint, *J* = 7.4 Hz, 2H), 1.56 (quint, *J* = 7.4 Hz, 2H). ^{13}C NMR (100 MHz, $CDCl_3$): δ = 138.3, 131.5, 128.2, 128.0, 123.7, 114.9, 99.5, 70.4, 33.1, 29.6, 29.4, 28.5. ^{77}Se NMR (76 MHz, $CDCl_3$): δ = 160.5. GC-MS (EI) *m/z* 264 (10) $[M]^+$, 182 (62), 155 (27), 149 (25), 141 (51), 115 (23), 102 (53), 89 (80), 55 (100), 41 (67). HRMS (APCI) *m/z* calcd for $C_{14}H_{17}Se$ $[M+H]^+$: 265.04904, found: 265.04978.

(Cyclohexylmethyl)(phenylethynyl)selane (5e): Following the general procedure, we used KSeCN (**2**, 36.0 mg, 0.25 mmol) and cyclohexylmethyl bromide (**3e**, 44.3 mg, 0.25 mmol) for 10 min at 100 °C, K_3PO_4 (53.3 mg, 0.25 mmol) for 1 h at 100 °C, and phenylacetylene (**6a**, 25.5 mg, 0.25 mmol) and *t*-BuOK (56.0 mg, 0.50 mmol) for 2 h at 100 °C. Purification was performed by radial chromatography (pentane) affording **5e** as a pale yellow oil (38.8 mg, 56% yield). ^{1}H NMR

(400 MHz, $CDCl_3$): δ = 7.46 – 7.36 (m, 2H), 7.29 – 7.28 (m, 3H), 2.81 (d, *J* = 6.8 Hz, 2H), 1.96 – 1.87 (m, 2H), 1.77 – 1.64 (m, 4H), 1.34 – 1.23 (m, 2H), 1.16 (ddt, *J* = 12.6, 7.0, 3.0 Hz, 1H), 1.02 (ddd, *J* = 24.1, 12.3, 3.3 Hz, 2H). ^{13}C NMR (100 MHz, $CDCl_3$): δ = 131.5, 128.2, 127.9, 123.8, 98.7, 71.3, 38.3, 37.8, 33.0, 26.2, 26.0. ^{77}Se NMR (76 MHz, $CDCl_3$): δ = 126.8. GC-MS (EI) *m/z* 278 (18) $[M]^+$, 182 (44), 102 (28), 97 (48), 67 (29), 55 (100), 44 (41), 41 (52). HRMS (APCI) *m/z* calcd for $C_{15}H_{19}Se$ $[M+H]^+$: 279.06469, found: 279.06468.

***n*-Octyl(*p*-tolylethynyl)selane (5i):** Following the general procedure, we used KSeCN (**2**, 36.0 mg, 0.25 mmol) and *n*-octyl bromide (**3a**, 48.0 mg, 0.25 mmol) for 10 min at 100 °C, K_3PO_4 (53.3 mg, 0.25 mmol) for 1 h at 100 °C, and *p*-tolylacetylene (**6b**, 29.0 mg, 0.25 mmol) and *t*-BuOK (56.0 mg, 0.50 mmol) for 2 h at 100 °C. Purification was performed by radial chromatography (pentane) affording **5i** as a pale yellow oil (62.4 mg, 81% yield). ^1H NMR (400 MHz, $CDCl_3$): δ = 7.31 (d, *J* = 8.0 Hz, 2H), 7.09 (d, *J* = 7.9 Hz, 2H), 2.86 (t, *J* = 7.4 Hz, 2H), 2.33 (s, 3H), 1.85 (quint, *J* = 7.4 Hz, 2H), 1.44 (quint, *J* = 7.4 Hz, 2H), 1.31 – 1.27 (m, 8H), 0,88 (t, *J* = 6.7 Hz, 3H). ^{13}C NMR (100 MHz, $CDCl_3$): δ = 138.1, 131.5, 129.0, 120.7, 99.5, 69.5, 31.8, 30.2, 29.7, 29.4, 29.2, 29.0, 22.6, 21.5, 14.1. ^{77}Se NMR (76 MHz, $CDCl_3$): δ = 158.7. GC-MS (EI) *m/z* 308 (37) $[M]^+$, 196 (90), 116 (31), 115 (100), 71 (18), 57 (34), 55 (18), 43 (56), 41 (54). HRMS (APCI) *m/z* calcd for $C_{17}H_{25}Se$ $[M+H]^+$: 309.11166, found: 309.11063.

((4-Methoxyphenyl)ethynyl)(*n*-octyl)selane (5j): Following the general procedure, we used KSeCN (**2**, 36.0 mg, 0.25 mmol) and *n*-octyl bromide (**3a**, 48.0 mg, 0.25 mmol) for 10 min at 100 °C, K_3PO_4 (53.3 mg, 0.25 mmol) for 1 h at 100 °C, and 4-methoxyphenylacetylene (**6c**, 33.0 mg, 0.25 mmol) and *t*-BuOK (56.0 mg, 0.50 mmol) for 2 h at 100 °C. Purification was performed by radial chromatography (pentane) affording **5j** as a pale yellow oil (42.0 mg, 52% yield). ^1H NMR (400 MHz, $CDCl_3$): δ = 7.37 (d, *J* = 8.9 Hz, 2H), 6.82 (d, *J* = 8.9 Hz, 2H), 3.80 (s, 3H), 2.85 (t, *J* = 7.4 Hz, 2H), 1.85 (quint, *J* = 7.4 Hz, 2H), 1.44 (quint, *J* = 7.2 Hz, 2H), 1.33 – 1.25 (m, 8H), 0.88 (t, *J* = 6.8 Hz, 3H). ^{13}C NMR (100 MHz, $CDCl_3$): δ = 159.5, 133.3, 115.9, 113.9, 99.1, 68.4, 55.3, 31.8, 30.1, 29.7, 29.4, 29.2, 29.0, 22.6, 14.1. ^{77}Se NMR (76 MHz, $CDCl_3$): δ = 157.4. GC-MS (EI) *m/z* 324 (28) $[M]^+$, 212 (76), 197 (29), 168 (16), 132 (100), 57 (23), 43 (47), 41 (48). HRMS (APCI) *m/z* calcd for $C_{17}H_{25}OSe$ $[M+H]^+$: 325.10657, found: 325.10515.

((4-Chlorophenyl)ethynyl)(*n*-octyl)selane (5k): Following the general procedure, we used KSeCN (**2**, 36.0 mg, 0.25 mmol) and *n*-octyl bromide (**3a**, 48.0 mg, 0.25 mmol) for 10 min at 100 °C, K_3PO_4 (53.3 mg, 0.25 mmol) for 1 h at 100 °C, and 4-chlorophenylacetylene (**6d**, 34.1 mg, 0.25 mmol) and *t*-BuOK (56.0 mg, 0.50 mmol) for 2 h at 100 °C. Purification was performed by radial chromatography (pentane) affording **5k** as pale yellow oil (52.4 mg, 64% yield). ^{1}H NMR (400 MHz, $CDCl_3$): δ = 7.36 – 7.29 (m, 2H), 7.29 – 7.24 (m, 2H), 2.87 (t, *J* = 7.4 Hz, 2H), 1.85 (quint, *J* = 7.3 Hz, 2H), 1.50 – 1.38 (m, 2H), 1.35 – 1.23 (m, 8H), 0.88 (m, 3H). ^{13}C NMR (100 MHz, $CDCl_3$): δ = 133.9, 132.7, 128.6, 122.2, 98.3, 72.1, 31.8, 30.2, 29.7, 29.3, 29.2, 29.0, 22.6, 14.1. ^{77}Se NMR (76 MHz, $CDCl_3$): δ = 161.6. GC-MS (EI) *m/z* 328 (38) $[M]^+$, 216 (99), 180 (25), 136 (27), 123 (18), 71 (56), 57 (81), 55 (27), 43 (100), 41 (86). HRMS (APCI) *m/z* calcd for $C_{16}H_{22}ClSe$ $[M+H]^+$: 329.05676, found: 329.05640.

((2-Bromophenyl)ethynyl)(*n*-octyl)selane (5l): Following the general procedure, we used KSeCN (**2**, 36.0 mg, 0.25 mmol) and *n*-octyl bromide (**3a**, 48.0 mg, 0.25 mmol) for 10 min at 100 °C, K_3PO_4 (53.3 mg, 0.25 mmol) for 1 h at 100 °C, and 2-bromophenylacetylene (**6e**, 45.2 mg, 0.25 mmol) and *t*-BuOK (56.0 mg, 0.50 mmol) for 2 h at 100 °C. Purification was performed by radial chromatography (pentane) affording **5l** as pale yellow oil (56.8 mg, 61% yield). ^{1}H NMR (400 MHz, $CDCl_3$): δ = 7.55 (dd, *J* = 8.0, 0.9 Hz, 1H), 7.42 (dd, *J* = 7.7, 1.6 Hz, 1H), 7.23 (td, *J* = 7.6, 1.1 Hz, 1H), 7.11 (td, *J* = 7.9, 1.7 Hz, 1H), 2.91 (t, *J* = 7.4 Hz, 2H), 1.92 (quint *J* = 7.4 Hz, 2H), 1.45 (quint, *J* = 7.0 Hz, 2H), 1.37 – 1.22 (m, 8H), 0.87 (t, *J* = 6.8 Hz, 3H). ^{13}C NMR (100 MHz, $CDCl_3$): δ = 132.9, 132.3, 128.9, 126.9, 125.8, 125.0, 98.1, 76.6, 31.8, 30.3, 30.0, 29.4, 29.2, 29.1, 22.7, 14.1. ^{77}Se NMR (76 MHz, $CDCl_3$): δ = 166.8. GC-MS (EI) *m/z* 372 (18) $[M]^+$, 260 (45), 219 (15), 180 (23), 71 (56), 69 (21), 57 (100), 55 (32), 43 (98), 41 (66). HRMS (APCI) *m/z* calcd for $C_{16}H_{22}BrSe$ $[M+H]^+$: 373.00618, found: 373.00400.

(*E*,*Z*)-*n*-Octyl(4-phenylbut-3-en-1-yn-1-yl)selane (5n): Following the general procedure, we used KSeCN (**2**, 36.0 mg, 0.25 mmol) and *n*-octyl bromide (**3a**, 48.0 mg, 0.25 mmol) for 10 min at 100 °C, K_3PO_4 (53.3 mg, 0.25 mmol) for 1 h at 100 °C, and (*E*)-but-1-en-3-yn-1-ylbenzene (**6g**, 32.0 mg, 0.25 mmol) and *t*-BuOK (56.0 mg, 0.50 mmol) for 2 h at 100 °C. Purification was performed by radial chromatography (pentane) providing a mixture of *E* and *Z* isomers of **5n** (5:1 respectively) as a pale yellow oil (42.3 mg, 53% yield). <u>*E* isomer</u>: ^{1}H NMR (400 MHz, $CDCl_3$): δ = 7.44 – 7.19 (m, 5H, overhead), 6.89 (d, *J* = 16.2 Hz, 1H), 6.26 (d, *J* = 16.2 Hz, 1H), 2.84 (d, *J* = 7.4 Hz, 2H, overhead),

1,87-1,80 (m, 2H, overhead), 1.50 – 1.38 (m, 2H, overhead), 1.37 – 1.20 (m, 8H, overhead), 0.96–0.79 (m, 3H, overhead). ^{13}C NMR (100 MHz, $CDCl_3$): δ = 140.5, 128.7, 128.5, 128.3, 126.2, 108.5, 99.0, 73.1, 31.8, 30.2, 29.8, 29.4, 29.2, 29.0, 22.7, 14.1. ^{77}Se NMR (76 MHz, $CDCl_3$): δ = 166.2. *Z* isomer: ^{1}H NMR (400 MHz, $CDCl_3$): δ = 7.44 – 7.19 (m, 5H, overhead), 6.54 (d,*J* = 12.0 Hz, 1H), 5.80 (d, *J* = 12.0 Hz, 1H), 2.87 (d, *J* = 7.4 Hz, 2H, overhead), 1.87-1.80 (m, 2H, overhead), 1.50 – 1.38 (m, 2H, overhead), 1.37 – 1.20 (m, 8H, overhead), 0.96 – 0.79 (m, 3H, overhead). ^{13}C NMR (100 MHz, $CDCl_3$): δ = 137.3, 136.6, 136.3, 128.3, 107.6, 98.7, 77.2, signals from aliphatic carbons show them overhead with respect to *E* isomer (except for 30.4 and 29.5); it was not possible to detected the *ipso* carbon signal due to its low intensity. ^{77}Se NMR (76 MHz, $CDCl_3$): δ = 172.0. GC-MS (EI) *m/z* 320 (16) $[M]^+$, 207 (29), 128 (100), 115 (46), 57 (17), 43 (34), 41 (28). HRMS (APCI) *m/z* calcd for $C_{18}H_{25}Se$ $[M+H]^+$: 321.11166, found: 321.11012.

((2-Methoxyphenyl)ethynyl)(*n*-octyl)selane (5q): Following the general procedure, we used KSeCN (**2**, 36.0 mg, 0.25 mmol) and *n*-octyl bromide (**3a**, 48.0 mg, 0.25 mmol) for 10 min at 100 °C, K_3PO_4 (53.3 mg, 0.25 mmol) for 1 h at 100 °C, and 2-methoxyphenylacetylene (**6j**, 33.0 mg, 0.25 mmol) and *t*-BuOK (84.0 mg, 0.75 mmol) for 6 h at 100 °C. Purification was performed by radial chromatography (pentane) affording **5q** as a pale yellow oil (41.2 mg, 51% yield). ^{1}H NMR (400 MHz, $CDCl_3$) δ 7.38 (dd, *J* = 7.5, 1.7 Hz, 1H), 7.29 – 7.22 (m, 1H), 6.92 – 6.83 (m, 2H), 3.87 (s, 3H), 2.88 (t, *J* = 7.4 Hz, 2H), 1.89 (quint, *J* = 7.4 Hz, 2H), 1.48 – 1.41 (m, 2H), 1.35 – 1.22 (m, 8H), 0.88 (t, *J* = 6.9 Hz, 3H). ^{13}C NMR (100 MHz, $CDCl_3$) δ 160.2, 133.6, 129.5, 120.5, 113.1, 110.7, 95.7, 74.6, 55.9, 32.0, 30.2, 29.9, 29.6, 29.3, 29.2, 22.8, 14.2. ^{77}Se NMR (76 MHz, $CDCl_3$) δ 164.0. GC-MS (EI) *m/z* 324 (34) $[M]^+$, 197 (20), 131 (100), 119 (40), 118 (37), 91 (27), 57 (22), 43 (40), 41 (31). HRMS (APCI) *m/z* calcd for $C_{17}H_{25}OSe$ $[M+H]^+$: 325.10657, found: 325.10593.

1-Ethyl-2-(*n*-octylselanyl)-1*H*-indole (9): Following the general procedure, we used KSeCN(**2**, 36.0 mg, 0.25 mmol) and *n*-octyl bromide (**3a**, 48.0 mg, 0.25 mmol) for 10 min at 100 °C, K_3PO_4 (53.3 mg, 0.25 mmol) for 1 h at 100 °C, and *N*,*N*-diethyl-2-ethynylaniline (**6i**, 43.3 mg, 0.25 mmol) and *t*-BuOK (84.0 mg, 0.75 mmol) for 6 h at 100 °C. Purification was performed by radial chromatography (pentane) affording **9** as a yellow oil (52.1 mg, 62% yield). ^{1}H NMR (400 MHz, $CDCl_3$) δ 7.56 (d, *J* = 7.9 Hz, 1H), 7.32 (d, *J* = 8.1 Hz, 1H), 7.18 (td, *J* = 7.1, 1.0 Hz, 1H), 7.08 (td, *J* = 7.0, 1.1 Hz, 1H), 6.68 (s, 1H), 4.34 (quint, *J* = 7.2 Hz, 2H), 2.79 (t, *J* = 7.4, 2H), 1.67 (quint, *J* = 7.4 Hz, 2H), 1.41 – 1.32 (m, 2H) , 1.35 (t, *J* = 7.2 Hz, 3H), 1.29 - 1.20 (m, 8H), 0.87 (t, *J* = 6.8 Hz, 3H).

^{13}C NMR (100 MHz, $CDCl_3$) δ 137.3, 128.5, 125.5, 121.8, 120.2, 119.6, 110.0, 109.7, 39.8, 31.9, 30.3, 30.3, 29.8, 29.3, 29.2, 22.8, 15.6, 14.2. ^{77}Se NMR (76 MHz, $CDCl_3$) δ 157.2. GC-MS (EI) *m/z* 337 (23) $[M]^+$, 225 (60), 223 (32), 145 (100), 130 (28), 117 (11), 43 (11), 41 (11). HRMS (APCI) *m/z* calcd for $C_{18}H_{28}NSe$ $[M+H]^+$: 338.13821, found: 338.13687.

General experimental procedure for the synthesis of 3-iodo-2-(*n*-octylselanyl)benzofuran (10)

To a solution of 0.2 mmol of **5q** (64.7 mg) and 2 mL of CH_2Cl_2, 1.1 equiv. of I_2 dissolved in 2 mL of CH_2Cl_2 was gradually added. The reaction mixture was stirred at room temperature for 4 h. The excess of I_2 was removed by washing with saturated aqueous $Na_2S_2O_3$. Diethyl ether (15 mL) and water (15 mL) were added and the mixture was stirred. The organic layer was separated and the aqueous layer was extracted with diethyl ether (2 × 15 mL). The combined organic extract was dried over anhydrous Na_2SO_4 and the products were isolated by radial chromatography (pentane) affording **10** as a pale yellow oil (101.2 mg, 93% yield). ^1H NMR (400 MHz, $CDCl_3$) δ 7.45 – 7.37 (m, 1H), 7.35 – 7.22 (m, 3H), 3.04 (t, *J* = 7.4 Hz, 2H), 1.78 – 1.66 (quint, *J* = 7.4 Hz, 2H), 1.42 – 1.36 (m, 2H), 1.32 – 1.15 (m, 8H), 0.85 (t, *J* = 6.9 Hz, 3H). ^{13}C NMR (100 MHz, $CDCl_3$) δ 156.9, 148.2, 131.6, 125.4, 123.6, 121.2, 111.1, 75.5, 31.9, 30.8, 29.6, 29.3, 29.1, 29.1, 22.8, 14.2. ^{77}Se NMR (76 MHz, $CDCl_3$) δ 230.5. GC-MS (EI) *m/z* 436 (80) $[M]^+$, 323 (95), 197 (10), 168 (34), 89 (15), 71 (43), 57 (66), 55 (19), 43 (96), 41 (74). HRMS (APCI) *m/z* calcd for $C_{16}H_{21}INaOSe$ $[M+Na]^+$: 458.96950, found: 458.96898.

General procedure for the reaction between phenylacetylene (6a) and diphenyl diselenide (Scheme 5A)

The reactions were carried out in a 10 mL three-necked Schlenk tube, equipped with a nitrogen or oxygen gas inlet and a magnetic stirrer. The tube was charged with DMF (2.0 mL). Phenylacetylene (**6a**, 25.5 mg, 0.25mmol), diphenyl diselenide (39.2 mg, 0.125 mmol) and *t*-BuOK (28.0 mg, 0.25 mmol) were added and stirred for 2 h at 100 °C. The reaction mixture was cooled to room temperature. The reaction performed under nitrogen atmosphere was quenched with an excess of methyl iodide (63.5 mg, 0.5 mmol) and stirred at room temperature for 1 h. Diethyl ether (15 mL) and water (15 mL) were added and the mixture was stirred. The organic layer was separated and the aqueous layer was extracted with diethyl ether (2 × 15 mL). The combined organic extract was dried over anhydrous Na_2SO_4 and the products were detected by GC–MS and quantified by GC with internal standard.

References

1. a) Michel, P.; Gennet, D.; Rassat, A. *Tetrahedron Lett.* **1999***, 40*, 8575-8578. b) Hijfte, L. V.; Kolb, M.; Witz, P. *Tetrahedron Lett.* **1989***, 30*, 3655-3656.
2. Corey, E. J.; Fuchs, P. L. *Tetrahedron Lett.* **1972***, 13*, 3769-3772.
3. Sharma, A.; Schwab, R. S.; Braga, A. L.; Barcellos, T.; Paixão, M. W. *Tetrahedron Lett.* **2008***, 49*, 5172-5174.
4. Godoi, M.; Liz, D. G.; Ricardo, E. W.; Rocha, M. S. T.; Azeredo, J. B.; Braga, A. L. *Tetrahedron* **2014***, 70*, 3349-3354.

^{1}H NMR. *n*-Octyl(phenylethynyl)selane (5a)

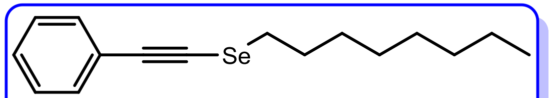

5a

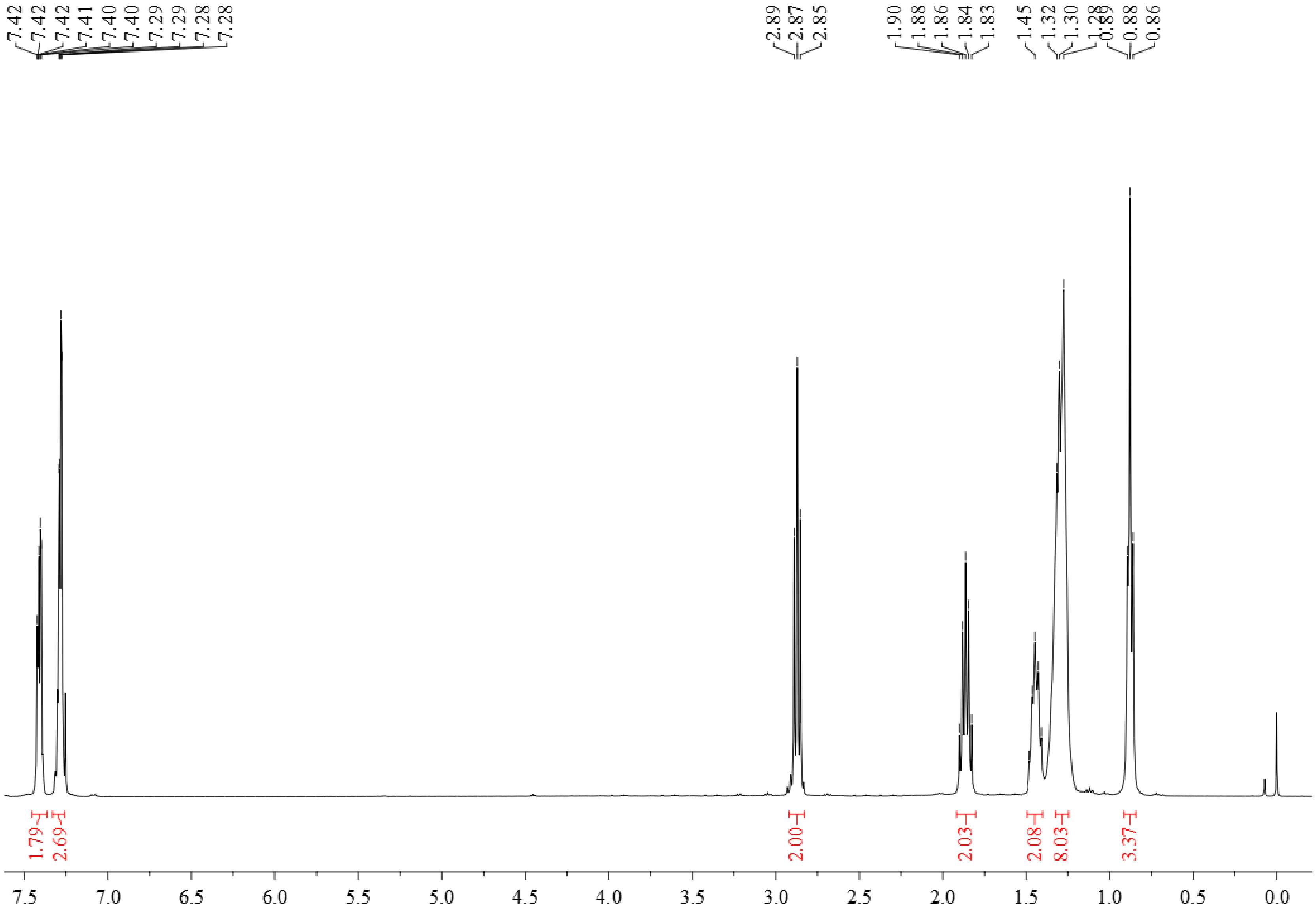

^{13}C NMR. *n*-Octyl(phenylethynyl)selane (5a)

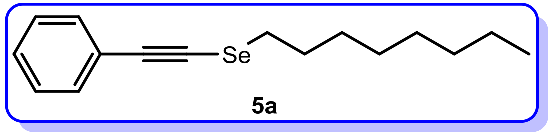

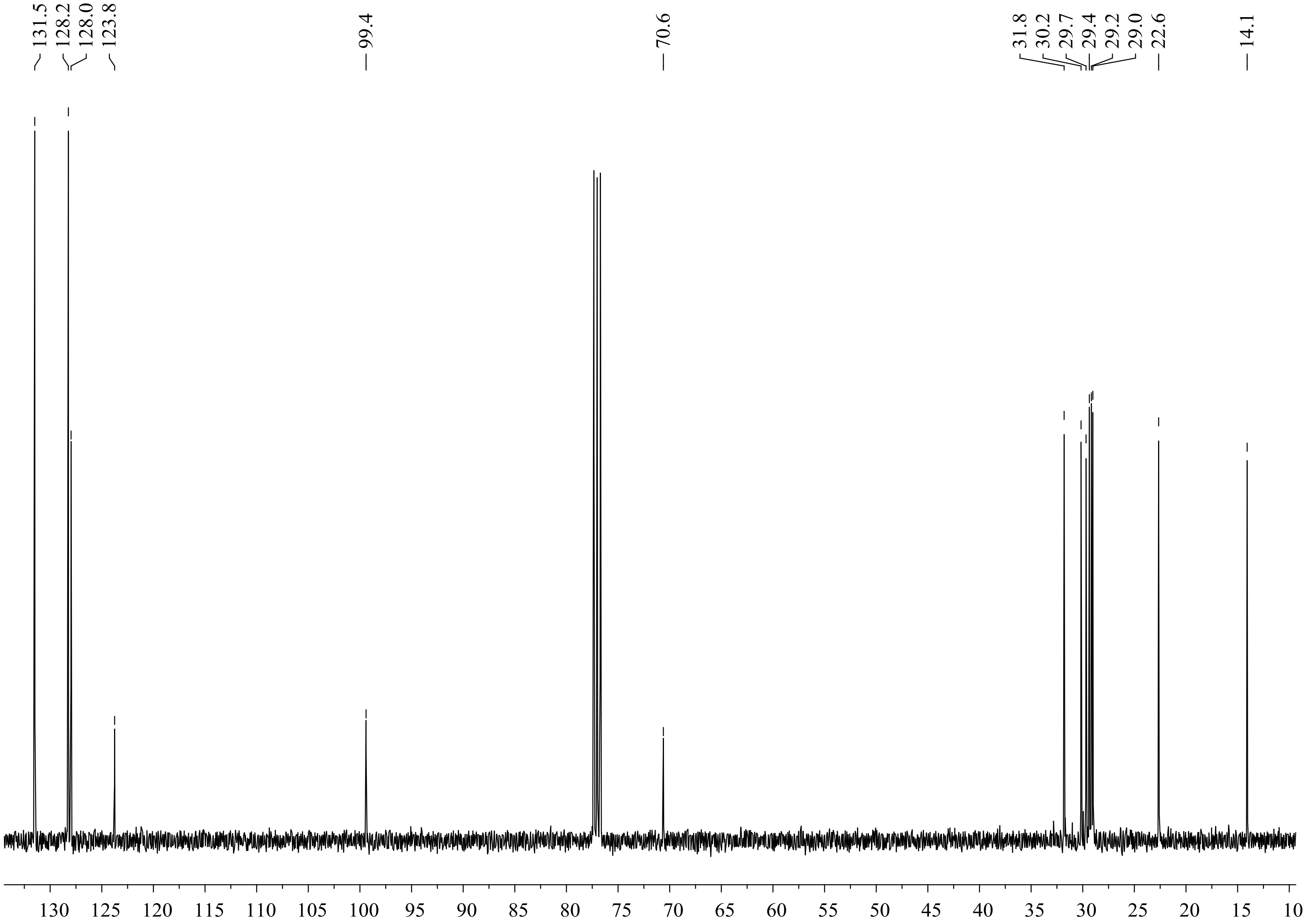

^{77}Se NMR. *n*-Octyl(phenylethynyl)selane (5a)

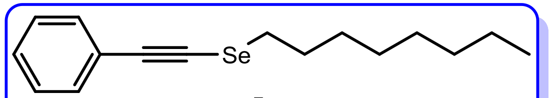

5a

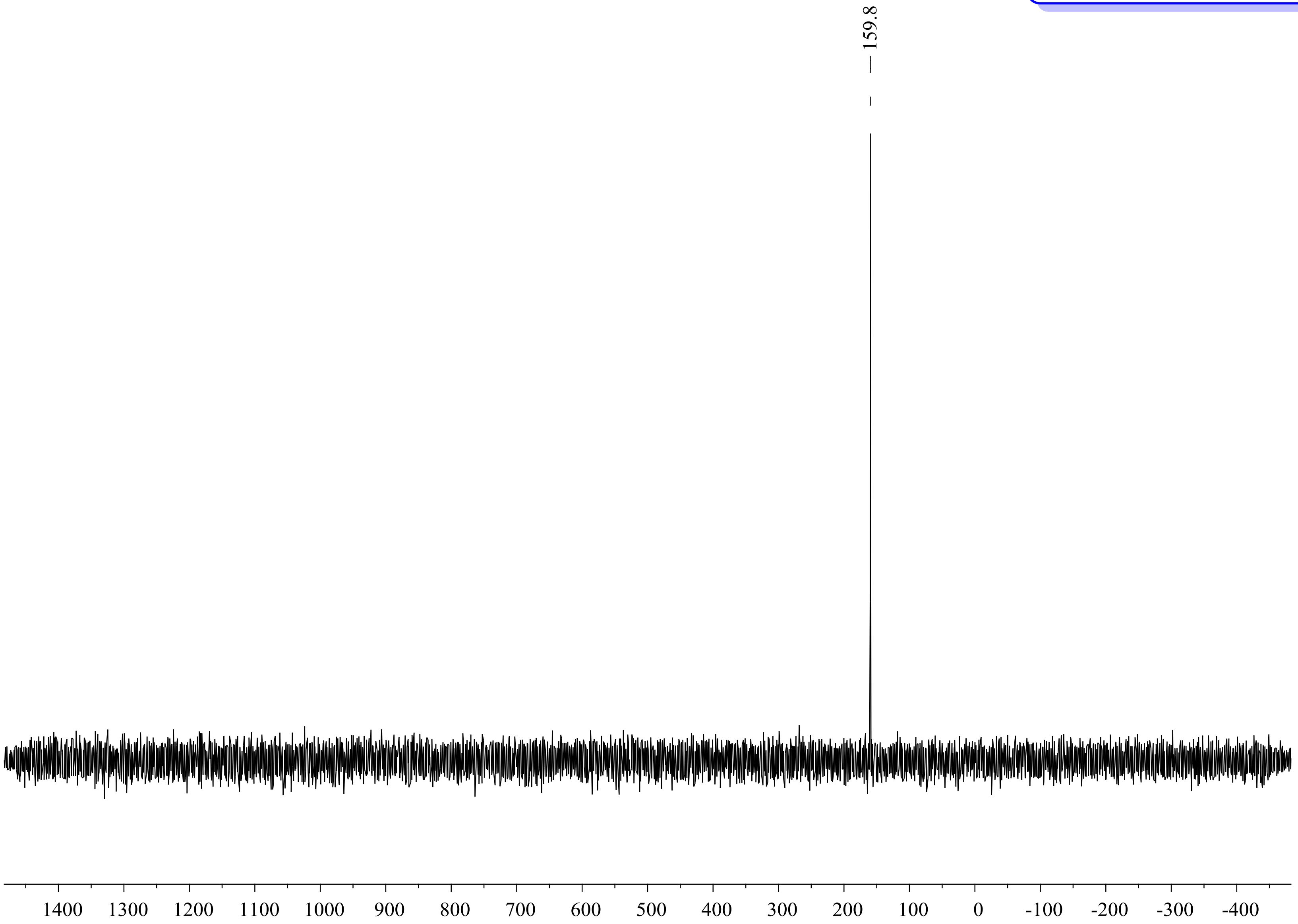

^{1}H NMR. Methyl(phenylethynyl)selane (5b)

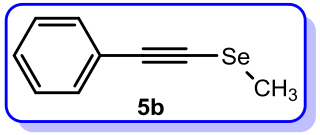

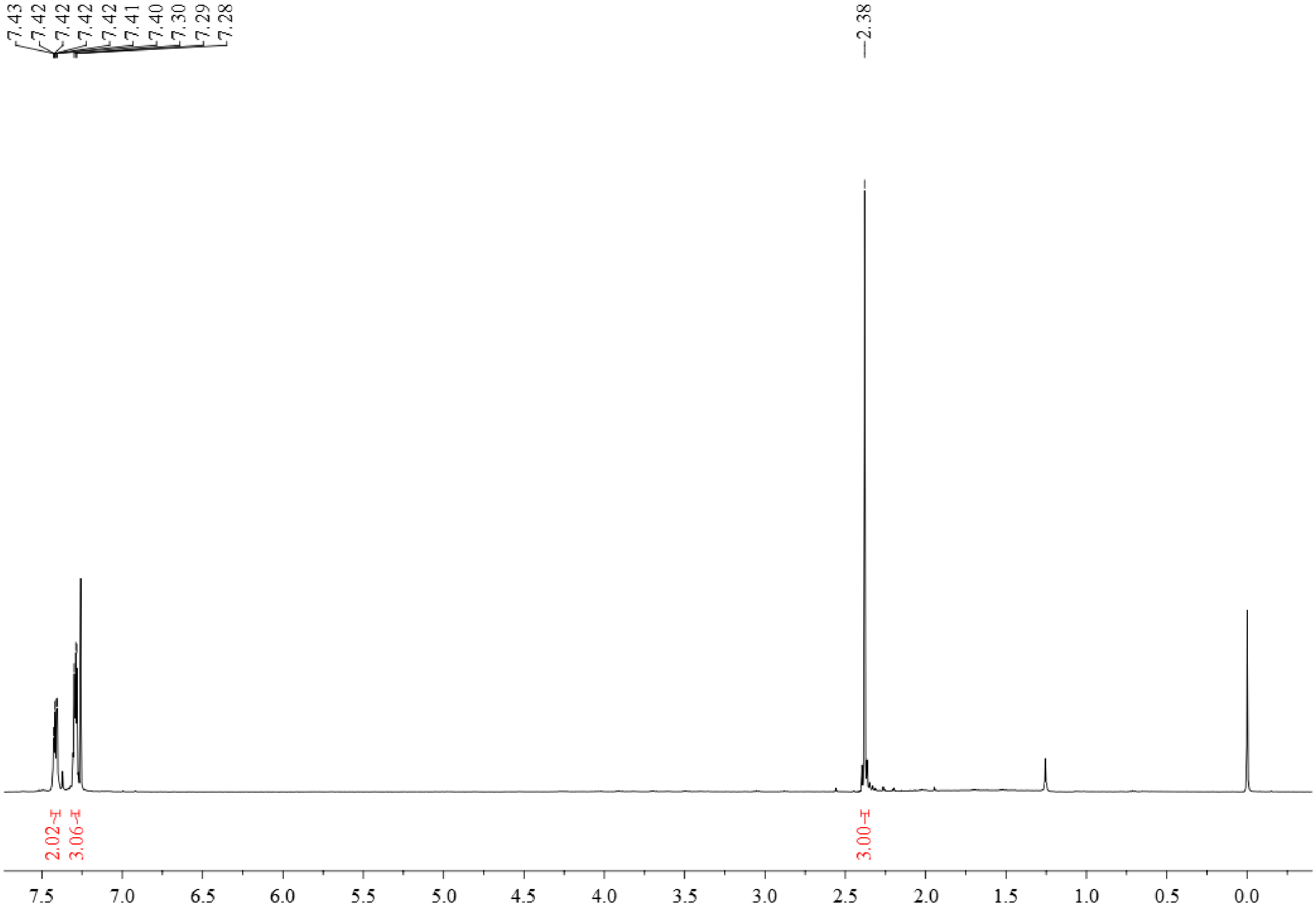

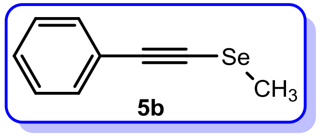

^{13}C NMR. Methyl(phenylethynyl)selane (5b)

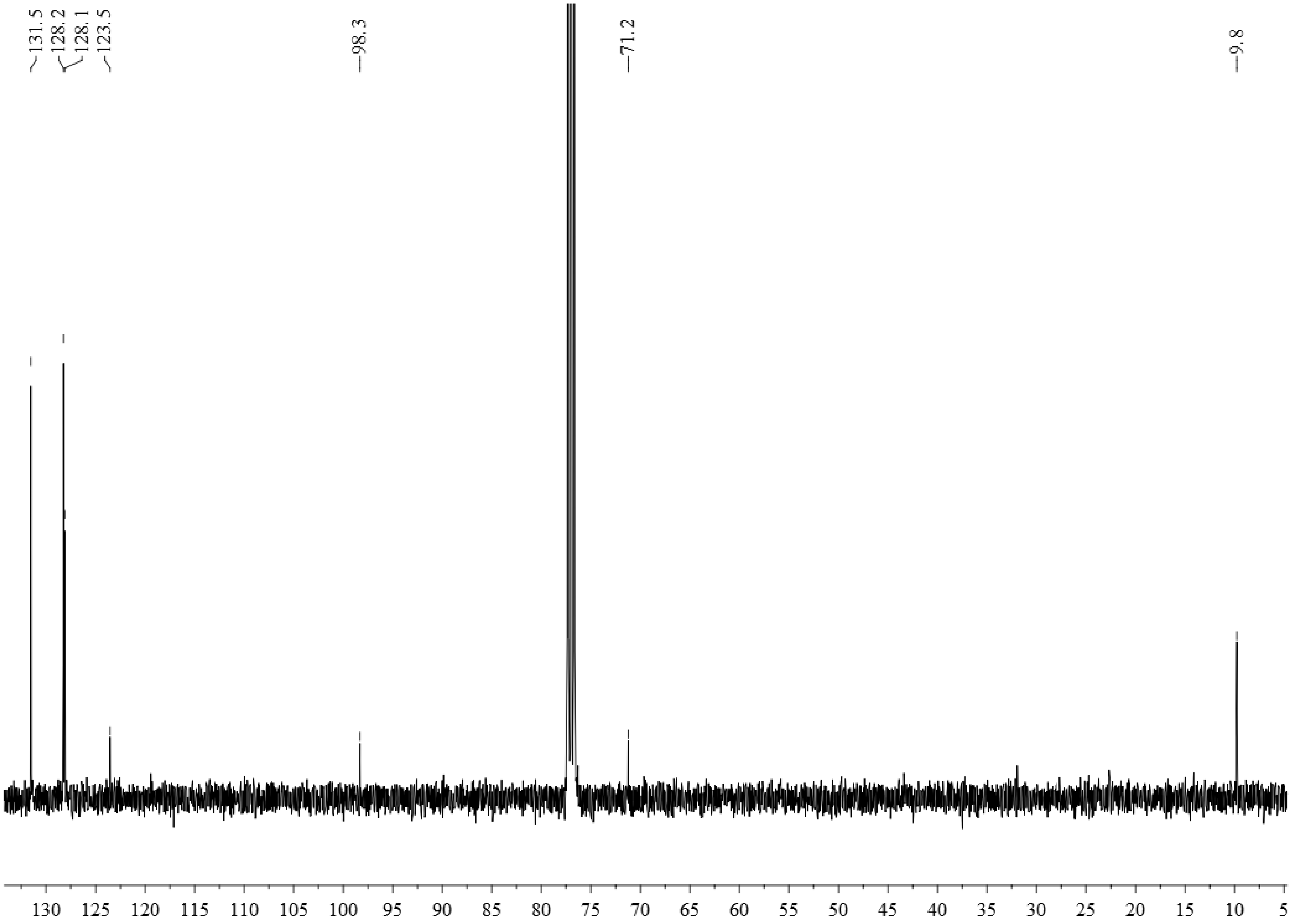

^{77}Se NMR. Methyl(phenylethynyl)selane (5b)

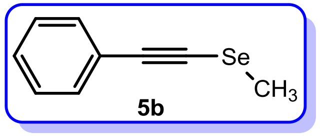

5b

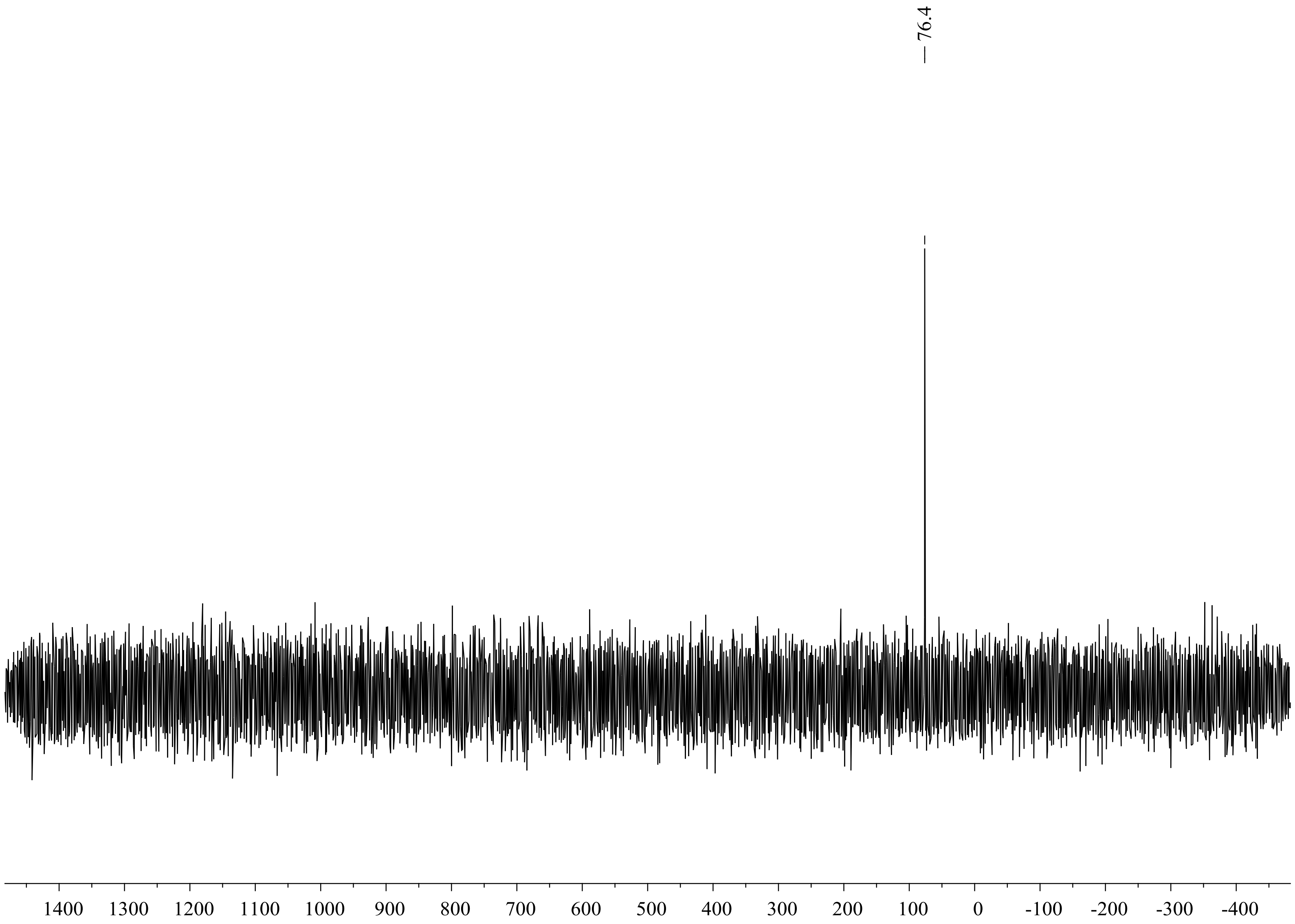

^{1}H NMR. *n*-Butyl(phenylethynyl)selane (5c)

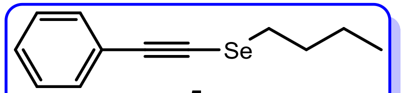

5c

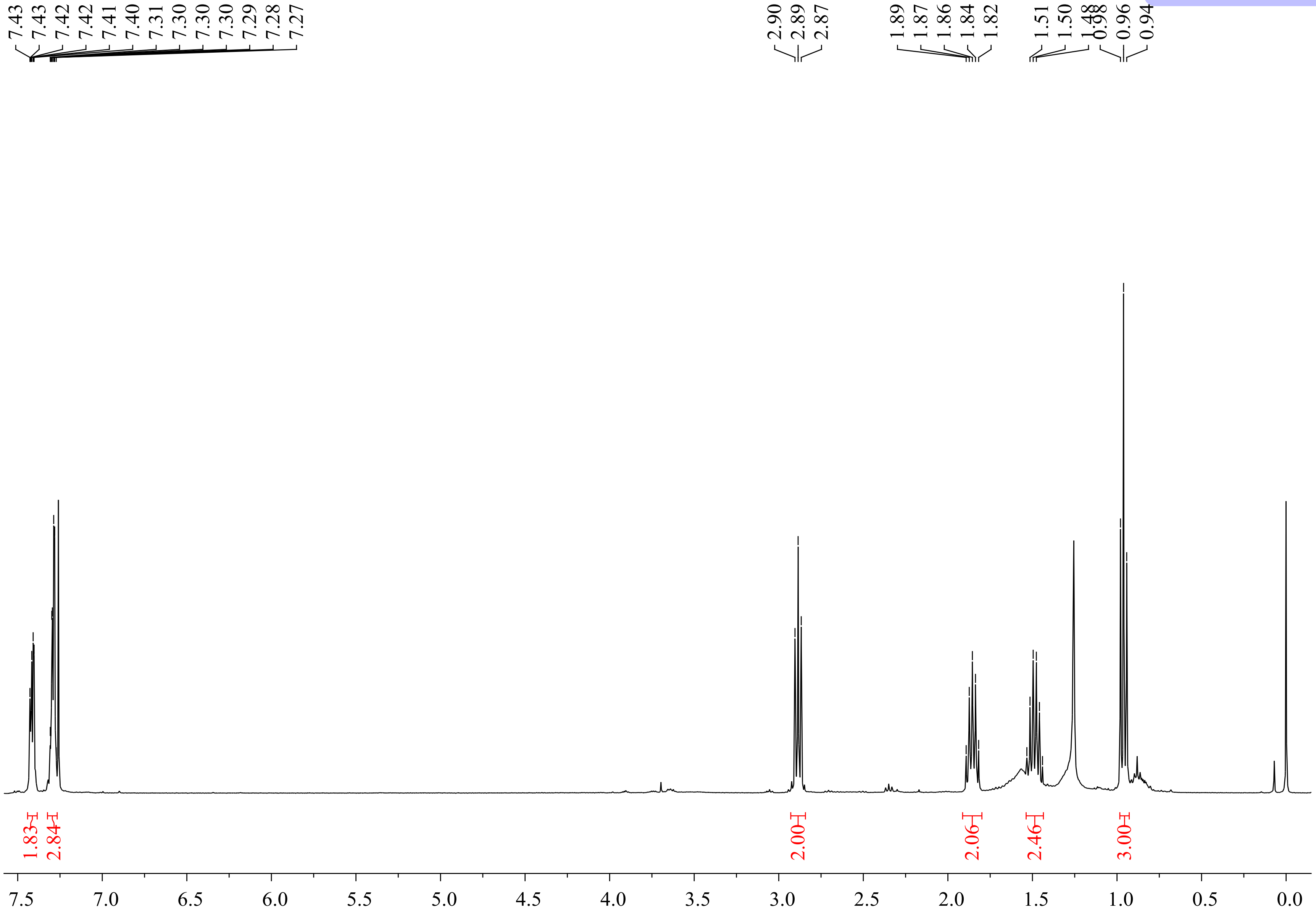

^{13}C NMR. *n*-Butyl(phenylethynyl)selane (5c)

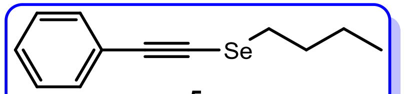

5c

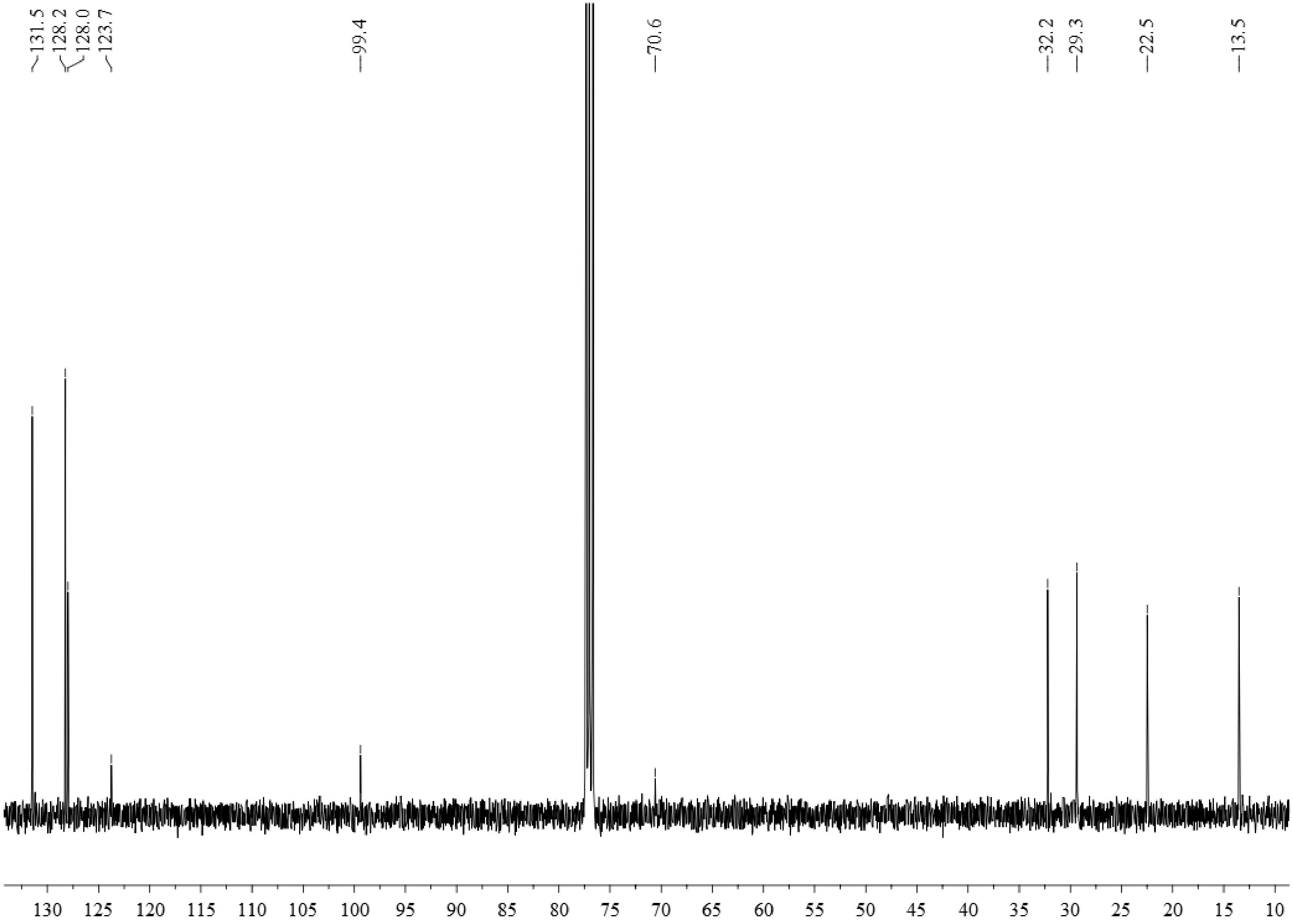

^{77}Se NMR. *n*-Butyl(phenylethynyl)selane (5c)

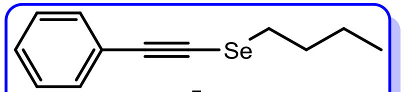

5c

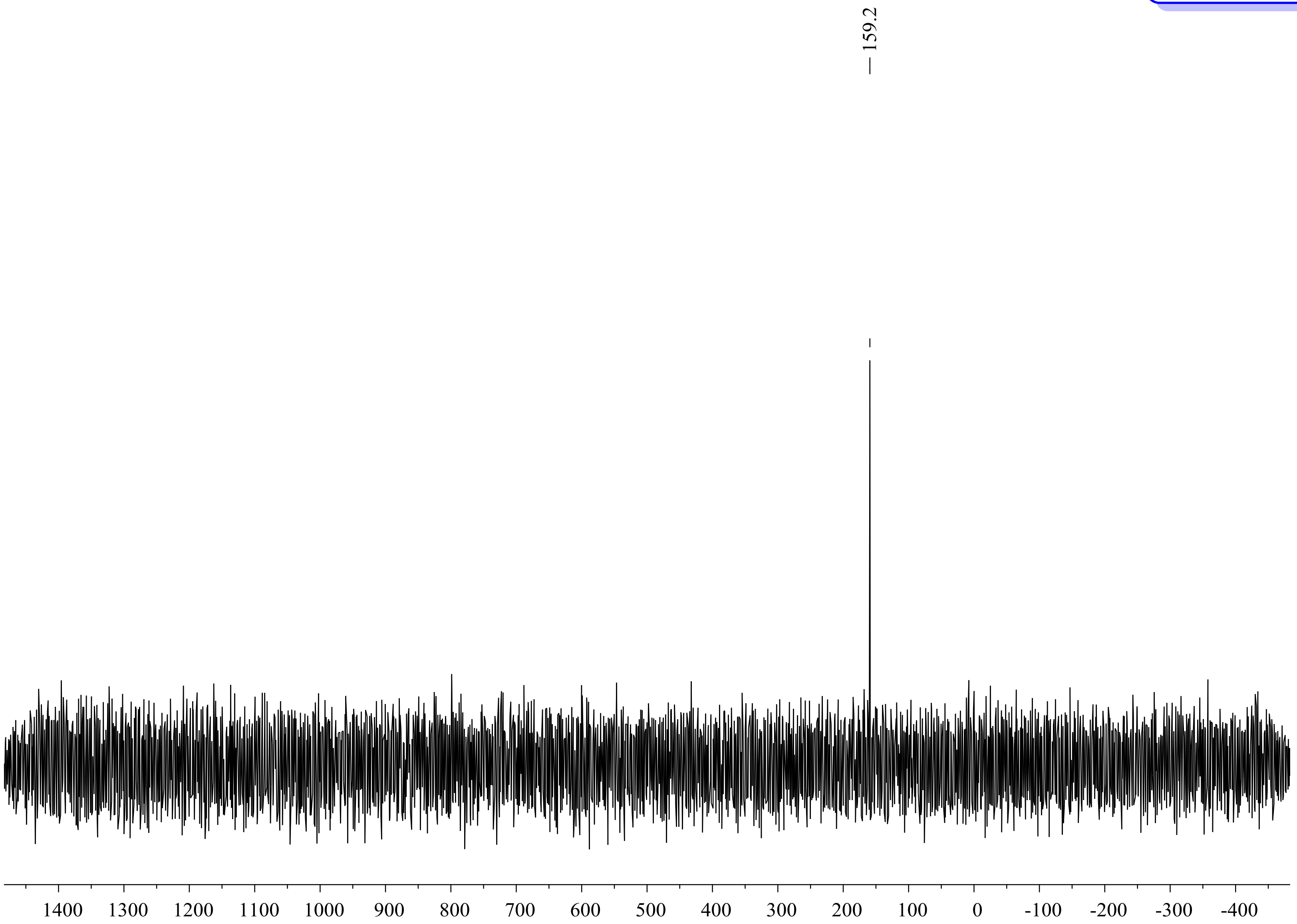

^{1}H NMR. Hex-5-en-1-yl(phenylethynyl)selane (5d)

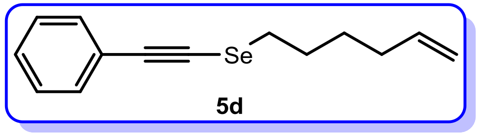
5d

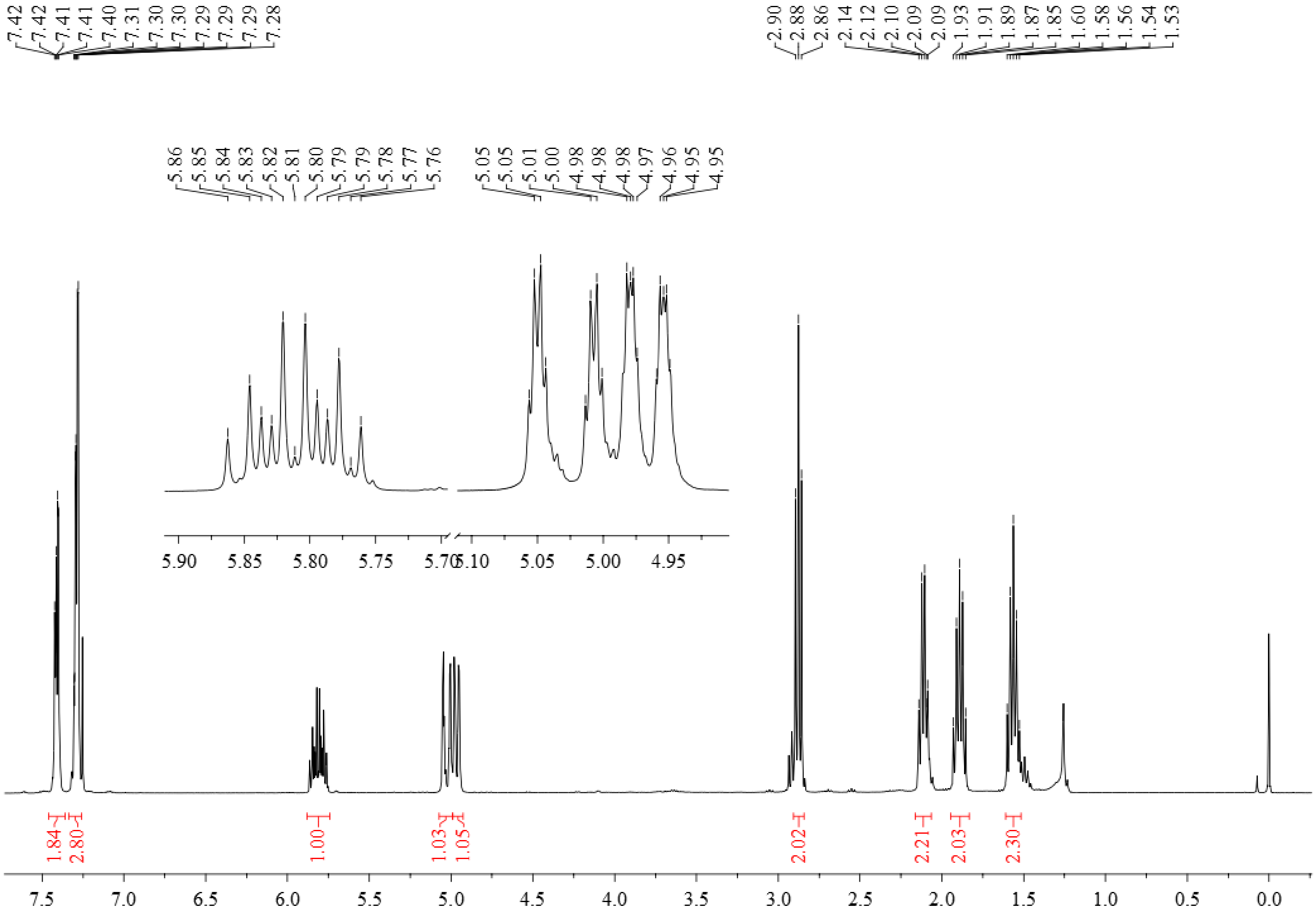

^{13}C NMR. Hex-5-en-1-yl(phenylethynyl)selane (5d)

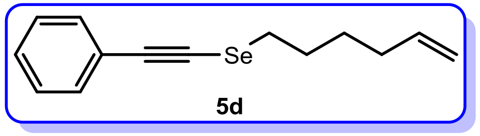

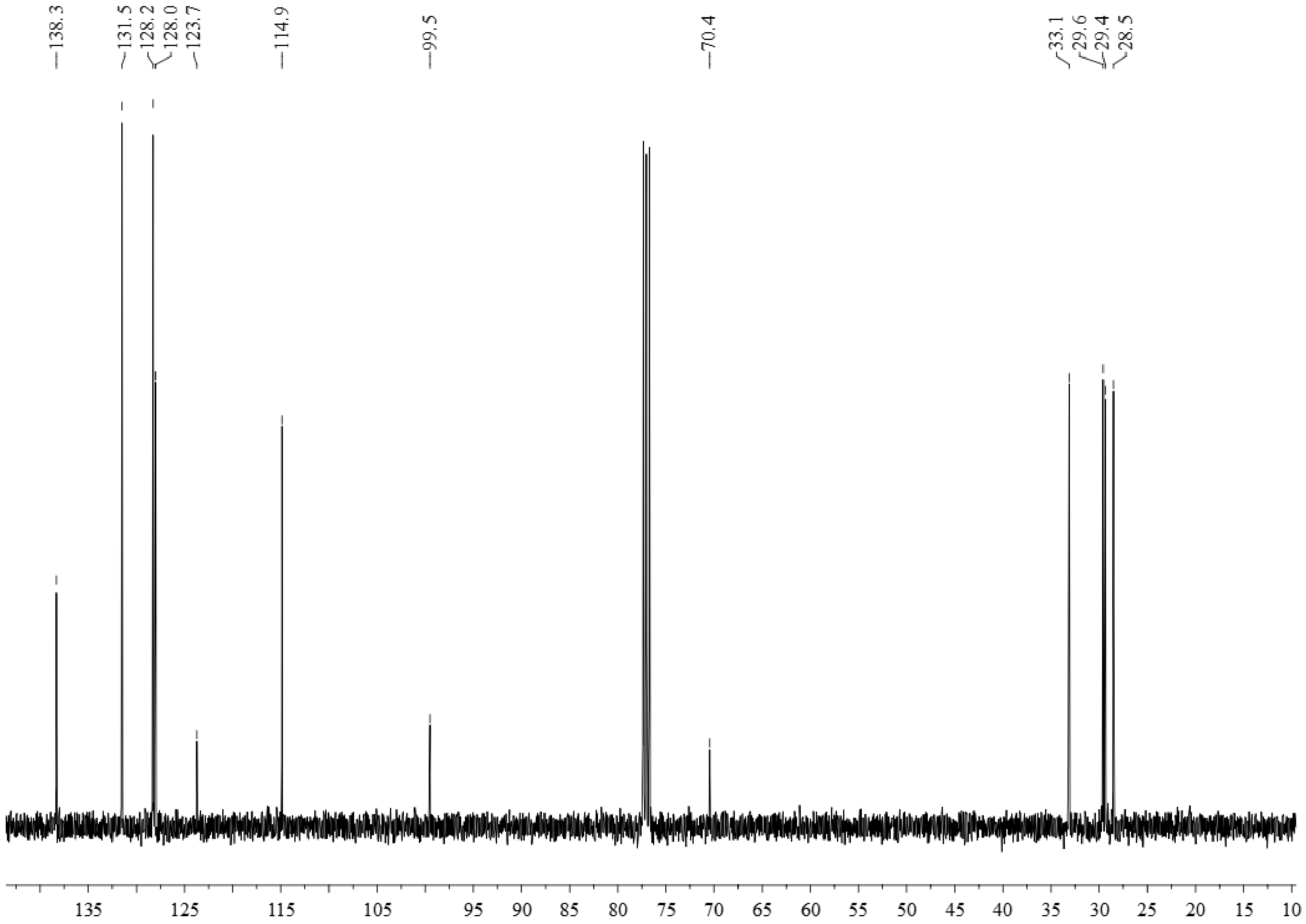

^{77}Se NMR. Hex-5-en-1-yl(phenylethynyl)selane (5d)

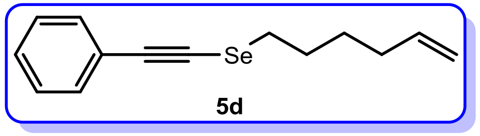

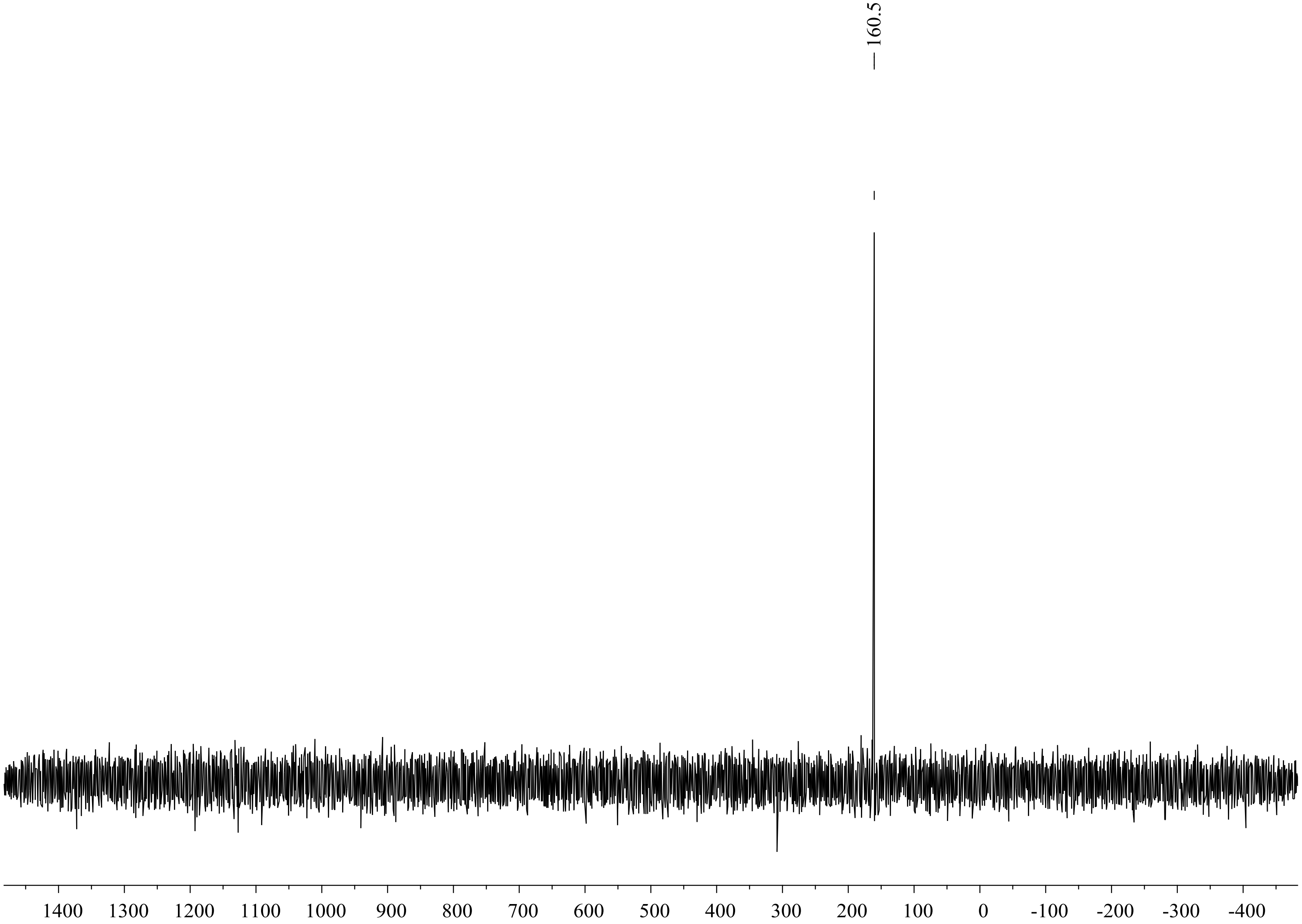

^{1}H NMR.(Cyclohexylmethyl)(phenylethynyl)selane (5e)

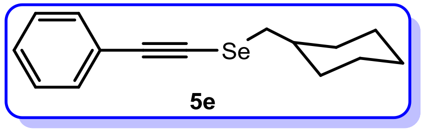

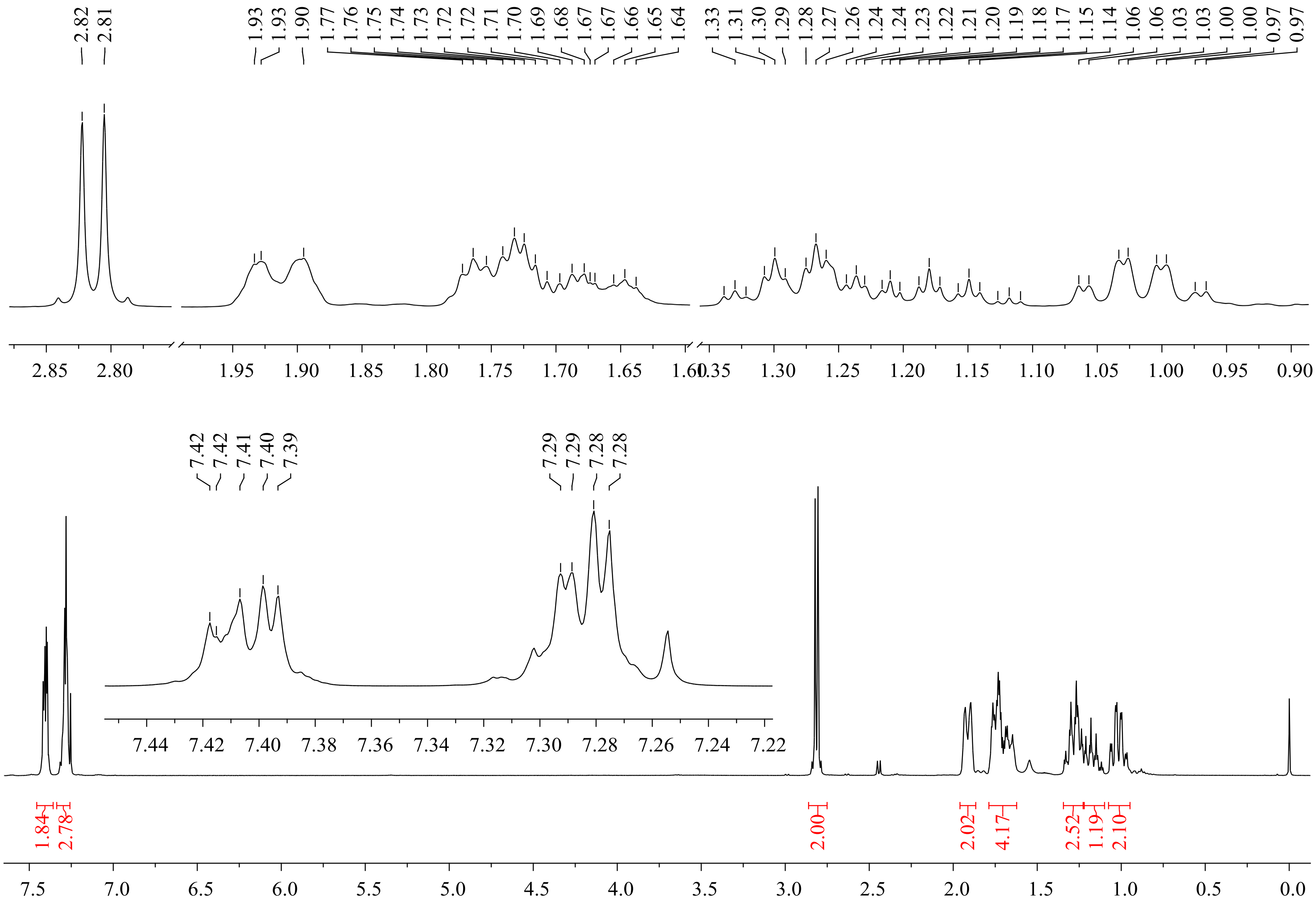

^{13}CNMR.(Cyclohexylmethyl)(phenylethynyl)selane (5e)

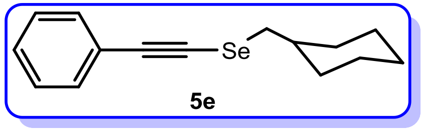

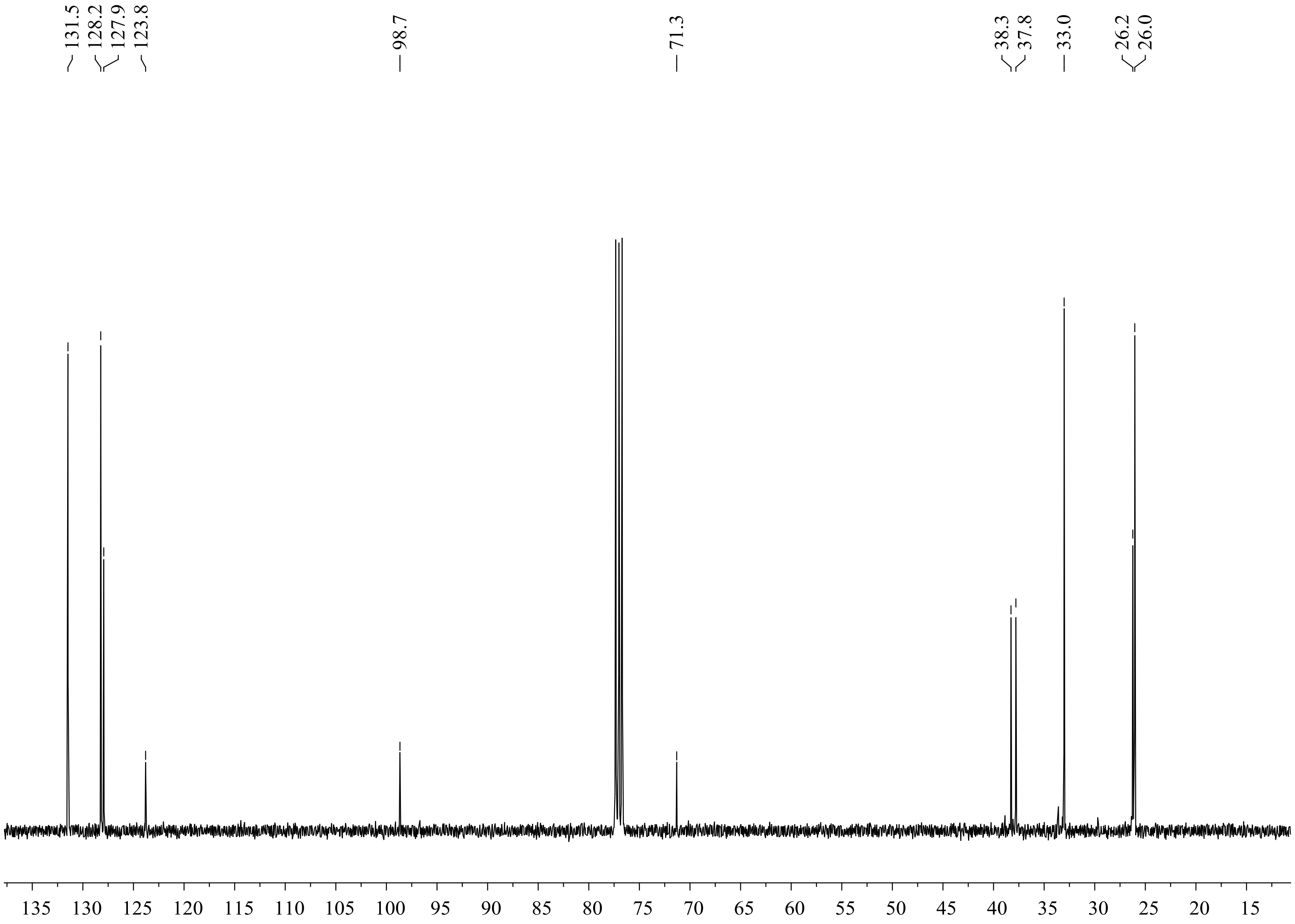

^{77}SeNMR.(Cyclohexylmethyl)(phenylethynyl)selane (5e)

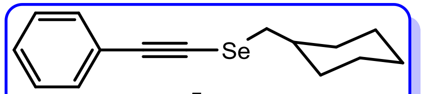

5e

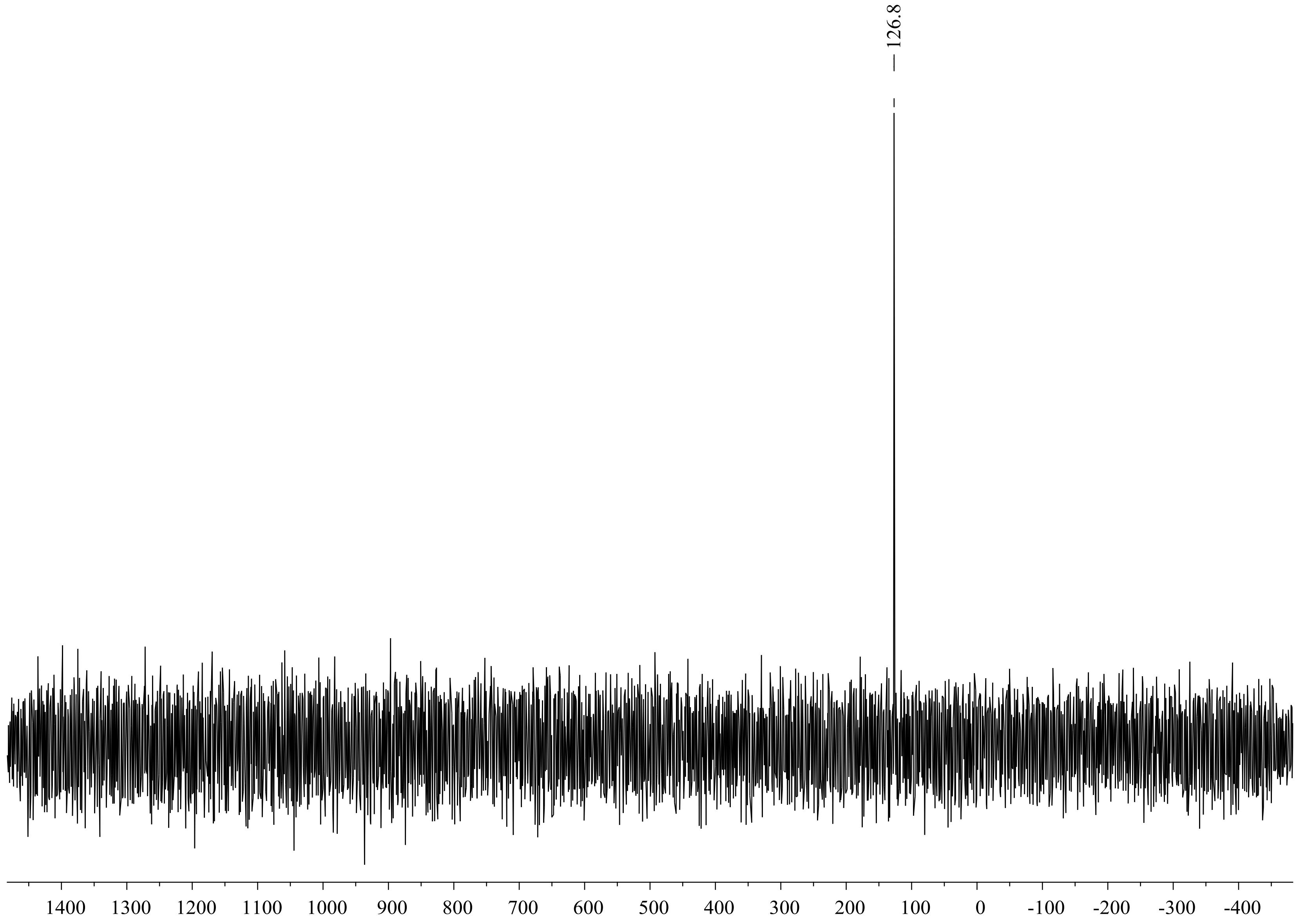

^{1}H NMR. *n*-Octyl(*p*-tolylethynyl)selane (5i)

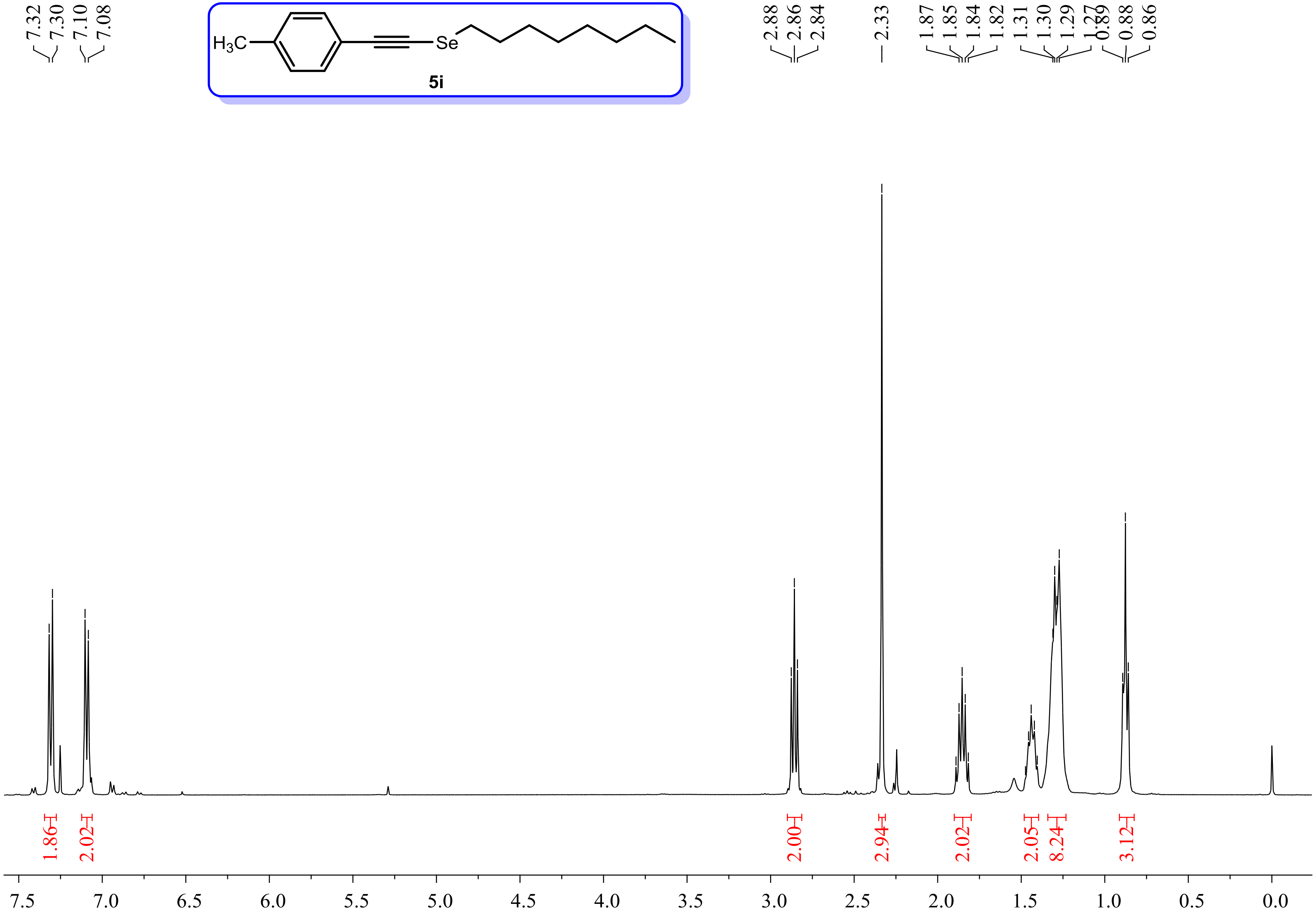

^{13}C NMR. *n*-Octyl(*p*-tolylethynyl)selane (5i)

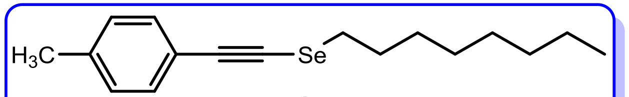

5i

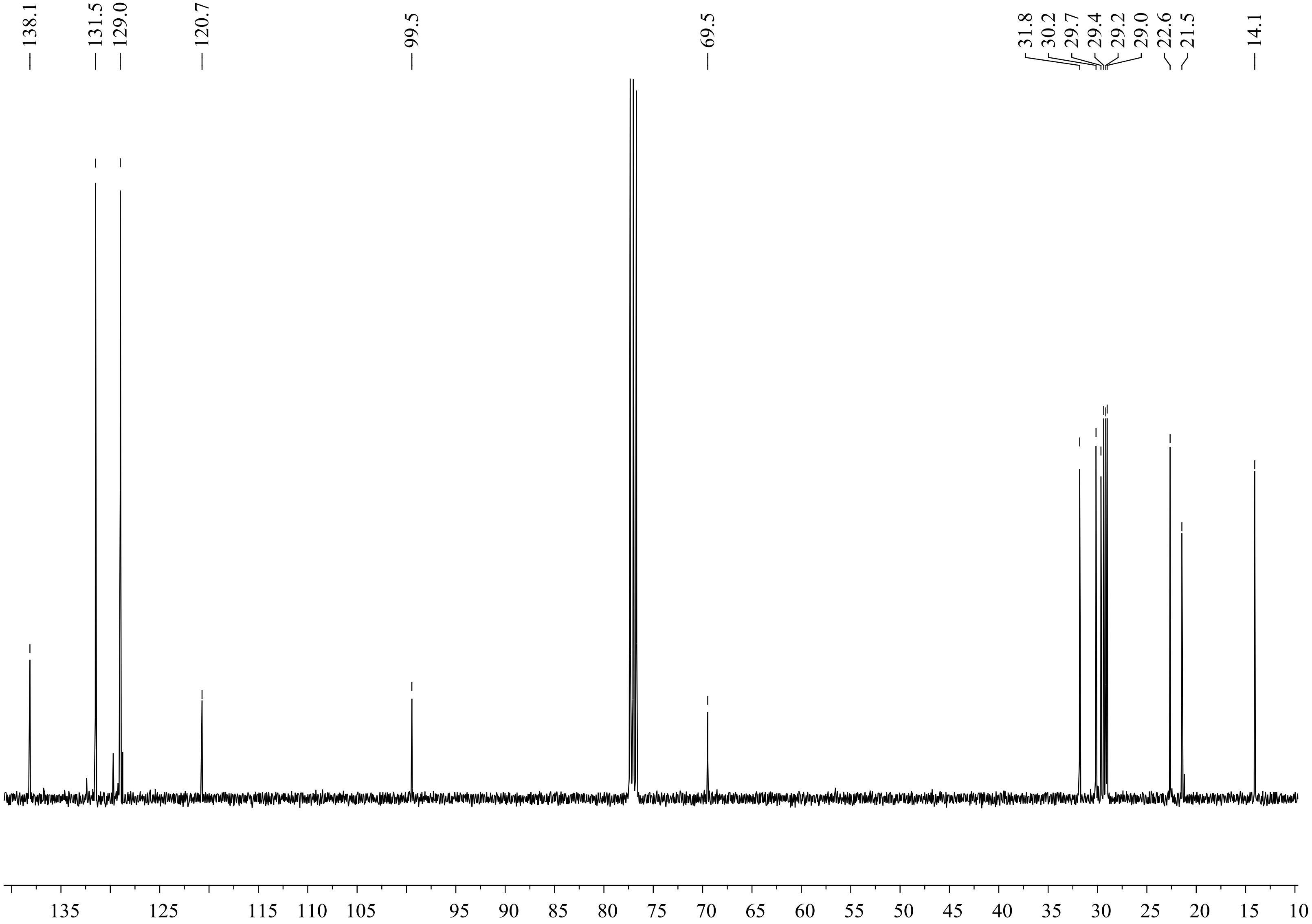

^{77}Se NMR. *n*-Octyl(*p*-tolylethynyl)selane (5i)

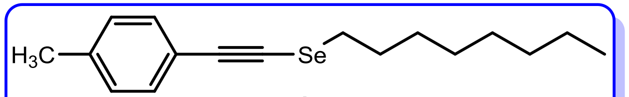

5i

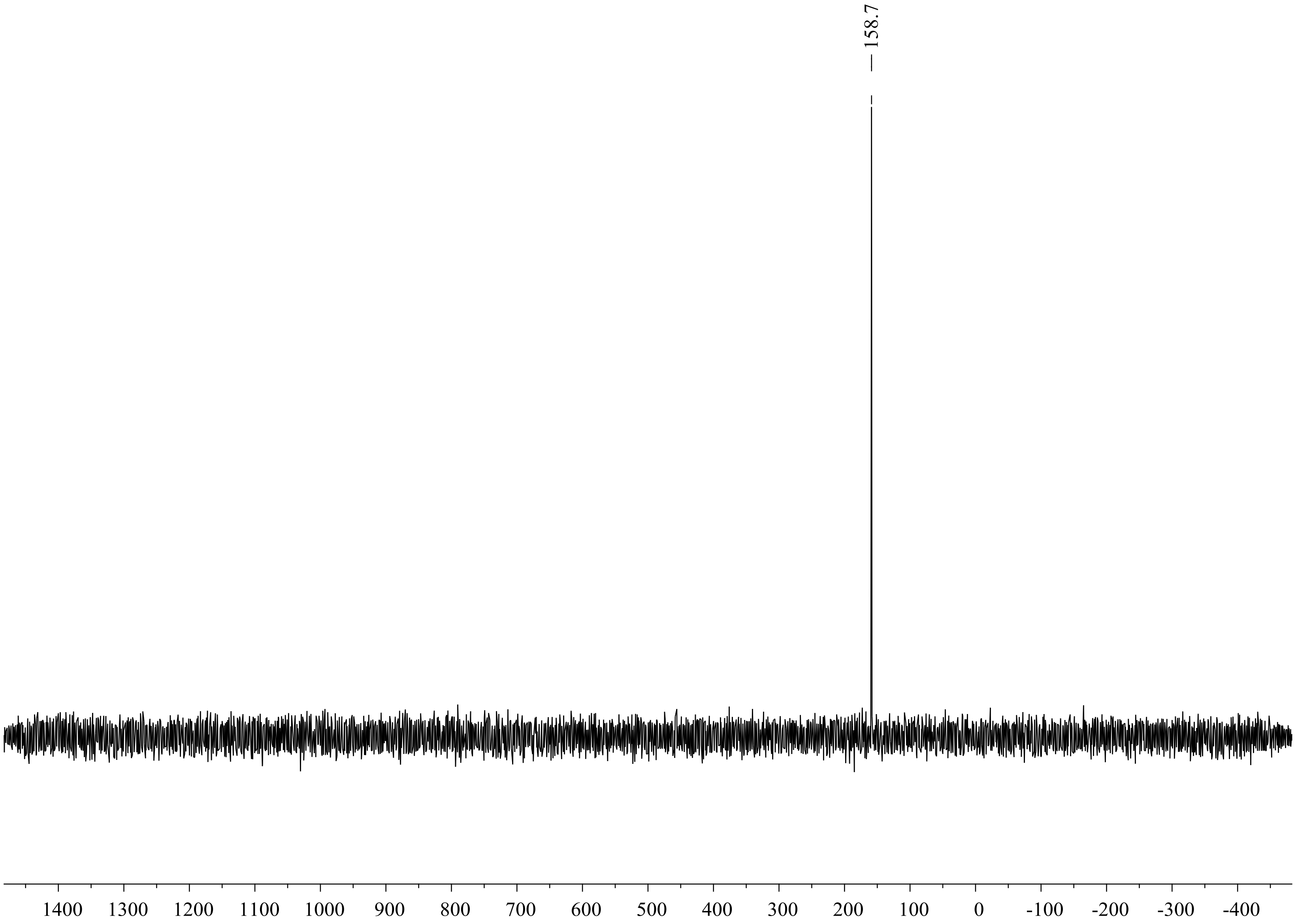

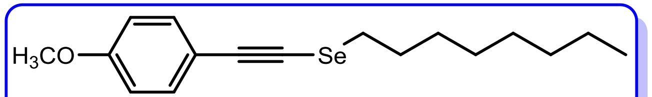

5j

^{1}H NMR.((4-Methoxyphenyl)ethynyl)(*n*-octyl)selane (5j)

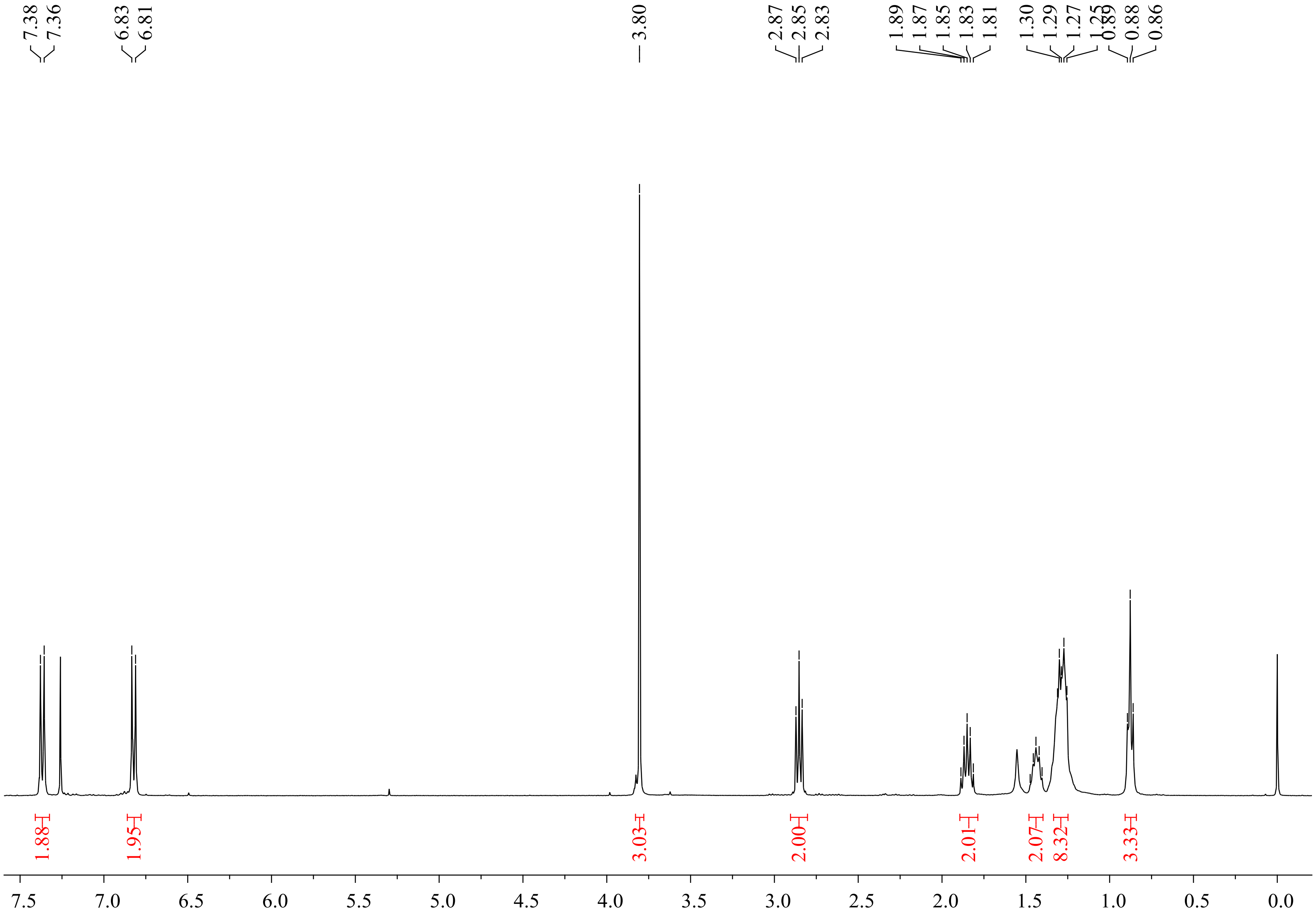

^{13}CNMR.((4-Methoxyphenyl)ethynyl)(*n*-octyl)selane (5j)

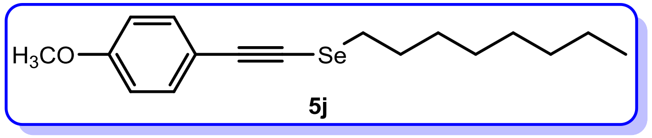

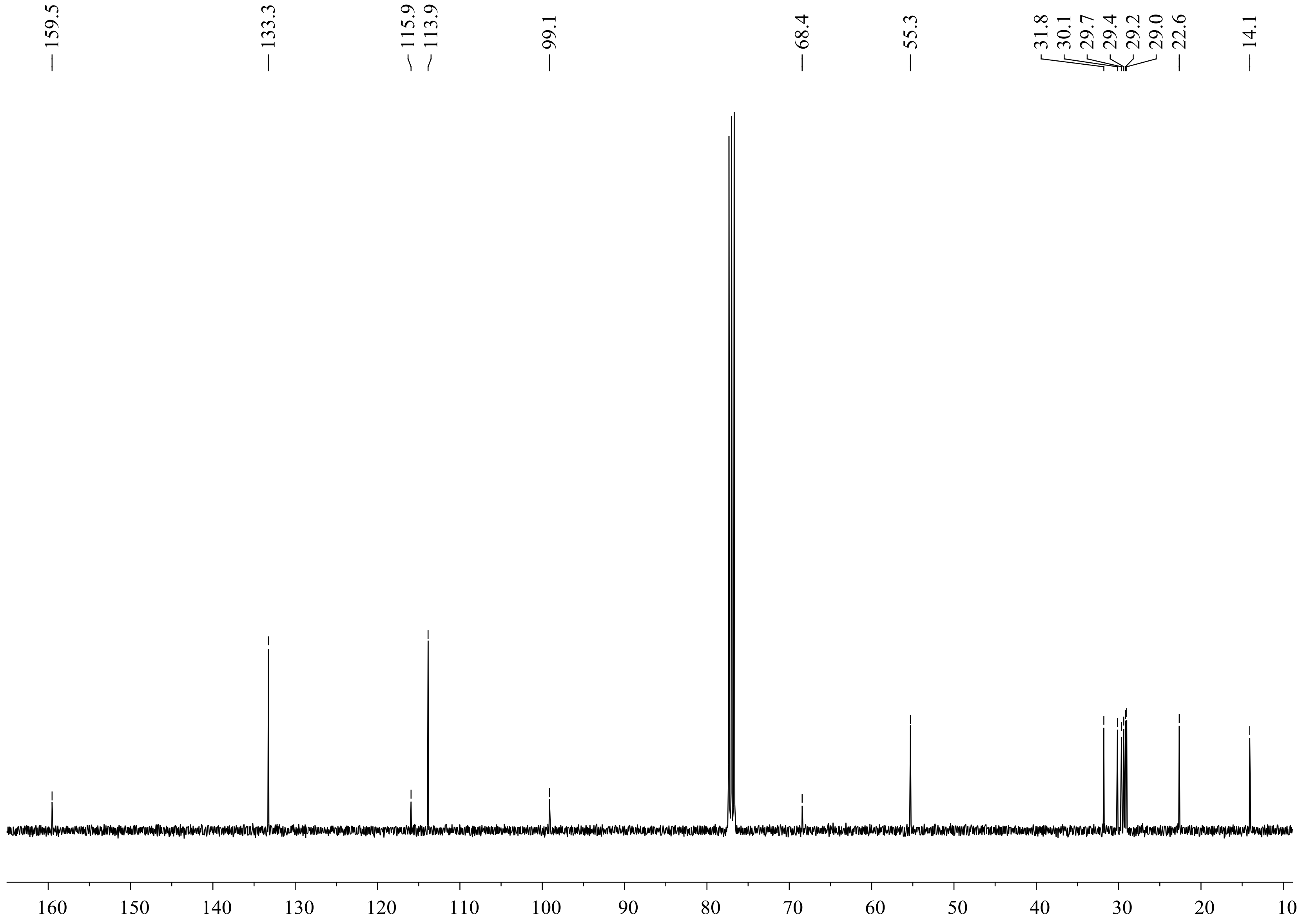

^{77}SeNMR.((4-Methoxyphenyl)ethynyl)(*n*-octyl)selane (5j)

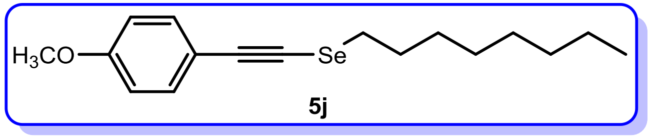

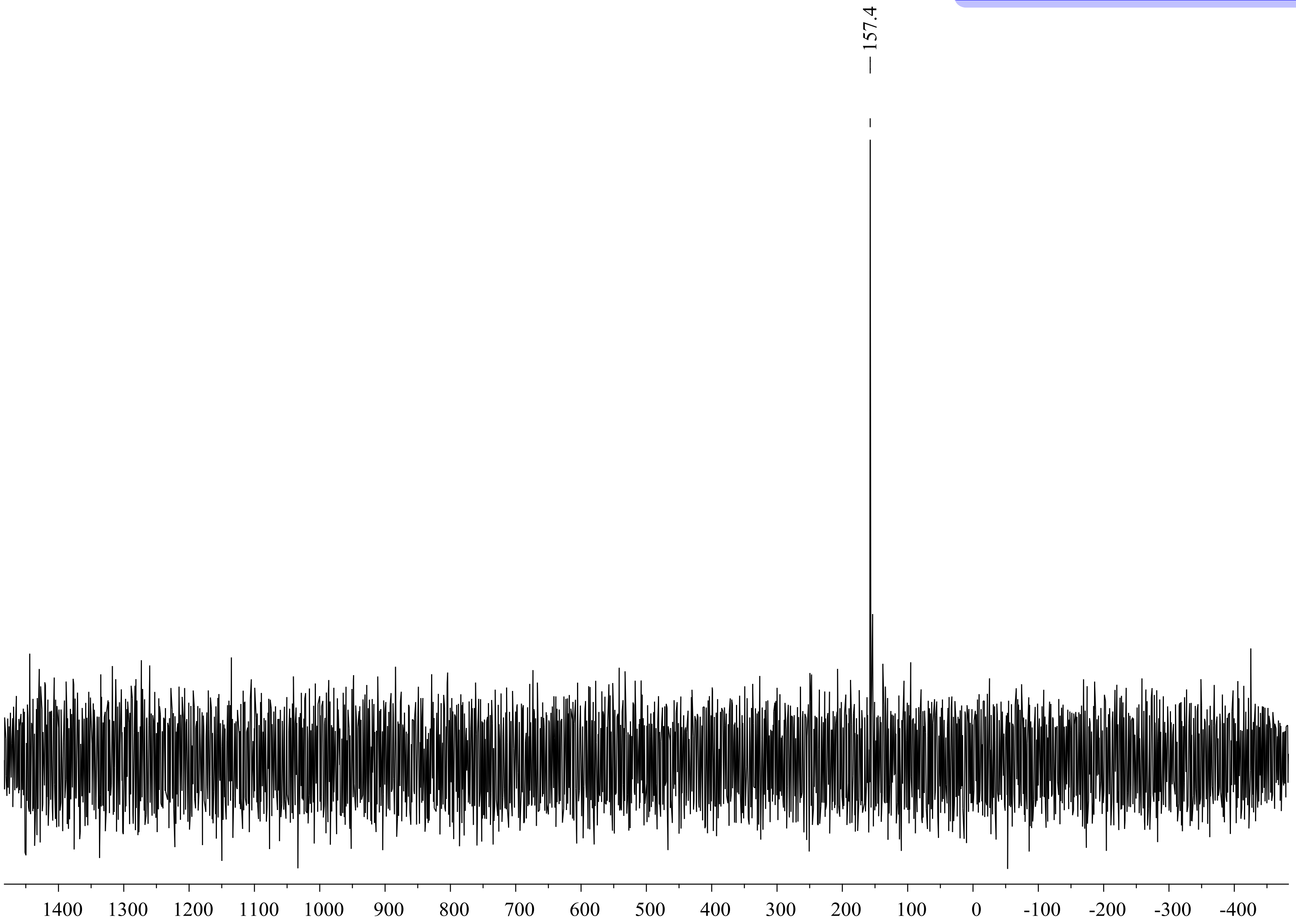

^{1}H NMR. ((4-Chlorophenyl)ethynyl)(*n*-octyl)selane (5k)

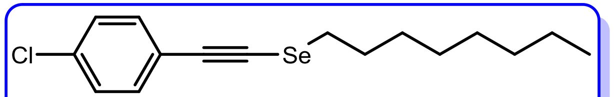

5k

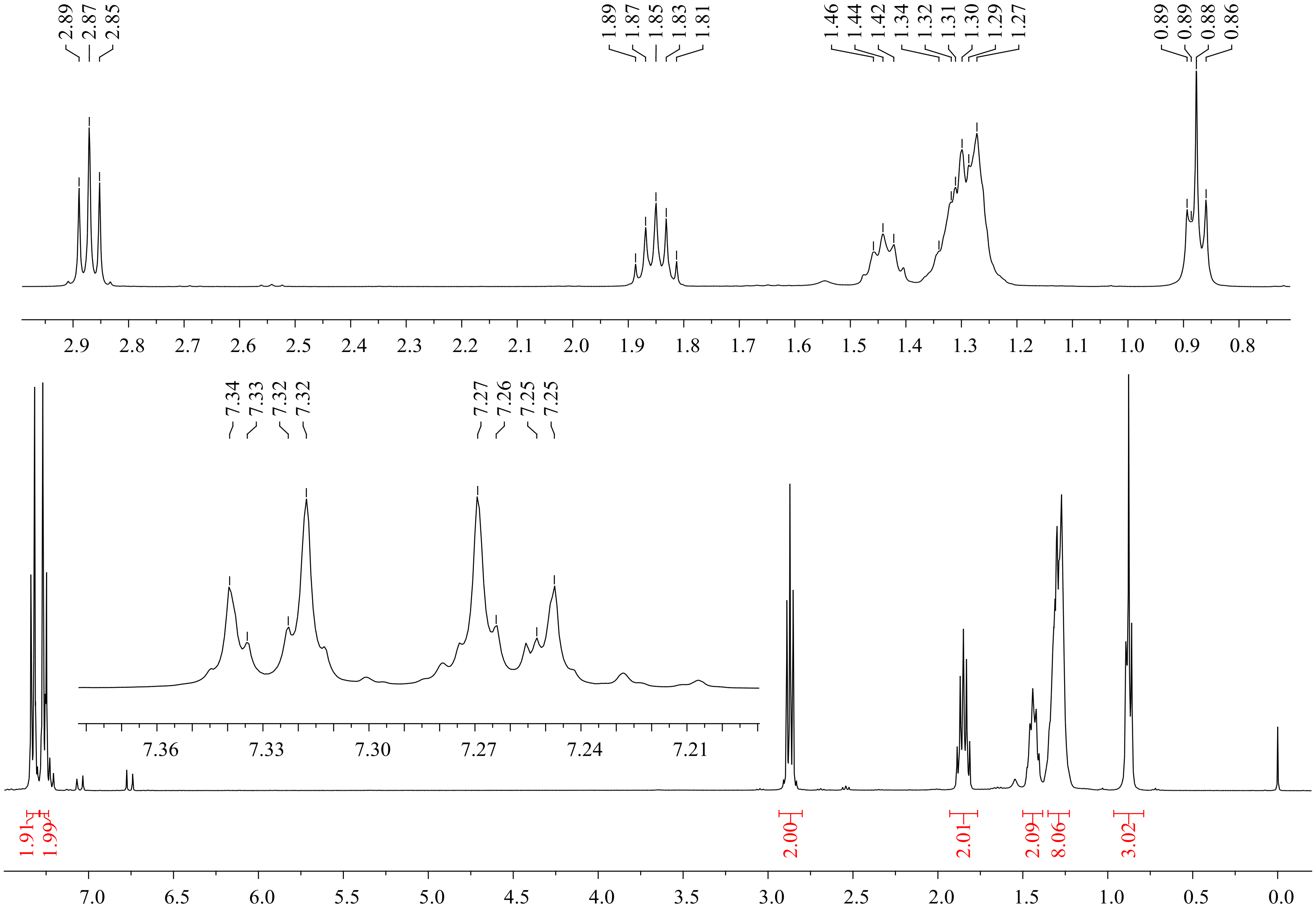

^{13}C NMR. ((4-Chlorophenyl)ethynyl)(*n*-octyl)selane (5k)

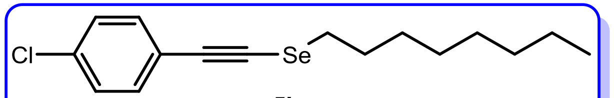

5k

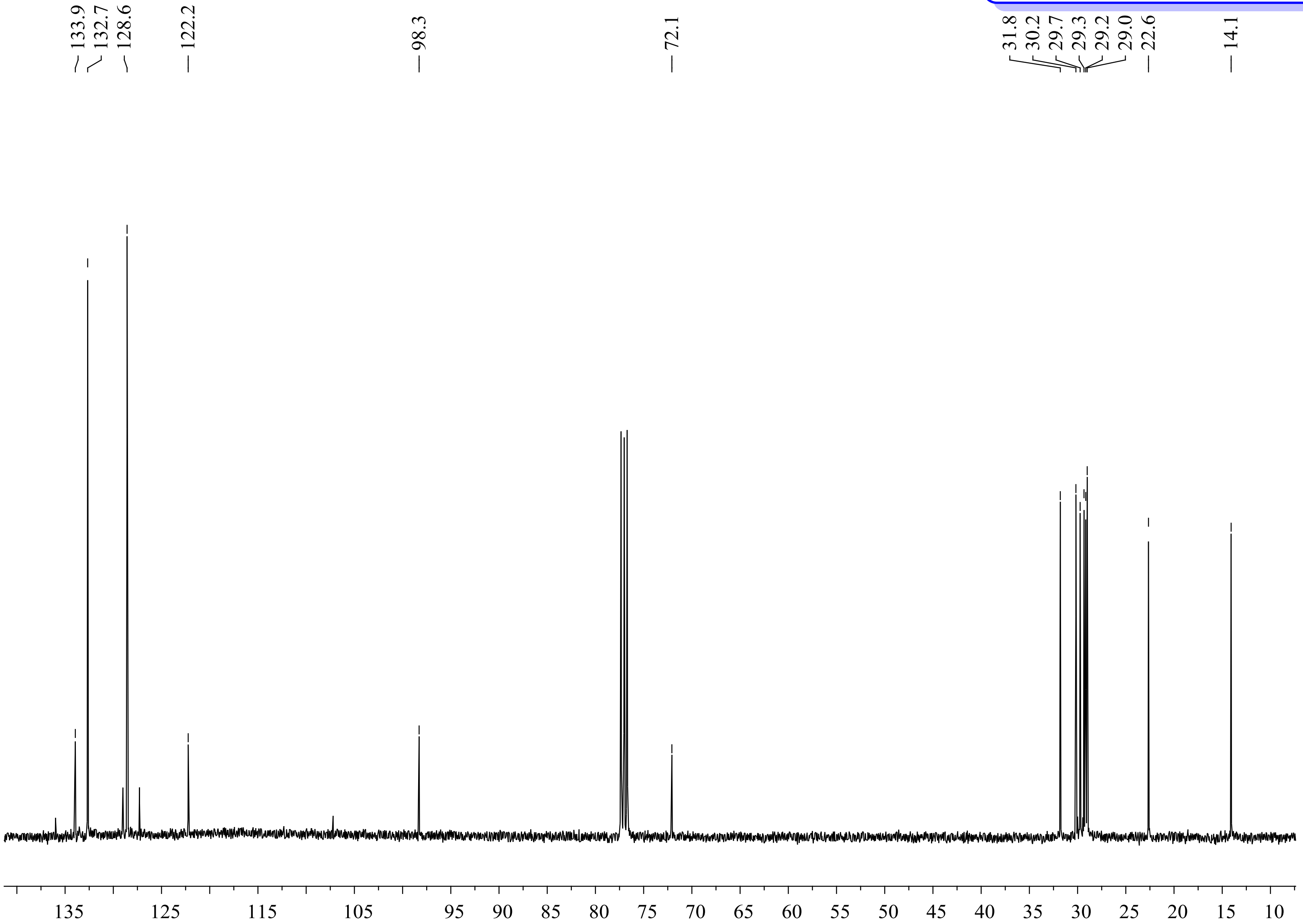

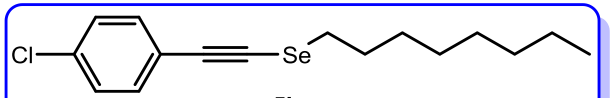

5k

[77]**Se NMR. ((4-Chlorophenyl)ethynyl)(*n*-octyl)selane (5k)**

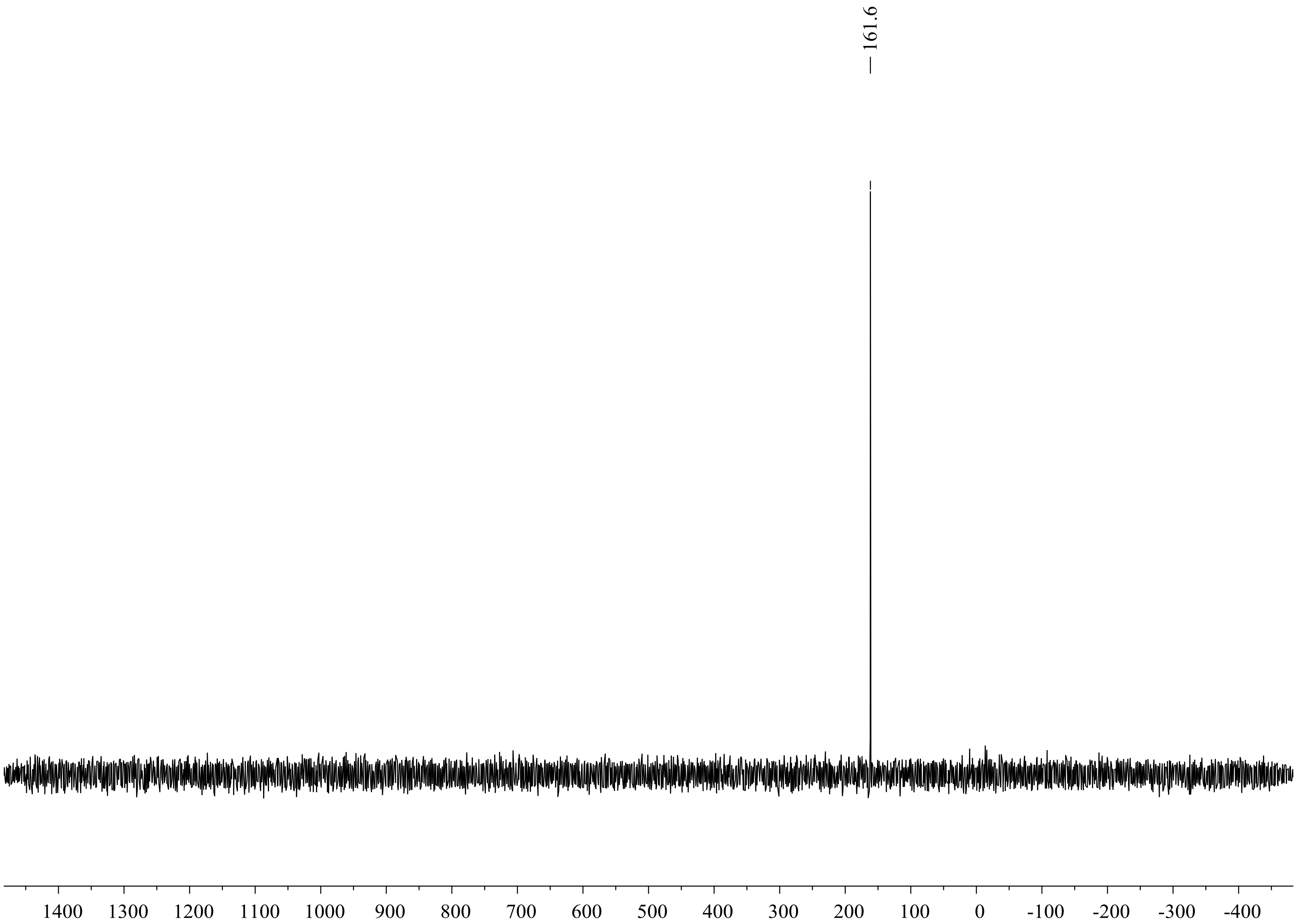

^{1}H NMR. ((2-Bromophenyl)ethynyl)(*n*-octyl)selane (5l)

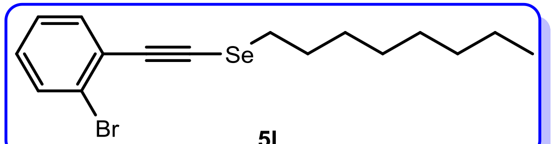

5l

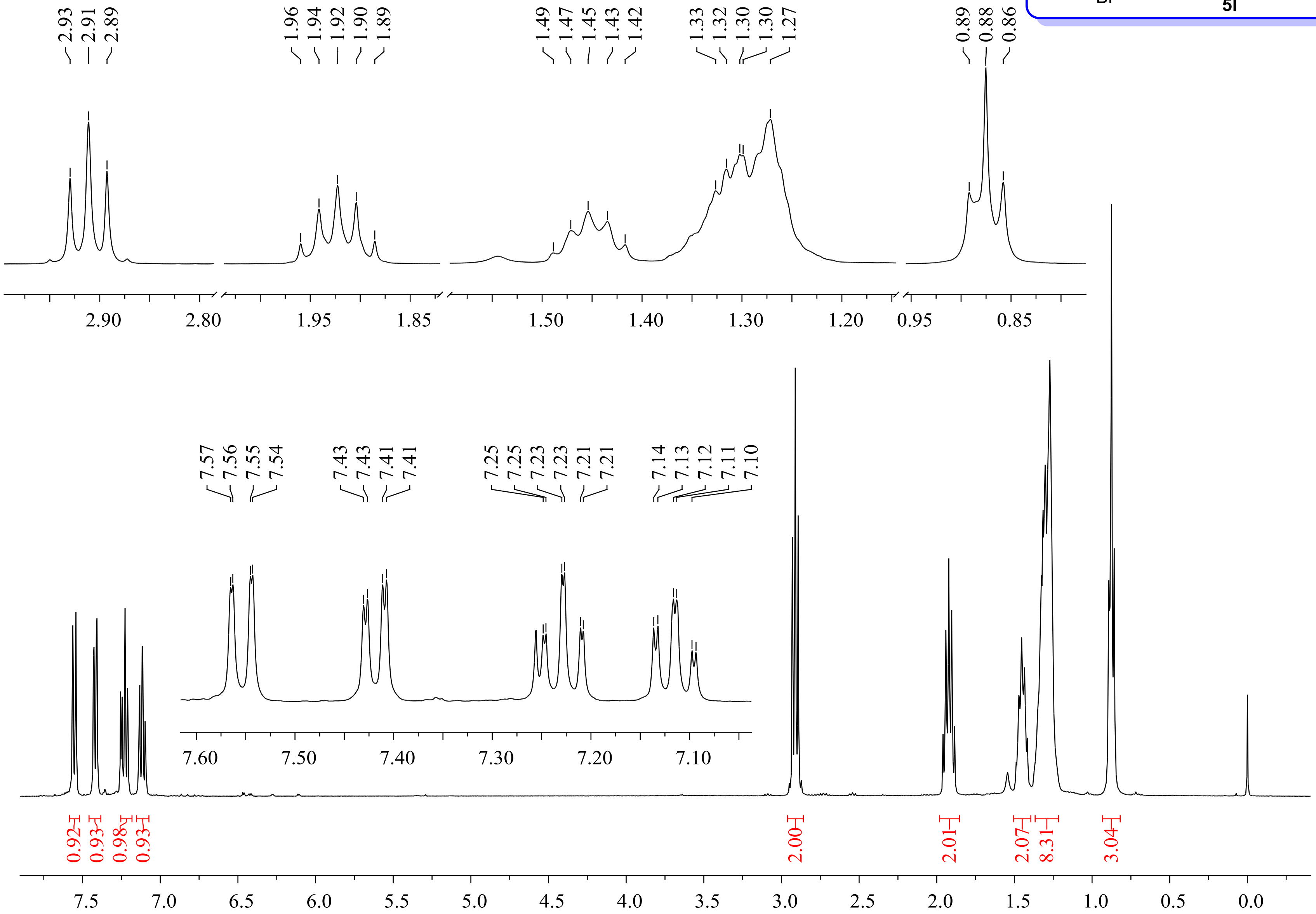

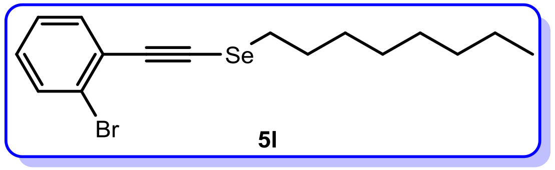

^{13}C NMR. ((2-Bromophenyl)ethynyl)(*n*-octyl)selane (5l)

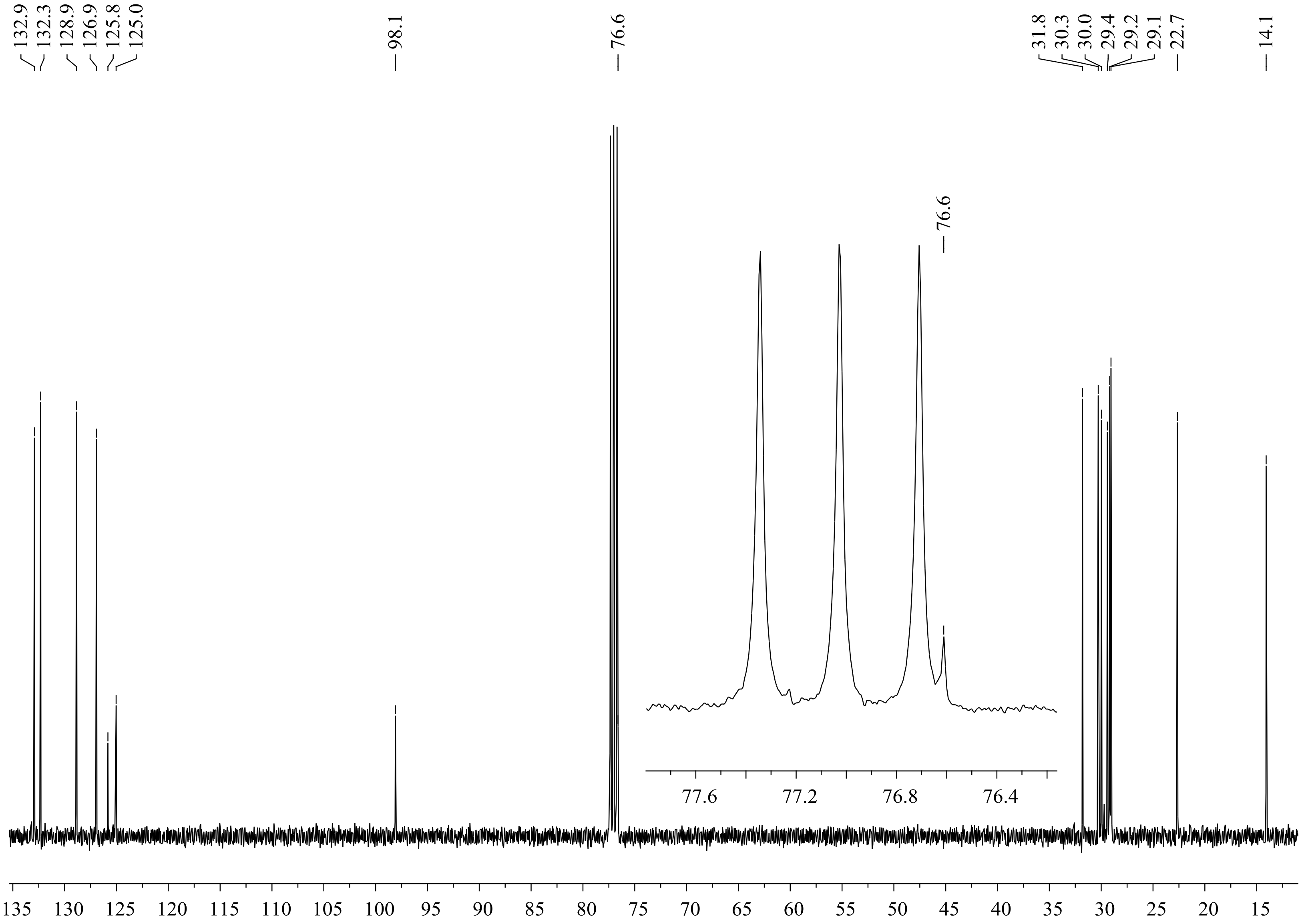

^{77}**Se NMR. ((2-Bromophenyl)ethynyl)(*n*-octyl)selane (5l)**

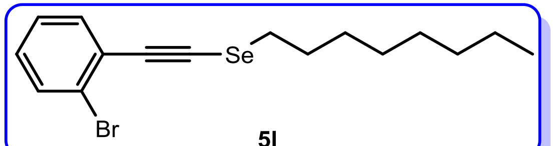

5l

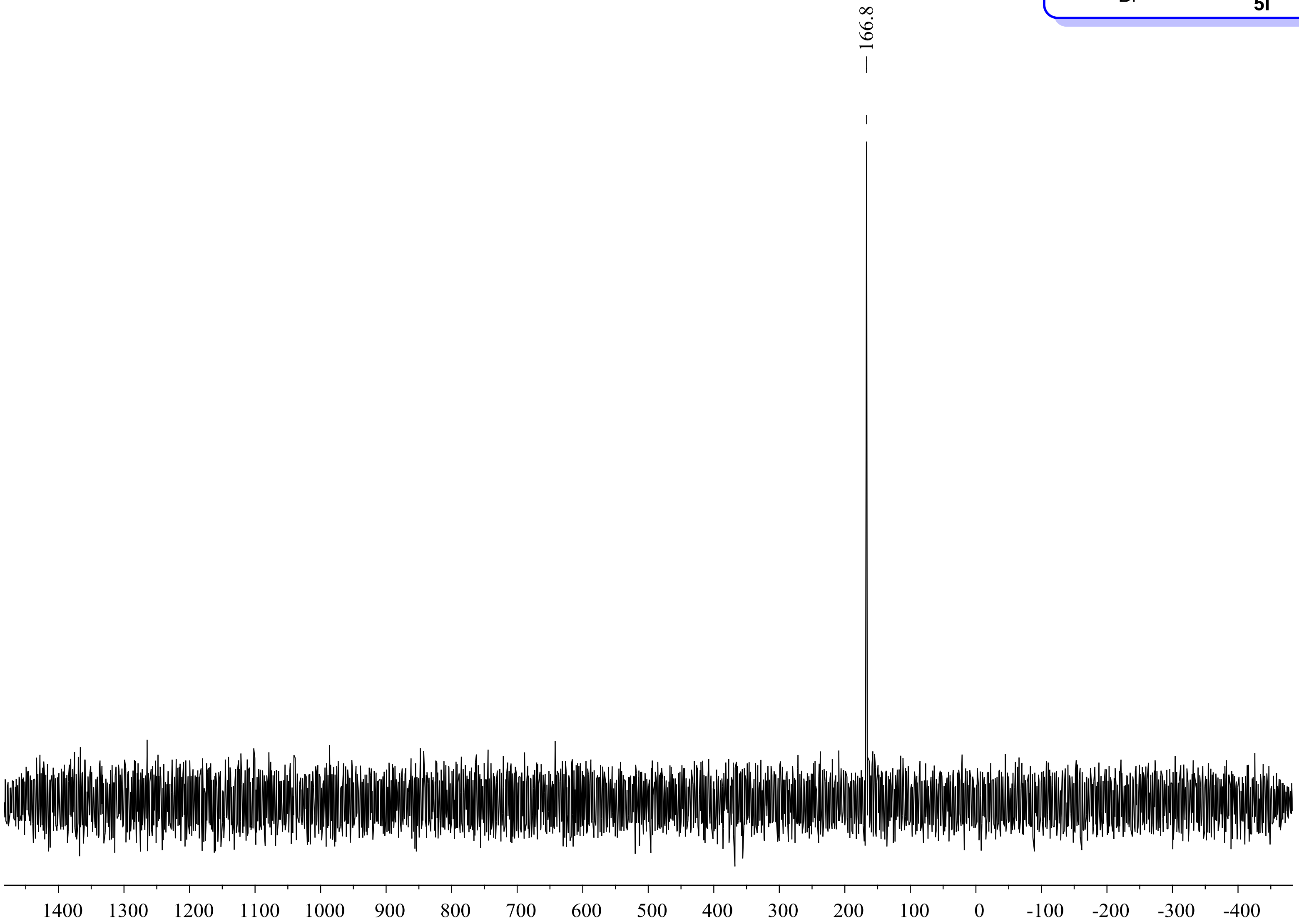

^{1}HNMR.(*E*,*Z*)-*n*-Octyl(4-phenylbut-3-en-1-yn-1-yl)selane (5n)

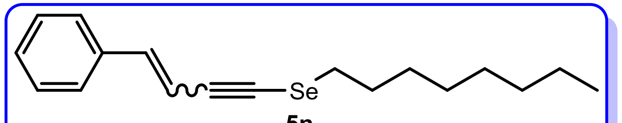

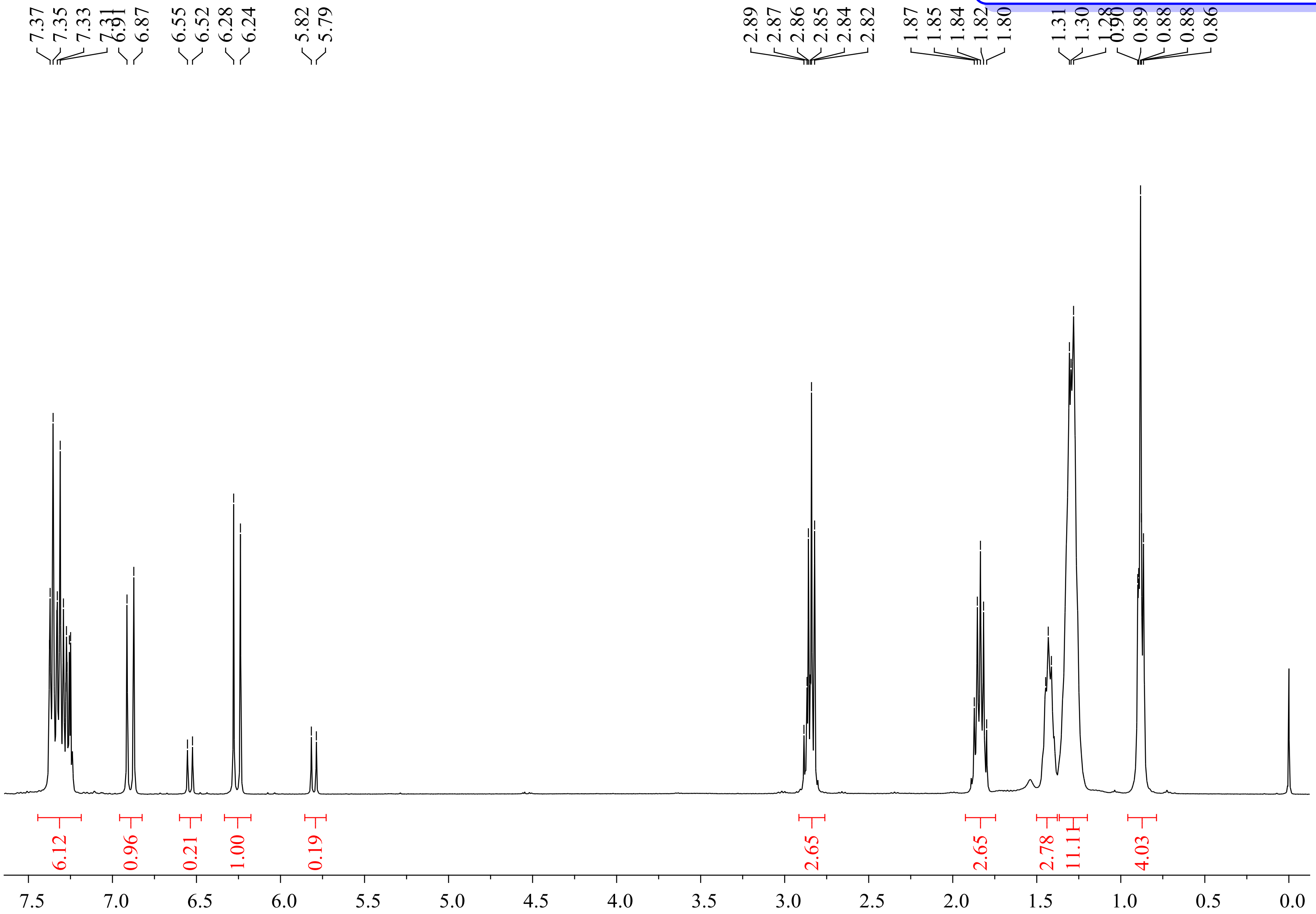

^{13}C NMR.(*E*,*Z*)-*n*-Octyl(4-phenylbut-3-en-1-yn-1-yl)selane (5n)

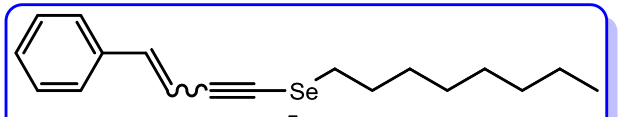

5n

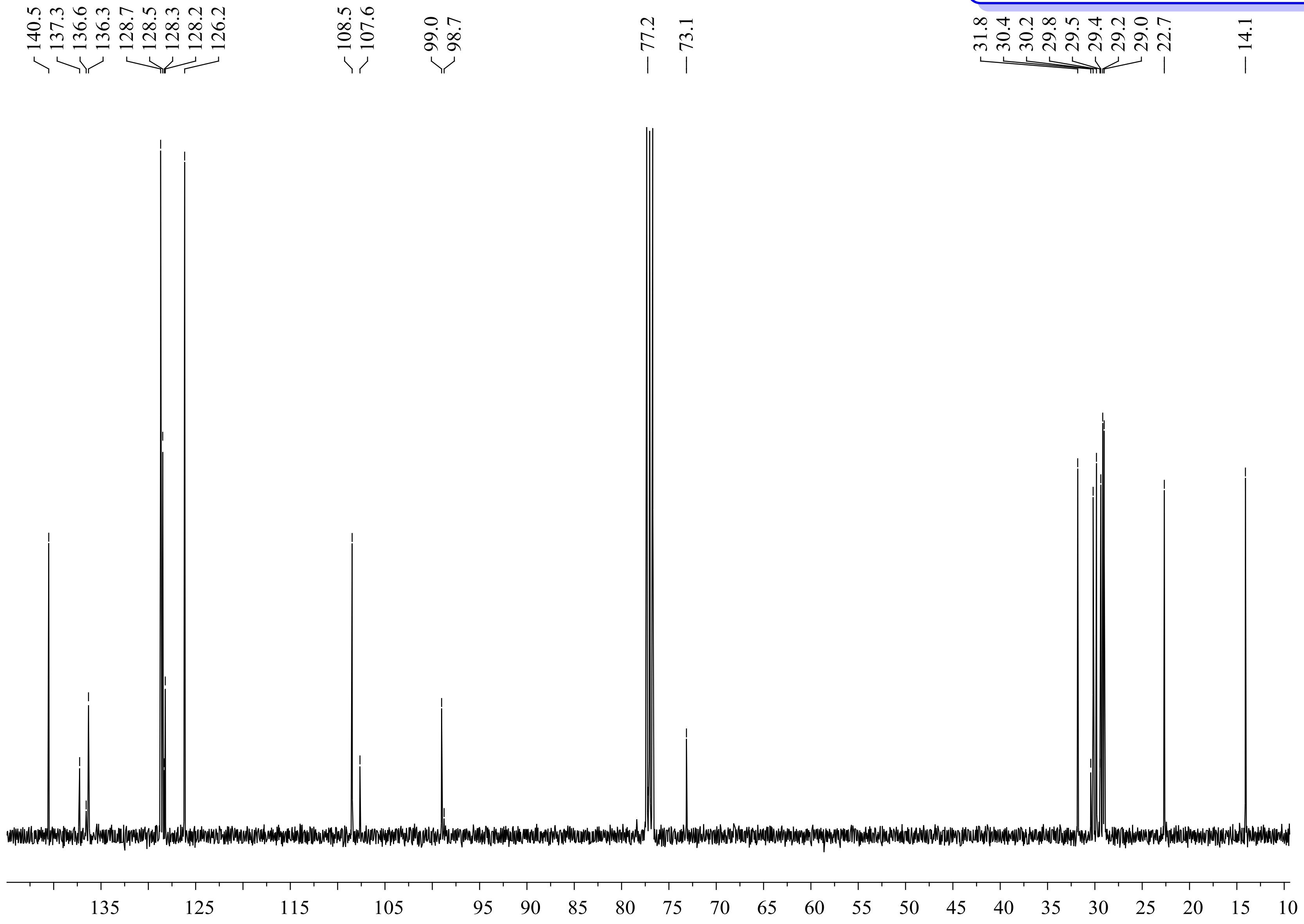

^{77}SeNMR.(*E*,*Z*)-*n*-Octyl(4-phenylbut-3-en-1-yn-1-yl)selane (5n)

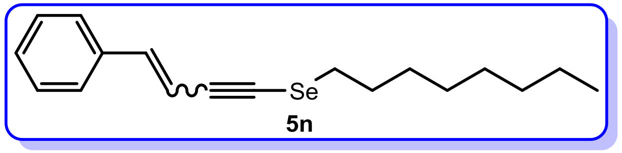

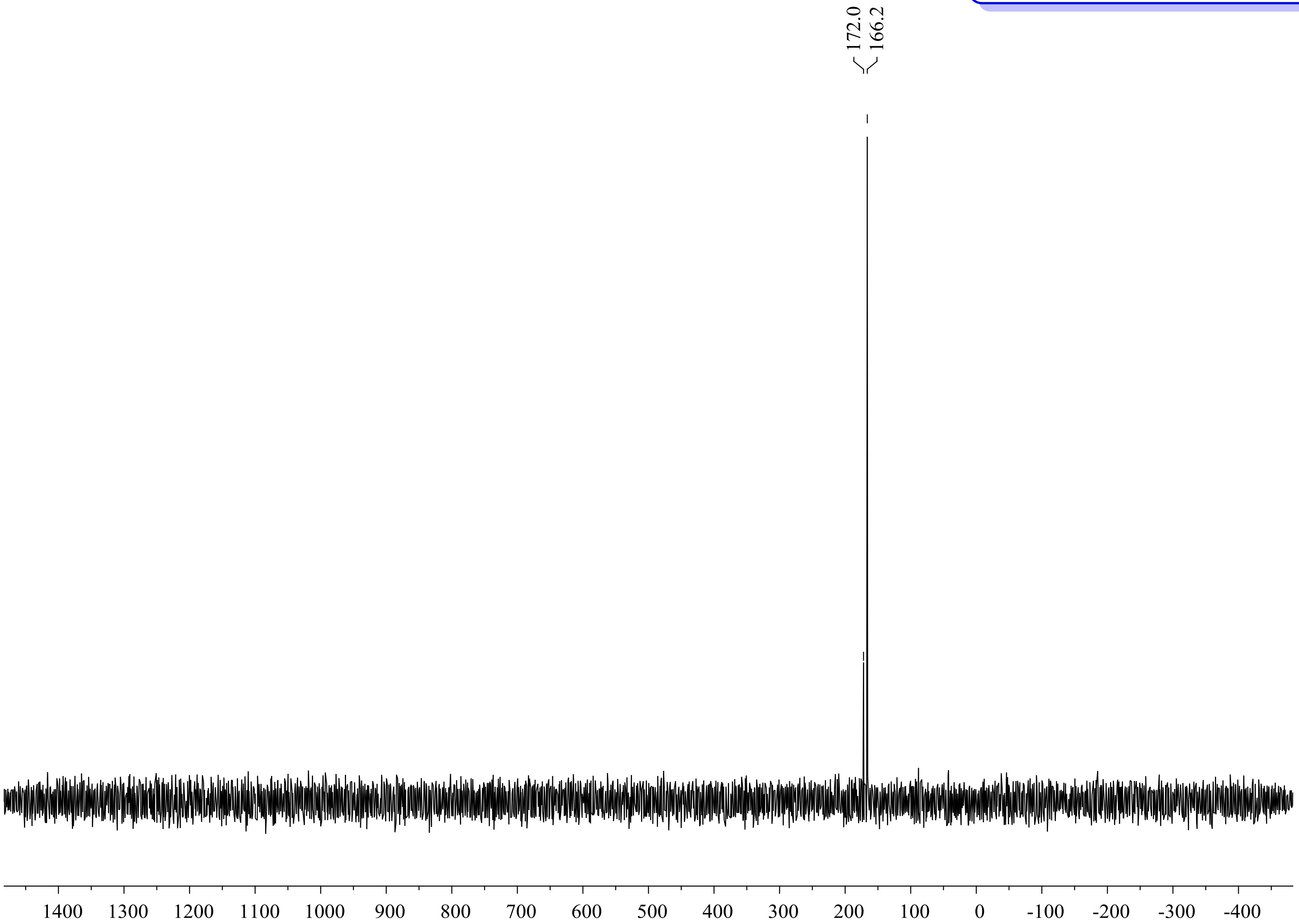

^{1}H NMR. ((2-Methoxyphenyl)ethynyl)(*n*-octyl)selane (5q)

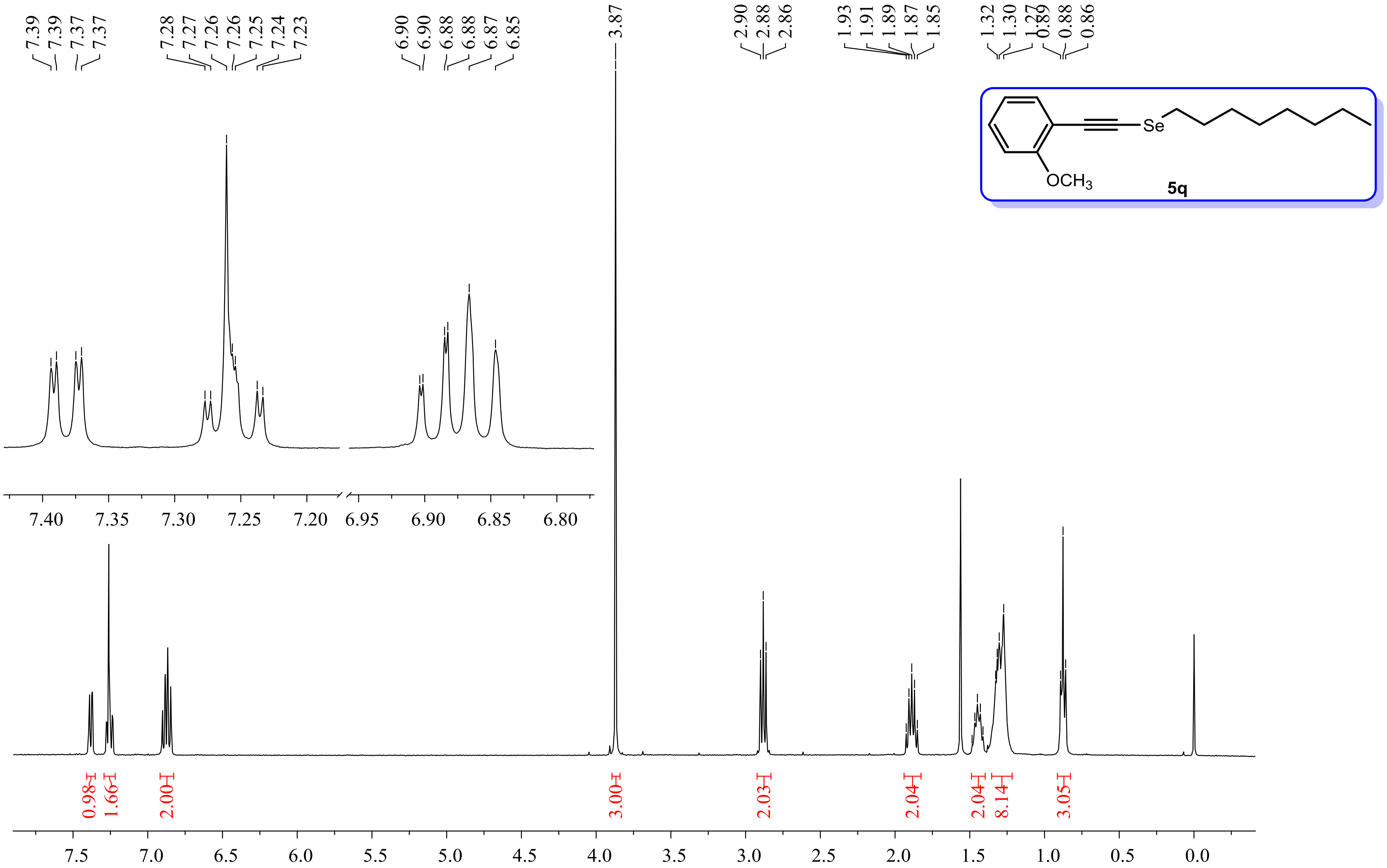

^{13}C NMR. ((2-Methoxyphenyl)ethynyl)(*n*-octyl)selane (5q)

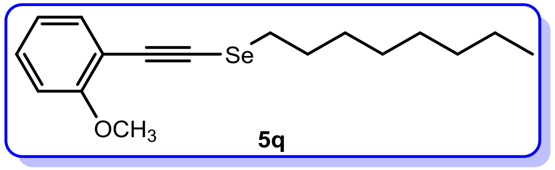

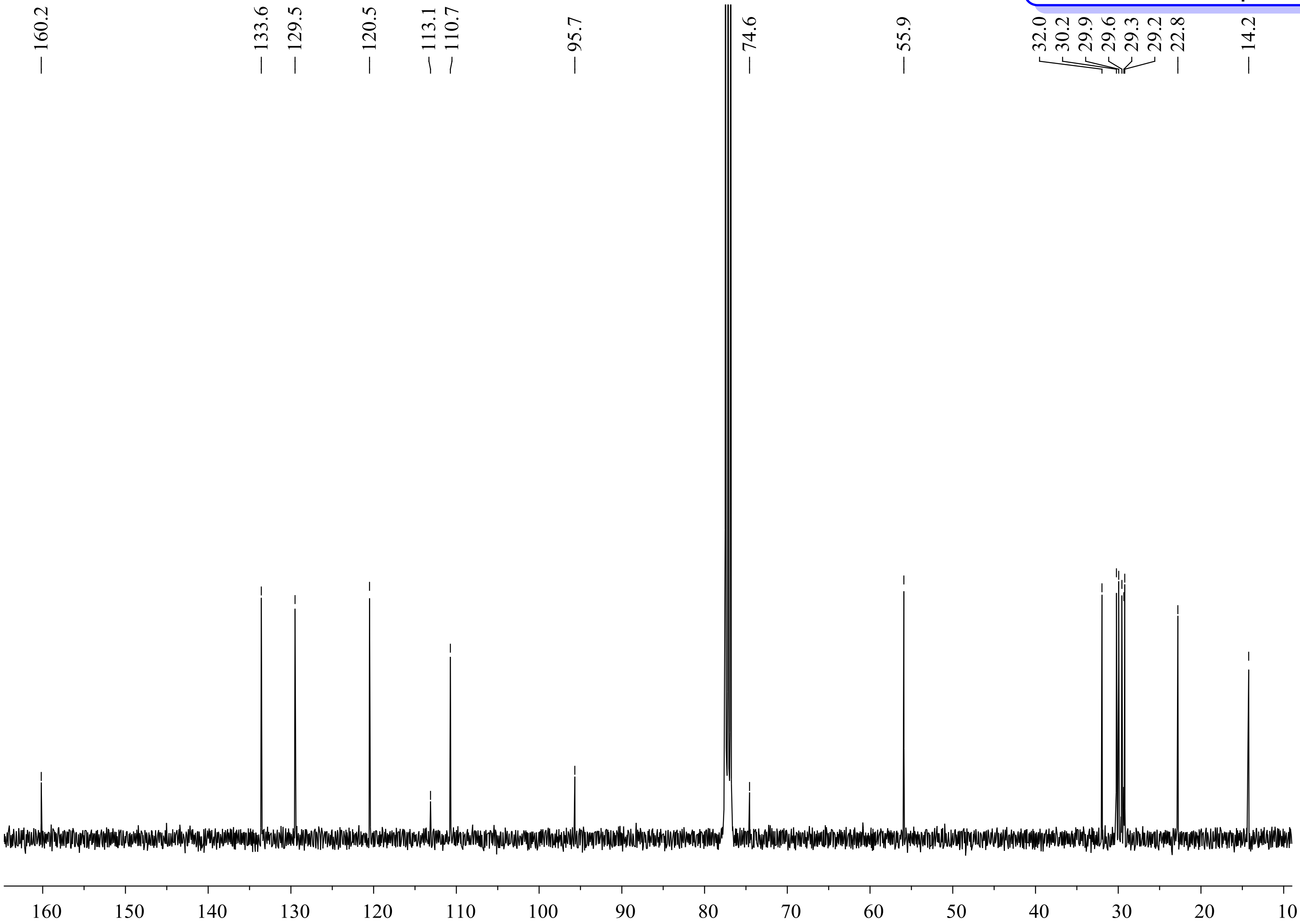

^{77}Se NMR. ((2-Methoxyphenyl)ethynyl)(*n*-octyl)selane (5q)

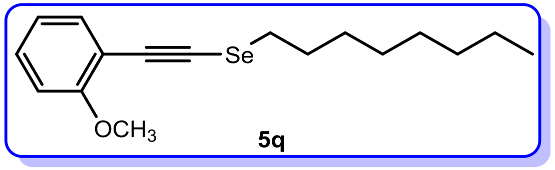

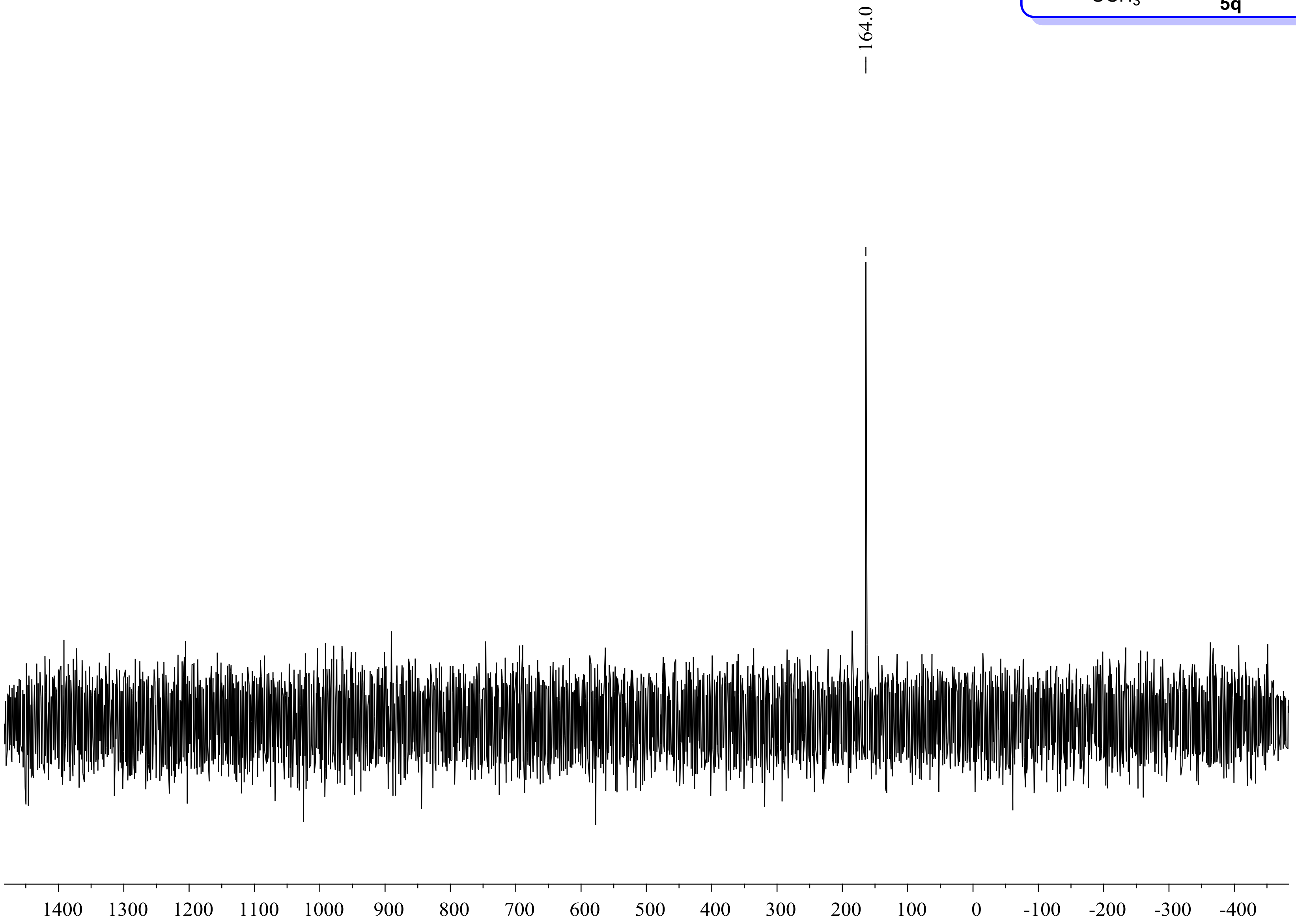

^{1}H NMR. 1-Ethyl-2-(*n*-octylselanyl)-1*H*-indole (9)

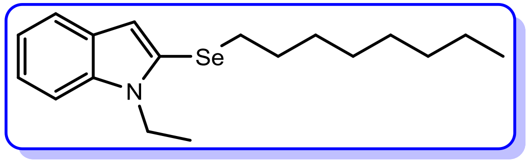

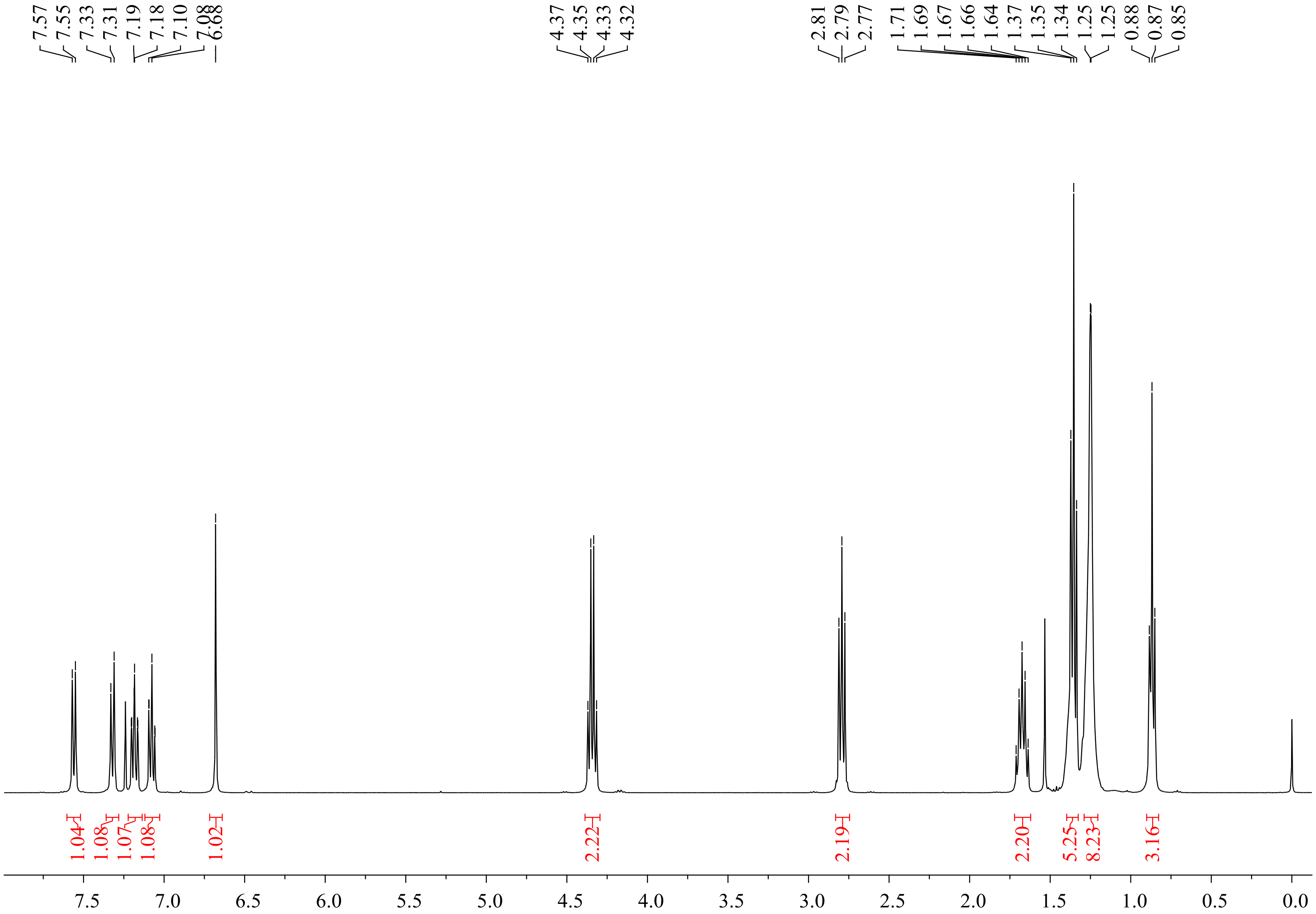

^{13}C NMR. 1-Ethyl-2-(*n*-octylselanyl)-1*H*-indole (9)

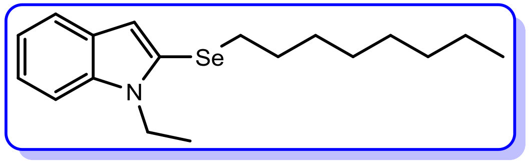

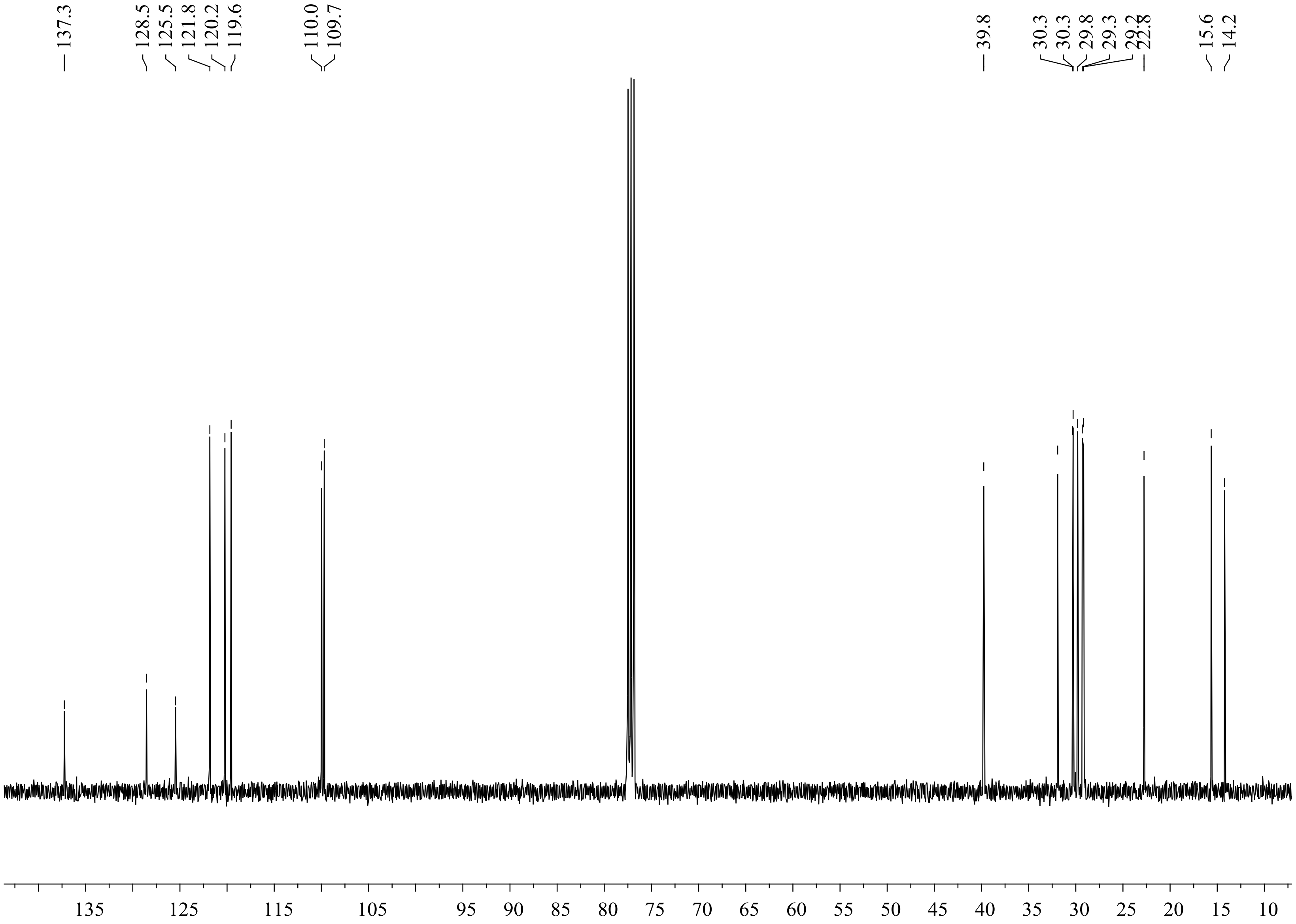

^{77}Se NMR. 1-Ethyl-2-(*n*-octylselanyl)-1*H*-indole (9)

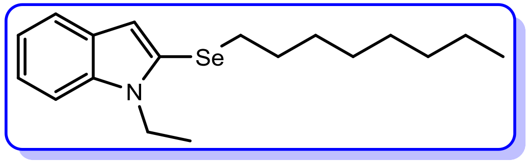

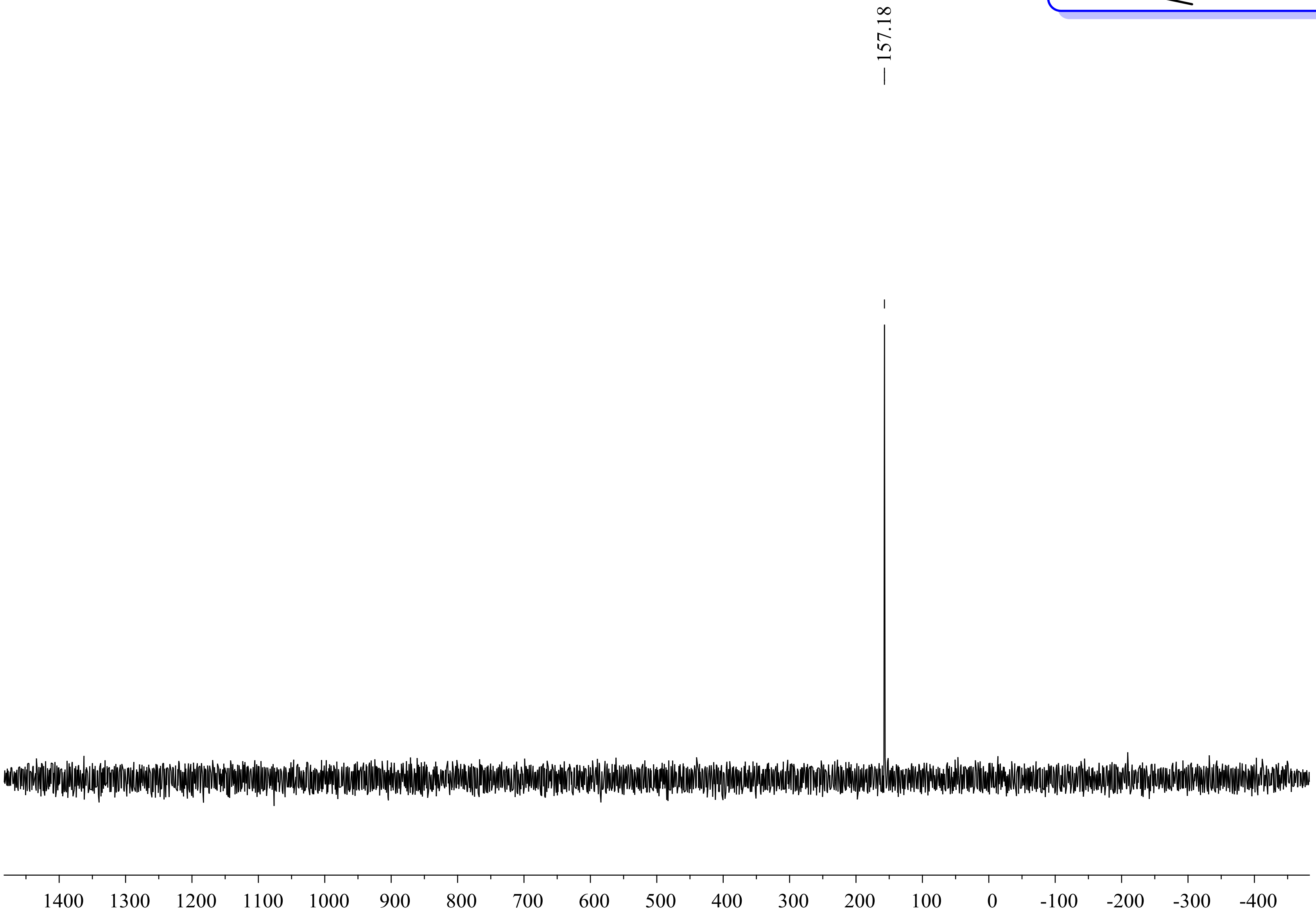

^{1}H NMR. 3-Iodo-2-(*n*-octylselanyl)benzofuran (10)

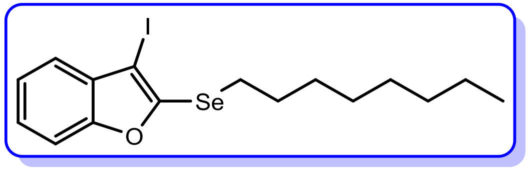

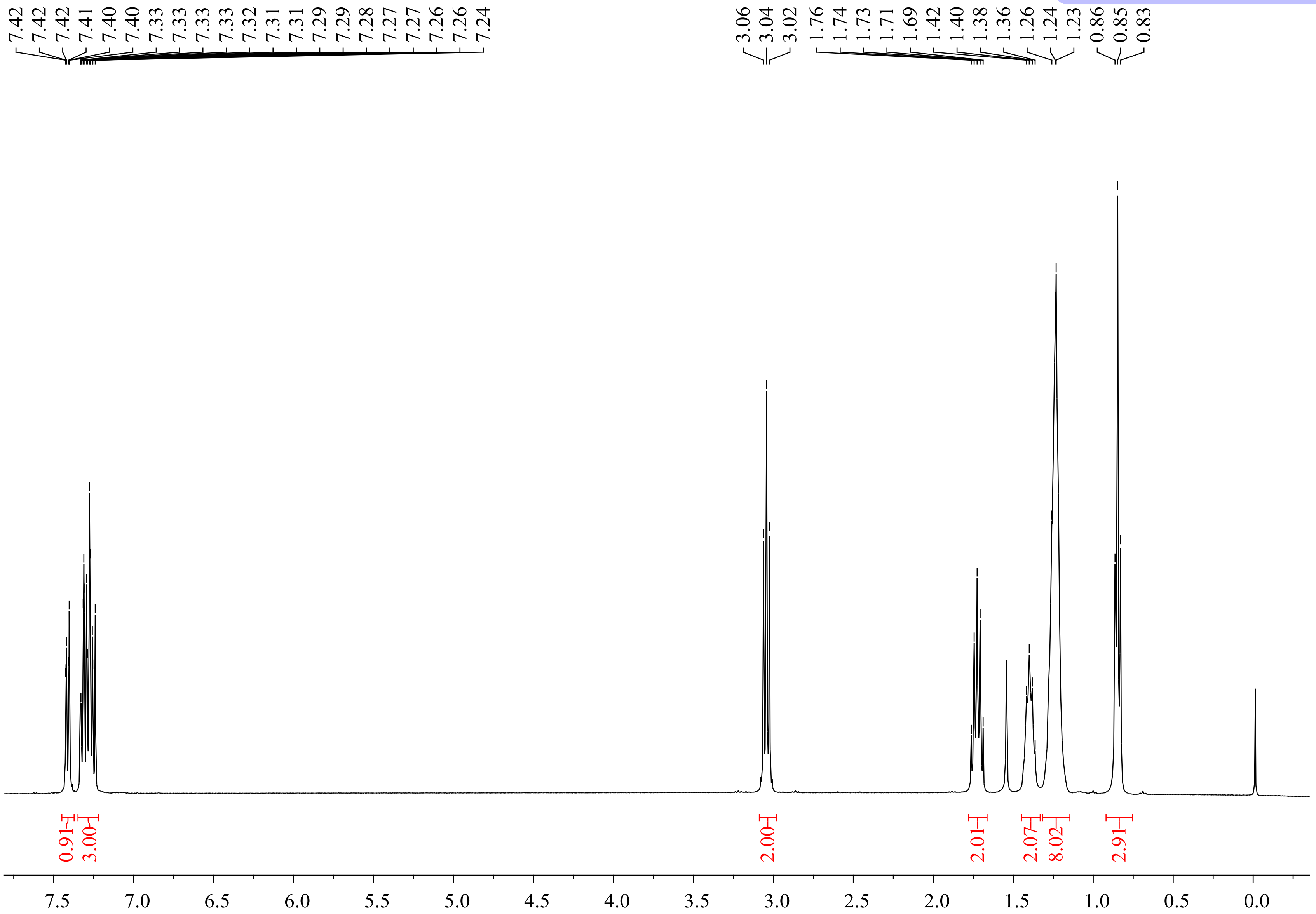

^{13}C NMR. 3-Iodo-2-(*n*-octylselanyl)benzofuran (10)

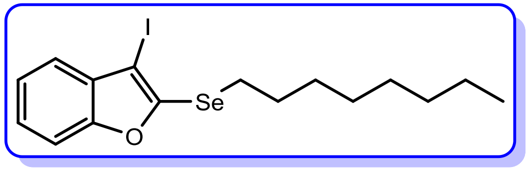

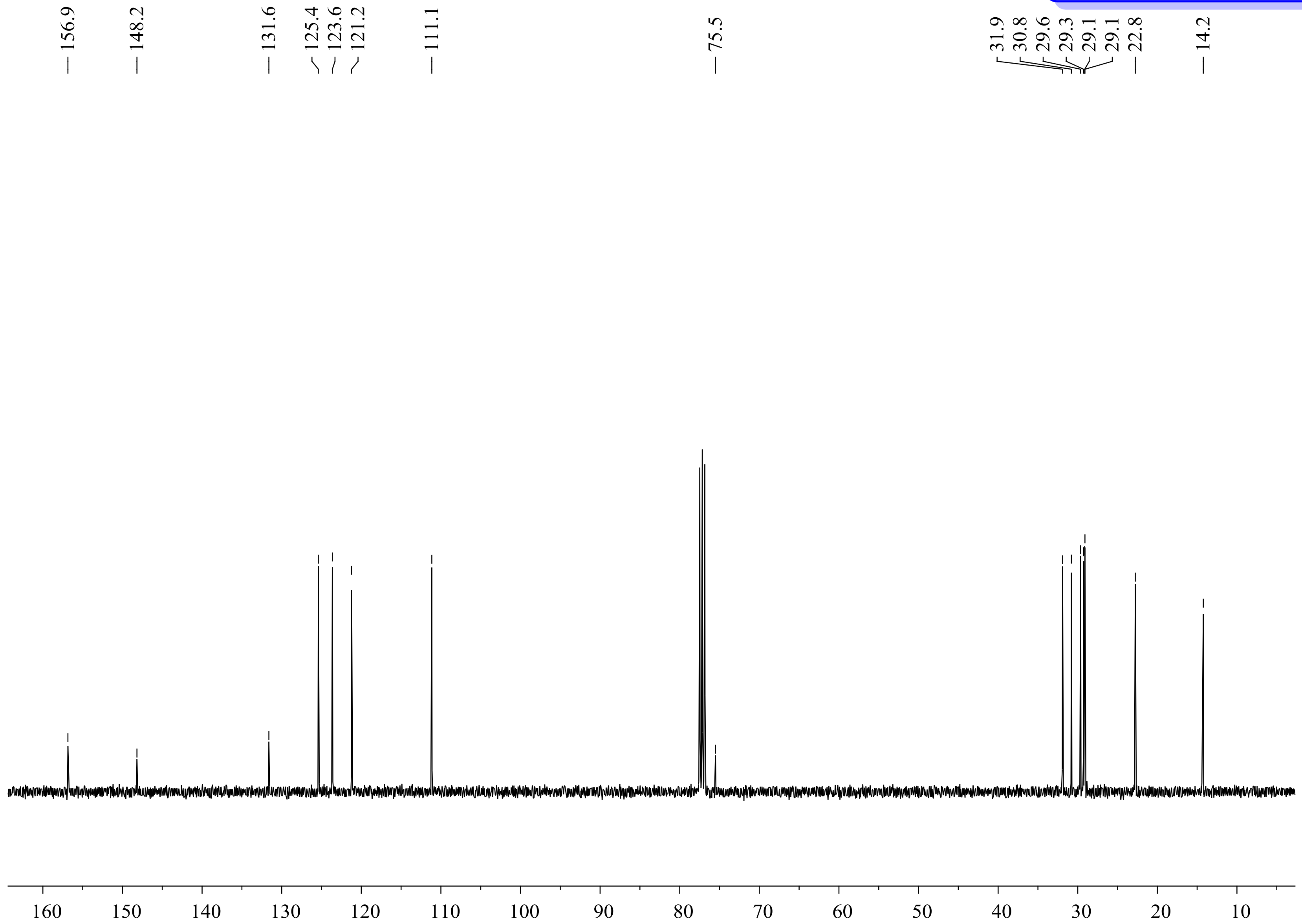

^{77}**Se NMR. 3-Iodo-2-(*n*-octylselanyl)benzofuran (10)**

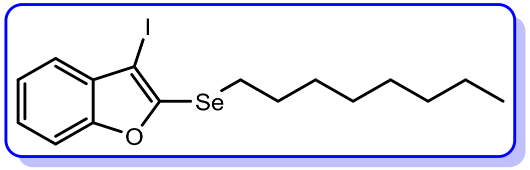

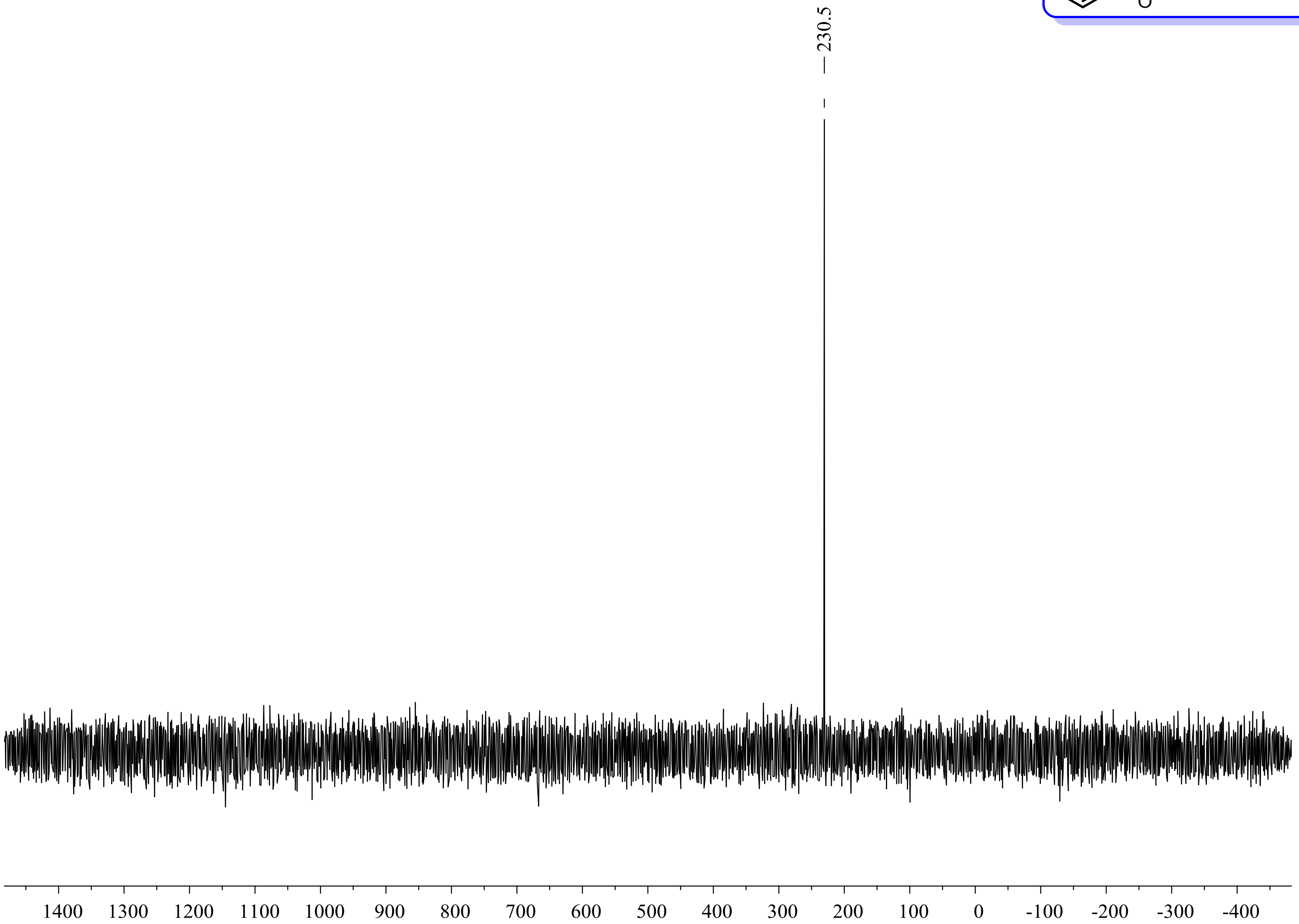

Supplement: File 1 — Experimental details, characterization data and copies of 1H, 13C and 77Se NMR spectra for products 5a–e, 5i–l, 5n, 5q, 9 and 10. [file Beilstein_J_Org_Chem-13-910-s001.pdf]
